# Total Synthesis of (−)-Neocucurbol C Enabled by Pattern Recognition and MHAT Cyclization

**DOI:** 10.1021/jacs.5c08224

**Published:** 2025-08-04

**Authors:** Li-Ping Zhong, Cyrus Gudeman, Jingsong Zhen, Oshani A. Wanasinghe, Jacob Hellmig, Michael J. E. Collins, John Bacsa, Alexander Adibekian, Mingji Dai

**Affiliations:** † Department of Chemistry, 1371 Emory University, Atlanta, Georgia 30322, United States; ‡ Department of Chemistry, 14681 University of Illinois Chicago, Chicago, Illinois 60607, United States; § University of Illinois Cancer Center, Chicago, Illinois 60607, United States; ∥ Department of Pharmacology and Chemical Biology, School of Medicine, 1371 Emory University, Atlanta, Georgia 30322, United States

## Abstract

We report an asymmetric total synthesis of (−)-neocucurbol C, a diterpene natural product possessing a unique and complex 6/6/5/5/6 polycyclic skeleton and nine stereocenters. Pattern-recognition analysis led us to the chiral pool molecule (+)-nootkatone as the starting material, already containing the AB ring system and two key stereocenters encoded by the target molecule. The extra isopropenyl group of (+)-nootkatone was removed by Kwon’s hydrodealkenylative bond fragmentation. Other key steps include a Suzuki–Miyaura cross coupling to introduce an aromatic ring as the E ring precursor, an oxidative dearomatization cyclization to form the key oxa-bridge, and a metal-catalyzed hydrogen atom transfer (MHAT)-initiated reductive radical cyclization to complete the entire framework for subsequent peripheral decorations, which eventually delivered (−)-neocucurbol C in 24 steps for the first time. In addition, the cytotoxicity evaluation of (−)-neocucurbol C and its synthetic intermediates against multiple cancer cell lines identified new lead compounds with promising anticancer activity for further development.

## Graphical abstract

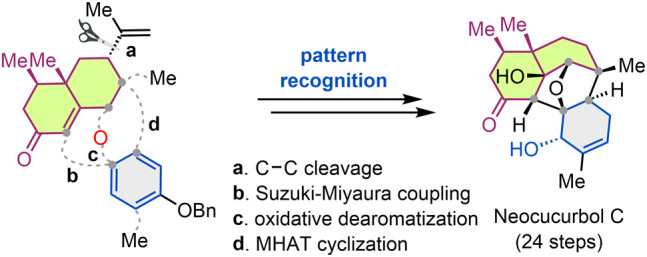

The neocucurbols (**1**–**5**, Figure 1A) are produced by *Neocucurbitaria unguis-hominis* FS685, a strain originating from a deep-sea sediment sample. They were isolated by Zhang and co-workers in 2022 and characterized as novel phomactin diterpenes.^1^ Among them, neocucurbols A–D represent the first phomactin family^2^ members with a complex and unprecedented 6/6/5/5/6 polycyclic skeleton. Three bridged ring systems (a bicyclo[5.3.1]­undecane, a bicyclo[3.3.1]­nonane, and an oxabicyclo[2.2.1]­heptane) and eight or nine stereocenters are embedded in their remarkable framework. In addition, four other neocucurbols (E–H) featuring a unique 6/8/6 tricyclic carbon skeleton (cf. **5**) were identified from the same extract. Biosynthetically, the neocucurbols are believed to derive from geranylgeranyl pyrophosphate (**8**, Figure 1B) by going through phomactatriene (**9**). Double-bond isomerization followed by protonation could convert **9** to allylic cation intermediate **10** for a transannular cyclization to generate **11** with the same carbon skeleton of neocucurbols E–H. From **11**, we proposed a remote proton elimination^3^ to forge the C9–C16 bond and deliver the tetracyclic carbon skeleton of neocucurbols A–D.

**Figure 1:**
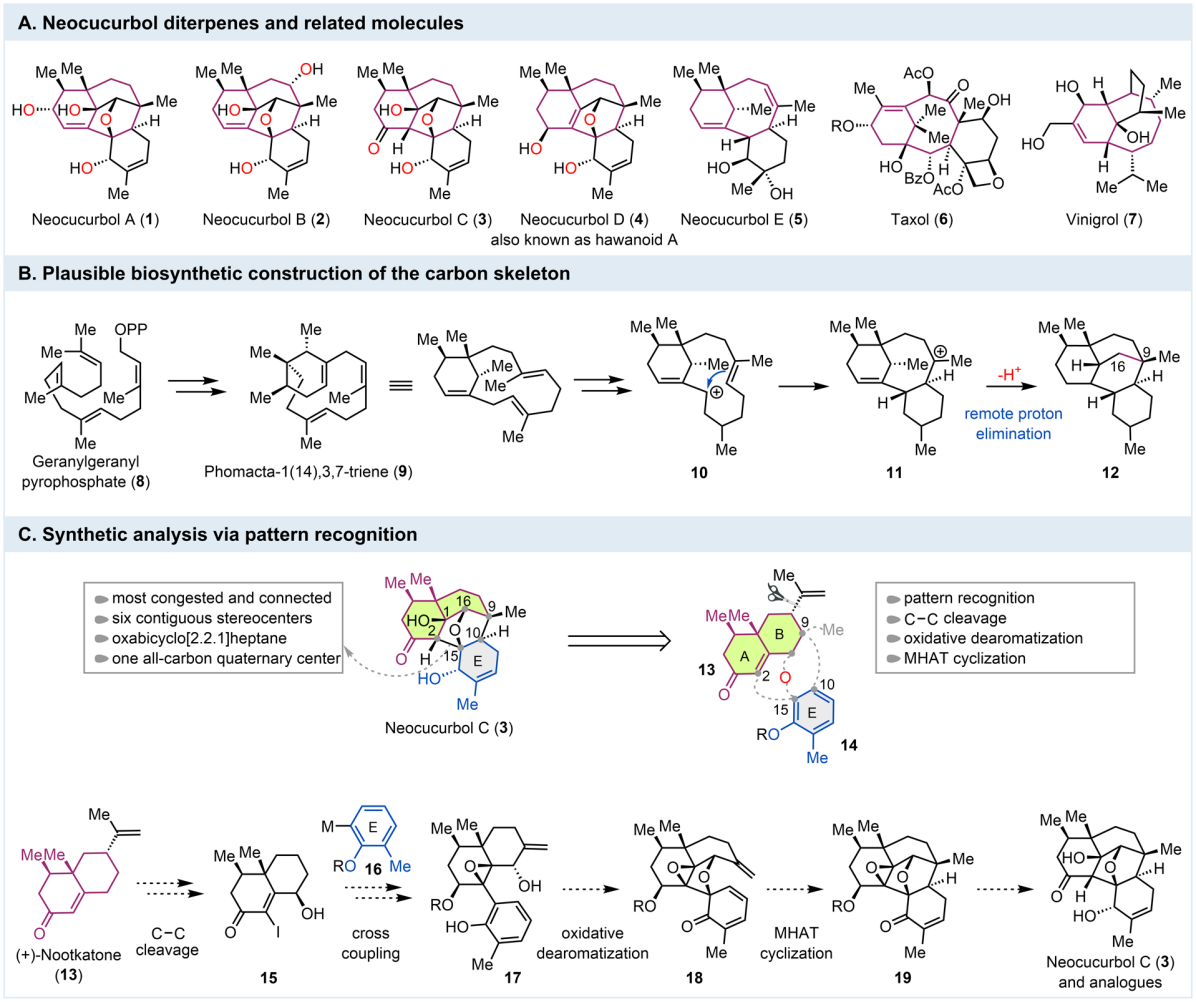
Structure, Plausible Biosynthesis, and Synthetic Plan.

Their polycyclic carbon skeleton and multiple stereocenters render the neocucurbols challenging targets for total synthesis and offer opportunities for innovations in chemistry. The bicyclo[5.3.1]­undecane scaffold of the neocucurbols resembles the ones found in taxol (**9**)^4^ and vinigrol (**10**),^5^ two challenging natural products with a lot of synthetic attention. So far, no total synthesis of neocucurbols has been reported. Herein, we report the first asymmetric total synthesis of neocucurbol C (**3**).

Using pattern-recognition analysis,^6^ we mapped (+)-nootkatone (**13**) on the AB ring system of neocucurbol A–D (Figure 1C). This would require a selective C–C bond cleavage to amputate the isopropenyl group of (+)-nootkatone (**13 → 15**). Such surgical removal of the isopropenyl group was recently developed by Kwon and co-workers and has significantly expanded the application of chiral pool molecules.^7^ With this in mind and using bond-network analysis,^8^ we decided to stitch the E ring onto the decalin ring derived from (+)-nootkatone by constructing the oxabicyclo[2.2.1]­heptane, the most congested ring system of neocucurbols A–D. We further envisioned a substituted benzene (cf. **16**) as the E ring precursor, which would allow a cross coupling such as the Suzuki–Miyaura reaction^9^ to form the C2–C15 bond and link the aromatic moiety to the decalin system (**15 → 17**). A subsequent oxidative dearomatization^10^ was planned to break the aromaticity and form the C15–O bond (**17 → 18**). How to form the C9–C10 bond became the most challenging step. We wondered about the possibility of using a reductive olefin coupling via a metal-catalyzed hydrogen atom transfer (MHAT)-initiated radical cyclization^11^ to form this strategically important bond and the C9 quaternary center, delivering the entire framework of neocucurbols A–D (**18 → 19**). From here on, several peripheral manipulations would be needed to complete the total synthesis. With this said, we were aware of several potential issues associated with this synthetic plan. One major issue is the stereochemical control of the spirocenter formed in the oxidative dearomatization cyclization step. There is no convincing reason that one stereoisomer would be significantly favored over the other one. Second, while there are ample precedents for MHAT cyclization at the β-position of α,β-unsaturated enones, cyclization at the δ-position to form a bridged ring system poses a great synthetic challenge.

With the above concerns, we decided to carry out a model study (Scheme 1A) and prepared **20** (see Supporting Information) to evaluate the two key cyclization steps. When **20** was treated with PIDA in HFIP,^12^ we did not observe any desired product. Instead, compound **21** and its epimer were produced in 73% yield (d.r. = 2.5/1), slightly favoring **21** presumably due to the directing effect of the tertiary alcohol. While the oxidative dearomatization cyclization did occur, the diastereoselectivity was low. What made it worse is that an iodophenyl derived from PIDA was added at the γ-position.^13^ Various reaction conditions and hypervalent iodine reagents were investigated to suppress the formation of these byproducts but without any success. While we could not prevent the formation of **21**, we decided to use it as a probe molecule to test the proposed MHAT cyclization. Again, our endeavors were not fruitful. At this stage, we decided to move the hydroxyl group on the aromatic ring from the *ortho* position to the *para* position. While such a maneuver would increase the steps for late-stage E ring functionalization, it could avoid the addition of the iodophenyl group and simplify the diastereoselectivity issue. The β-position of the oxidative dearomatization product (a cyclohexa-2,5-dien-1-one) would also be more reactive than the δ-position of the corresponding cyclohexa-2,4-dien-1-one for the MHAT cyclization. Model **23** was then prepared to test this new strategy. When **23** was treated with PIDA in HFIP, the desired product **24** was produced in 60% yield. Furthermore, **23** underwent MHAT cyclization to give **25** (CCDC 2450485) in 75% yield with the combination of Fe­(acac)_3_ and PhSiH_3_.^14^

**Scheme 1:**
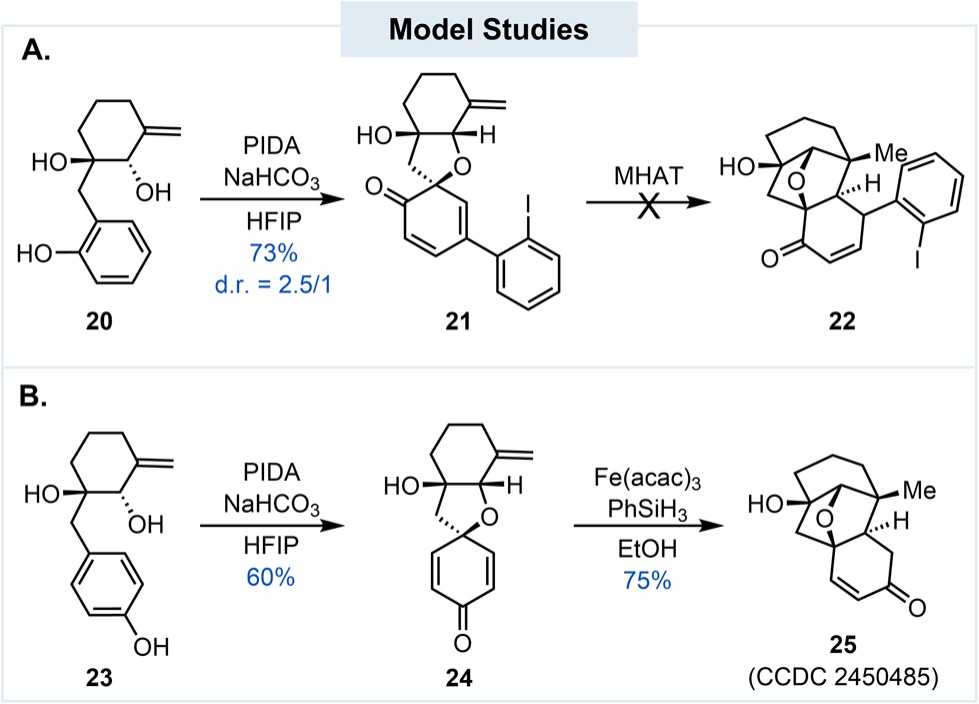
Two Model Studies

With a successful model, we started our total synthesis from commercially available (+)-nootkatone (Scheme 2). Kwon’s hydrodealkenylative bond fragmentation was utilized to remove the extra isopropenyl group, delivering **26** in 85% yield on a gram scale.^15^ We then investigated different protocols to introduce a hydroxyl group at the γ-position of the enone. While Yue’s photoredox C–H hydroxylation was able to introduce the hydroxyl group in one step,^16^ we could not scale up this synthesis to gram scale and the reaction was complicated by overoxidation (see the Supporting Information). Thus, we opted for a well-established two-step procedure: extended silyl enol ether formation and *m*-CPBA oxidation,^17^ which produced **27** and its epimer in 81% yield (d.r. = 4.4/1) on a gram scale. At this stage, we decided to introduce an iodide at the α-position of the enone for subsequent cross-coupling to append the aromatic moiety. This α-iodination turned out to be difficult due to the steric interference generated by the angular all-carbon quaternary center. Our initial attempts with the Johnson iodination protocol (I_2_, pyridine) were unsuccessful.^18^ We were able to circumvent this problem with a combination of TMSN_3_ and iodine.^19^ In this case, TMSN_3_ functions as a Lewis acid to activate the enone, and the azide is a relatively small nucleophile for the conjugate addition step. Under these iodination conditions, the secondary alcohol was also protected as TMS ether to give products **28** and **29**. While the yield for the desired product **28** was modest (30%), **29** could be converted back to **27** with TBAF for the next cycle to avoid significant material loss. Subsequent Suzuki–Miyaura cross coupling between **28** and boronic acid **30** occurred smoothly with Pd­(dppf)­Cl_2_ as catalyst.^20^ The coupling product was next reduced to **31** with a Luche reduction. After a one-pot acetate protection and TMS group removal, **31** was then converted to **32** for a directed epoxidation with VO­(acac)_2_ and TBHP. Notably, the resulting fully substituted epoxide is surprisingly stable and survived the following steps until the desired reductive opening. Dess-Martin periodinane (DMP) oxidation of the secondary alcohol gave ketone **33**. The next step was to introduce the desired α-methylene group, which was accomplished using *N,N,N′,N′*-tetramethanediamine as a mild Mannich reagent.^21^ The ketone was then stereoselectively reduced in the same pot to furnish **34** in 80% yield. Subsequent benzyl removal with BBr_3_ set the stage for the oxidative dearomatization cyclization. Notably, the free phenol product is unstable, presumably due to *para* quinone methide formation via epoxide opening, and needs to be subjected to the next step immediately. Upon treatment of the crude phenol with PIDA in HFIP, the desired product **35** was produced in 68% yield from **34**. Compound **35** was then treated with Fe­(acac)_3_ and PhSiH_3_ to initiate the reductive radical cyclization, which produced **36** (CCDC 2424690) with the entire neocucurbol skeleton in 75% yield.

**Scheme 2:**
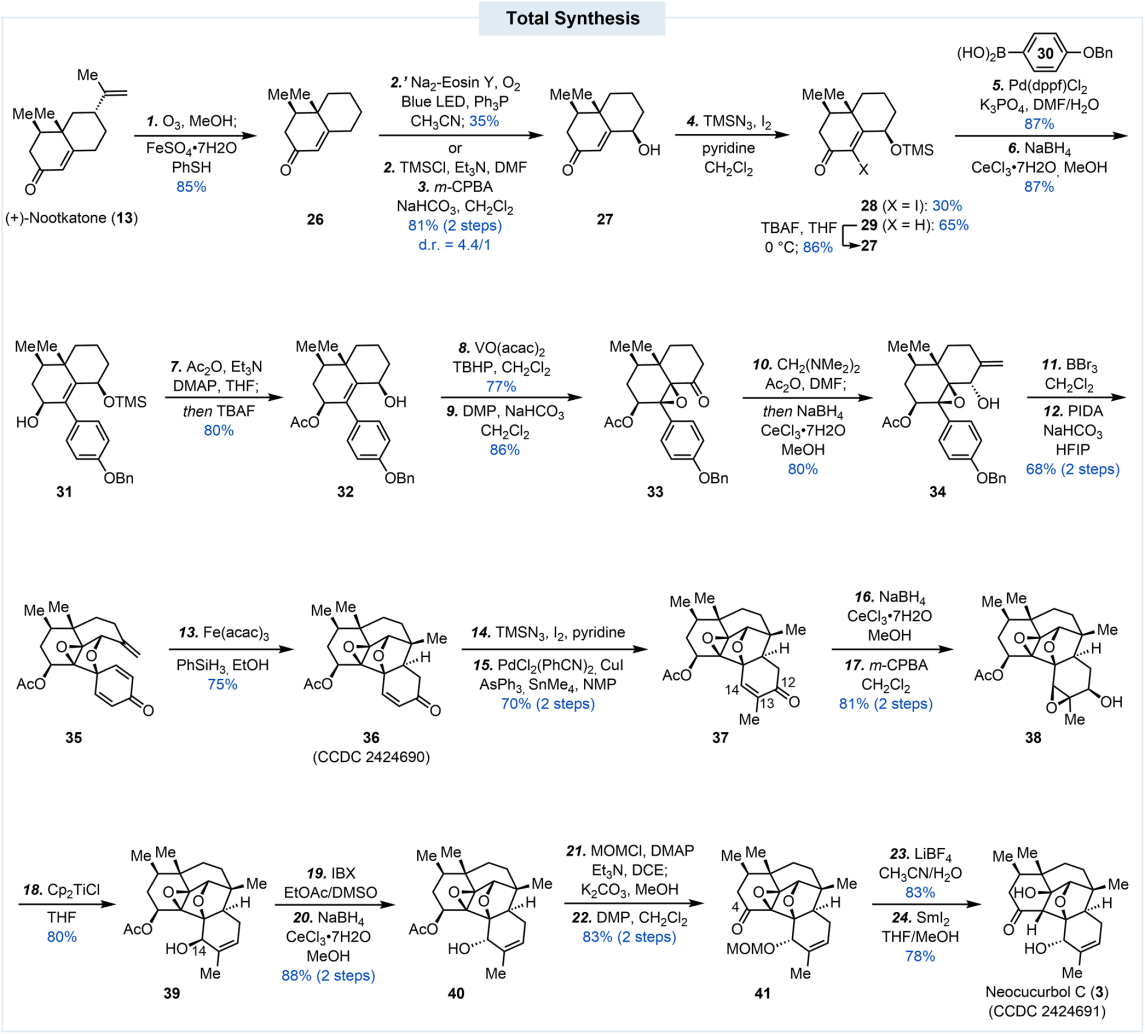
Total Synthesis of Neocucurbol C

With **36** in hand, peripheral decoration was needed to introduce one more methyl group and adjust the oxidation state. Installation of the C13 methyl group was accomplished by α-iododination followed by Stille cross coupling.^22^ This two-step process afforded **37** in 70% yield. We next needed to migrate the oxygen functionality from C12 to C14, an issue resulting from moving the phenol from the *ortho* to *para* position to enable a successful oxidative dearomatization and MHAT cyclization. This maneuver turned out to be nontrivial. To achieve this transposition, the ketone group was first reduced to a secondary alcohol to direct subsequent *m*-CPBA epoxidation to give **38** in 81% yield. Compound **38** was then subjected to a titanocene-promoted reductive elimination^23^ to afford **39** with undesired stereochemistry at C14, which was fixed with a two-step process: IBX oxidation and Luche reduction. Notably, the stereochemistries of the epoxide and the secondary alcohol of **38** are important for the titanocene-promoted epoxyl alcohol reductive elimination. To complete the total synthesis of neocucurbol C, the secondary alcohol of **40** was protected as a MOM ether. In the same pot, the acetate group at C4 was hydrolyzed and the resulting alcohol was oxidized to ketone **41** with DMP. After removal of the MOM protecting group with LiBF_4_, SmI_2_-mediated reductive epoxide opening delivered neocucurbol C in 78% yield. In addition to comprehensive NMR and mass spectral analyses, we further confirmed the structure and absolute stereochemistry of our synthetic neocucurbol C by X-ray crystallographic analysis (CCDC 2424691). Notably, the optical rotation data of our neocucurbol C sample is −18 (*c* 0.1 in MeOH), which is not consistent with the data [+28 (*c* 0.1 in MeOH)] reported in the isolation paper.^1a^ The absolute stereochemistry of natural neocucurbol C was also established by X-ray crystallographic analysis, which is consistent with our assignment. In addition, the electronic circular dichroism (ECD) spectrum of our synthetic sample matches the ECD spectrum of the natural one. Without access to the natural sample, we currently do not know what caused the optical rotation difference. Since the natural sample contained a significant amount of impurities based on the reported NMR spectra, the differences are likely generated by these unknown impurities.^24^

While neocucurbol C and its natural congeners were reported to have no cytotoxicity against several human cancer cell lines,^1a^ we wondered whether the synthetic intermediates generated in our synthesis have any biological activities. Neocucurbol C together with 10 synthetic intermediates were evaluated against a panel of carcinoma cell lines (see the Supporting Information). While neocucurbol C did not show cytotoxicity against these carcinoma cell lines, compounds **35** and **36** exhibited promising cytotoxicity with EC_50_ values ranging from 1.64 μM to 37.84 μM (Table 1).

**Table 1:** EC_50_ Values of **35**, **36**, and Neocucurbol C (**3**)

Cell line	**35** (μM)	**36** (μM)	**3** (μM)
A431	13.24 ± 2.68	14.06 ± 3.40	ND^a^
HeLa	13.74 ± 3.23	18.67 ± 2.07	ND
Caco-2	21.90 ± 2.48	37.84 ± 2.64	ND
MDA-MB-231	13.66 ± 1.44	16.84 ± 1.09	ND
A375-MA2	3.71 ± 2.01	1.64 ± 0.40	>50
Ishikawa	4.71 ± 0.99	4.73 ± 1.37	>50
Huh7	20.72 ± 6.90	22.88 ± 4.89	>50
HCT116	3.74 ± 3.38	6.39 ± 4.49	>50

a ND: not determined.

In summary, we completed the first total synthesis of neocucurbol C in 24 steps. Pattern-recognition analysis enabled us to identify (+)-nootkatone as a readily available starting material, the unnecessary isopropenyl group of which was trimmed off with Kwon’s hydrodealkenylative bond fragmentation. An aromatic starting material was used as the E ring precursor and appended on the AB ring system with Suzuki–Miyaura cross coupling. The most connected oxabicyclo[2.2.1]­heptane ring was subsequently constructed with an oxidative dearomatization, followed by a MHAT reductive cyclization. With these enabling transformations, the polycyclic skeleton of neocucurbol C was established in 13 steps. While the following peripheral decoration took a longer sequence than ideal, it produced synthetic intermediates with the neocucurbol skeleton for biological evaluation, from which compound **36** and its precursor **35** were identified to show promising anticancer activity and are worth further investigation. Chemoproteomic studies to identify the cellular targets of these compounds, including neocucurbol C, are currently underway and will be reported in due course.

## Supplementary Material



## Notes

Experimental procedures and NMR spectra for all new compounds (PDF)

## ABBREVIATIONS

HFIP: hexafluoroisopropanol
PIDA: phenyliodine­(III) diacetate
TMS: trimethylsilyl
DMF: dimethylformamide
*m*-CPBA: *meta*-chloroperoxybenzoic acid
DMAP: 4-dimethylaminopyridine
THF: tetrahydrofuran
TBAF: tetra-*n*-butylammonium fluoride
TBHP: *tert*-butyl hydroperoxide
DMP: Dess-Martin periodinane
NMP: *N*-methyl-2-pyrrolidone
IBX: 2-iodoxybenzoic acid
DMSO: dimethyl sulfoxide
MOMCl: methoxymethyl chloride
DCE: 1,2-dichloroethane.
