# Supplementary material for: Total Synthesis of (−)-Neocucurbol C Enabled by Pattern Recognition and MHAT Cyclization
Source: J Am Chem Soc. 2025 Aug 4;147(32):28589–94. doi: 10.1021/jacs.5c08224 (PMC12356577; doi:10.1021/jacs.5c08224)
Supplement: Supplementary file 1 [file ja5c08224_si_001.pdf]

Supporting Information

Total Synthesis of (−)-Neocucurbol C Enabled by Pattern Recognition and MHAT Cyclization

Li-Ping Zhong,[1] Cyrus Gudeman,[1] Jingsong Zhen,[1] Oshani A. Wanasinghe[2], Jacob Hellmig,[1] Michael J. E. Collins,[1] John Bacsa,[1] Alexander Adibekian,*[,2,3] and Mingji Dai*[,1,4]

[1]Department of Chemistry, Emory University, Atlanta, Georgia 30322, United States

[2]Department of Chemistry, University of Illinois Chicago, Chicago, Illinois 60607, United Sates

[3]University of Illinois Cancer Center, Chicago, Illinois 60607, United Sates

[4]Department of Pharmacology and Chemical Biology, School of Medicine, Emory University, Atlanta, Georgia 30322, United States

Table of Contents

Part 1. Experimental procedures and spectra data..S3

Part 2. NMR comparison..S30

Part 3. X-ray structures and analysis data..S34

Part 4. Biological evaluations…………...S37

Part 5. References..S41

Part 6. ^{1}H and ^{13}C NMR spectra..S42

Part 1. Experimental procedures and spectra data

A. General Methods

All commercially available compounds were purchased from Sigma-Aldrich, Alfa-Aesar, Oakwood chemicals and Ambeed unless otherwise noted. Materials obtained from commercial suppliers were used without further purification. NMR spectra were recorded on Bruker spectrometers (^{1}H at 400 MHz, 600 MHz, 800 MHz and ^{13}C at 101 MHz, 151 MHz, 201 MHz). Chemical shifts (δ) were given in ppm with reference to solvent signals [$CDCl_3$ δ 7.26 ppm ^{1}H NMR, δ 77.16 ppm ^{13}C NMR); CD_3OD, δ 3.31 ppm ^{1}H NMR, δ 49.0 ppm ^{13}C NMR]. ^{1}H NMR data are reported as follows: chemical shift (δ ppm), multiplicity (s = singlet, d = doublet, t = triplet, q = quartet, m = multiplet, br = broad), coupling constant (Hz), and integration. IR spectra were collected on a Nicolet iS10 FT-IR spectrometer. Mass spectra were taken on a Thermo Finnigan LTQ-FTMS spectrometer with APCI, ESI. Optical rotation were determined by Autopol IV(Rudolph Research Analytical). Column chromatography was performed on silica gel. All reactions sensitive to air or moisture were conducted under argon atmosphere in dry solvents under anhydrous conditions, unless otherwise noted. Dry THF (tetrahydrofuran), DMF (dimethylformamide), MeCN and DCM (CH_2Cl_2) were processed via PureProcessTechnology GS-SPS-5-CM system. All other solvents and reagents were used as obtained from commercial sources without further purification. Room temperature (r.t.) is around 23 °C.

B. Model Study

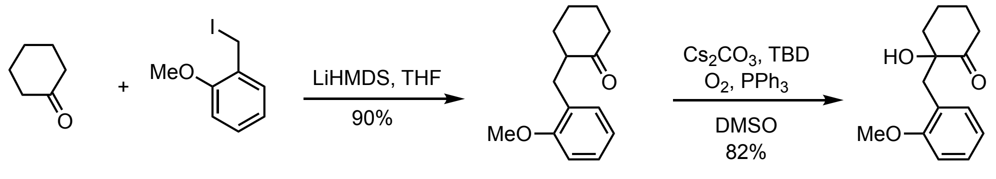

Cyclohexanone **S1** (2.0 g, 20.4 mmol, 1.0 equiv.) was dissolved in THF (20 mL). LiHMDS (1.0 M in THF, 20.4 mL, 20.4 mmol, 1.0 equiv.) was added to the solution at −78 °C. After the solution was stirred at this temperature for 30 min, **S2**[1] (5.05 g, 20.4 mmol, 1.0 equiv.) in THF (6.0 mL) was added at the same temperature. The mixture was warmed to room temperature and stirred for another 3 h. The reaction mixture was quenched with saturated aqueous NH_4Cl (30 mL). The layers were separated and the aqueous layer was extracted with EtOAc (20 mL × 3). The combined organic layers were washed with brine (50 mL),

dried over Na_2SO_4, filtered and concentrated under reduced pressure. The crude product was purified by flash chromatography (5~15% ethyl acetate in hexane) to yield **S3** (4.0 g, 90%) as a colorless oil.

Under a balloon pressure of oxygen, the mixture of **S3** (4.0 g, 18.4 mmol, 1.0 equiv.) and Cs_2CO_3 (1.79 g, 5.51 mmol, 0.3 equiv.), TBD (1,5,7-triazabicyclo[4.4.0]dec-5-ene, 0.767 g, 5.51 mmol, 0.3 equiv.), PPh_3 (5.80 g, 22.1 mmol, 1.2 equiv.) in DMSO (150 mL) was stirred for 15 h at room temperature.[2] After TLC showed full consumption of **S3**, the reaction mixture was quenched with saturated aqueous NH_4Cl (150 mL). The layers were separated and the aqueous layer was extracted with Et_2O (100 mL × 3). The combined organic layers were washed with brine (200 mL), dried over Na_2SO_4, filtered and concentrated under reduced pressure. The crude product was purified by flash chromatography (10~25% ethyl acetate in hexane) to yield **S4** (3.5 g, 82%) as a colorless oil.

R_f= 0.5 (hexane/ethyl acetate = 4/1).
IR (film) λ_{max}: 3515, 2933, 2860, 1705, 1587, 1586, 1491, 1241, 1120, 1062, 1024, 749 cm^{-1}.
^{1}H NMR (400 MHz, $CDCl_3$) δ 7.21 (ddd, J = 13.7, 7.5, 1.8 Hz, 2H), 6.94 – 6.85 (m, 1H), 6.80 (d, J = 8.1 Hz, 1H), 3.73 (s, 3H), 3.69 (s, 1H), 3.50 (d, J = 13.6 Hz, 1H), 2.94 (td, J = 13.8, 6.2 Hz, 1H), 2.76 (d, J = 13.6 Hz, 1H), 2.57 – 2.46 (m, 1H), 2.27 – 2.05 (m, 2H), 1.89 (tdd, J = 13.8, 11.9, 6.6 Hz, 2H), 1.78 – 1.64 (m, 2H).
^{13}C NMR (101 MHz, $CDCl_3$) δ 213.2, 157.3, 133.4, 128.3, 123.9, 120.5, 110.3, 78.8, 54.9, 41.6, 38.5, 38.4, 27.9, 22.9.
HRMS m/z (APCI): calc. for $C_{14}H_{17}O_2^+$ $[M+H–H_2O]^+$: 217.1223, found: 217.1222.

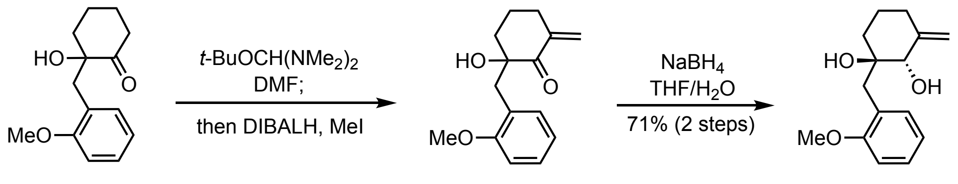

S4 S5 S6

Compound **S4** (2.0 g, 8.55 mmol, 1.0 equiv.) was dissolved in DMF (30 mL. t-BuOCH$(NMe_2)_2$ (8.9 mL, 42.8 mmol, 5.0 equiv.) was added to the solution. The mixture was then heated to 100 °C and stirred for 1 h.[3] After TLC showed full consumption of **S4**, the reaction mixture was cooled down to room temperature and concentrated under reduced pressure to give the crude enamine, which was used for next step without purification.

The above crude residue was dissolved in CH_2Cl_2 (50 mL). DIBALH (1.0 M in hexanes, 19.7 mL, 19.7 mmol, 2.3 equiv.) was added at −78 °C. The mixture was then warmed to room temperature and stirred for 30 min before MeI (1.23 mL, 19.7 mmol, 2.3 equiv.) was added. The stirring was continued for 1 h at the same temperature. The reaction was then cooled down to at −78 °C and quenched with a saturated aqueous sodium potassium tartarate (100 mL). The mixture was warmed to room temperature and stirred until the solution became clear. The layers were separeted, and the aqueous layer was extracted with DCM (50 mL × 2). The combined organic extracts were washed with brine (50 mL), dried over anhydrous Na_2SO_4, filtered and concentrated under reduced pressure. The crude product **S5** was used without further purification.

The crude product **S5** was dissolved in THF/H_2O (50 mL/5 mL). $NaBH_4$ (162 mg, 4.28 mmol, 0.5 equiv.) was added to the solution at 0 °C. The stirring was continued for 30 min at the same temperature. After TLC showed full consumption of **S5**, the reaction mixture was quenched with saturated aqueous Na_2CO_3 (20 mL). The layers were separeted, and the aqueous layer was extracted with DCM (30 mL × 3). The combined organic extracts were washed with brine (50 mL), dried over anhydrous Na_2SO_4, filtered and concentrated under reduced pressure. The crude product was purified by flash chromatography (25~33% ethyl acetate in hexane) to yield **S6** (1.51 g, 71%) as a yellow oil.

R_f= 0.5 (hexane/ethyl acetate = 2/1).
IR (film) λ_{max}: 3455, 2935, 2837, 1600, 1585, 1492, 1438, 1237, 1174, 1115, 1023, 753 cm^{-1}.
^{1}H NMR (400 MHz, $CDCl_3$) δ 7.29 – 7.16 (m, 2H), 6.99 – 6.86 (m, 2H), 4.97 (dd, *J* = 1.6, 1.6 Hz, 1H), 4.92 (dd, *J* = 2.2, 1.1 Hz, 1H), 3.87 (s, 3H), 3.85 – 3.81 (m, 1H), 3.18 (d, *J* = 14.6 Hz, 2H), 2.93 (d, *J* = 2.9 Hz, 1H), 2.69 (d, *J* = 14.3 Hz, 1H), 2.39 (dt, *J* = 12.8, 6.1 Hz, 1H), 2.16 – 2.02 (m, 1H), 1.80 (ddd, *J* = 12.6, 6.9, 3.8 Hz, 1H), 1.61 (tdd, *J* = 7.7, 5.4, 2.3 Hz, 2H), 1.51 – 1.40 (m, 1H).
^{13}C NMR (101 MHz, $CDCl_3$) δ 157.3, 147.4, 133.2, 128.1, 125.5, 121.3, 110.8, 109.6, 78.4, 76.5, 55.7, 34.7, 34.1, 31.8, 23.0.
HRMS m/z (APCI): calc. for $C_{15}H_{20}O_5^-$ $[M+O_2]^-$: 280.1316, found: 280.1319.

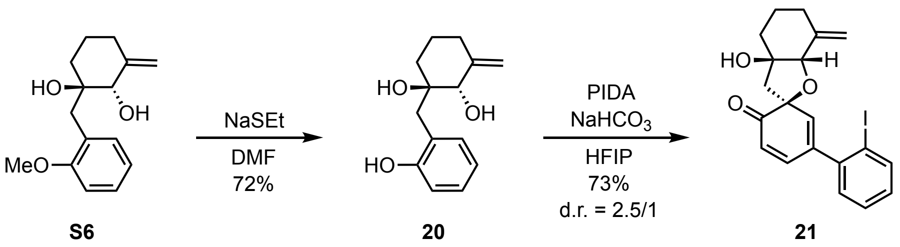

Compound **S6** (500 mg, 2.0 mmol, 1.0 equiv.) was dissolved in DMF (6 mL). To the stirred solution was then added Na_2SEt (672 mg, 8.0 mmol, 4.0 equiv.). The mixture was heated to 130 °C and stirred for 3 h. After TLC showed full consumption of **S6**, the reaction mixture was quenched with saturated aqueous NH_4Cl (20 mL). The layers were separeted, and the aqueous layer was extracted with Et_2O (20 mL × 2). The combined organic extracts were washed with brine (30 mL), dried over anhydrous Na_2SO_4, filtered and concentrated under reduced pressure. The crude product was purified by flash chromatography (15~33% ethyl acetate in hexane) to yield **20** (390 mg, 72%) as a yellow oil.

Compound **20** (50 mg, 0.212 mmol, 1.0 equiv.) was dissolved in HFIP (2.0 mL). $NaHCO_3$ (35.3 mg, 0.424 mmol, 2.0 equiv.) and PIDA (80.5 g, 0.25 mmol, 1.2 equiv.) were then added at 0 °C. The mixture was stirred for 20 min before it was quenched with saturated aqueous Na_2SO_3 (5.0 mL) and extracted with ethyl acetate (5.0 mL × 3). The combined organic layers were dried over anhydrous Na_2SO_4, filtered and concentrated *in vacuo.* The residue was purified by flash-column chromatography on silica gel (10~24% ethyl acetate in hexane) to afford **21** (67.5 mg, 73%) as a colorless oil.

R_f= 0.65 (hexane/ethyl acetate = 4/1).

IR (film) λ_{max}: 3441, 2932, 2863, 1711, 1461, 1430, 1265, 1264, 1093, 1044, 1013, 757, 734 cm^{-1}.

^{1}H NMR (600 MHz, $CDCl_3$) δ 7.89 (dd, *J* = 7.9, 1.2 Hz, 1H), 7.38 (td, *J* = 7.5, 1.3 Hz, 1H), 7.28 (dd, *J* = 7.6, 1.7 Hz, 1H), 7.04 (td, *J* = 7.7, 1.7 Hz, 1H), 6.95 (dd, *J* = 9.9, 2.3 Hz, 1H), 6.66 (d, *J* = 2.3 Hz, 1H), 6.06 (d, *J* = 9.9 Hz, 1H), 5.25 (s, 1H), 5.13 (s, 1H), 4.32 (s, 1H), 2.51 – 2.37 (m, 3H), 2.21 (t, *J* = 14.1 Hz, 2H), 1.73 – 1.56 (m, 4H).

^{13}C NMR (151 MHz, $CDCl_3$) δ 200.7, 143.7, 143.7, 143.4, 142.1, 139.5, 136.1, 129.8, 129.6, 128.7, 124.3, 115.9, 97.5, 86.7, 83.1, 80.2, 48.7, 35.5, 30.7, 22.9.

HRMS m/z (APCI): calc. for $C_{20}H_{20}IO_3^+$ $[M+H]^+$: 435.0452, found: 435.0451.

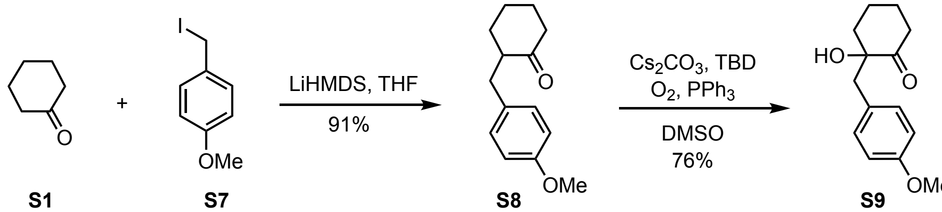

S1 S7 S8 S9

Cyclohexanone **S1** (2.0 g, 20.4 mmol, 1.0 equiv.) was dissolved in THF (30 mL). Then, LiHMDS (1.0 M in THF, 8.6 mL, 8.55 mmol, 1.0 equiv.) was added to the solution at −78 °C. The mixture was stirred at this temperature for 30 min before **S7**[4] (5.05 g, 20.4 mmol, 1.0 equiv.) in THF (6.0 mL) was added at the same temperature. The mixture was warmed to room temperature and stirred for another 3 h. The reaction mixture was quenched with saturated aqueous NH_4Cl (30 mL), The layers were separated and the aqueous layer was extracted with EtOAc (20 mL × 3). The combined organic layers were washed with brine (40 mL), dried over Na_2SO_4, filtered and concentrated under reduced pressure. The crude product was purified by flash chromatography (5~15% ethyl acetate in hexane) to yield **S8** (4.05 g, 91%) as a colorless oil.

Under a balloon pressure of oxygen, the mixture of **S8** (4.05 g, 18.6 mmol, 1.0 equiv.) and Cs_2CO_3 (1.81 g, 5.57 mmol, 0.3 equiv.), TBD (775 mg, 5.57 mmol, 0.3 equiv.), PPh_3 (5.85 g, 22.3 mmol, 1.2 equiv.) in DMSO (150 mL) was stirred for 15 h at room temperature. After TLC showed full consumption of **S7**, the reaction mixture was quenched with saturated aqueous NH_4Cl (150 mL). The layers were separated and the aqueous layer was extracted with Et_2O (100 mL × 3). The combined organic layers were washed with brine (200 mL), dried over Na_2SO_4, filtered and concentrated under reduced pressure. The crude product was purified by flash chromatography (10~25% ethyl acetate in hexane) to yield **S9** (3.3 g, 76%) as a colorless oil.

R_f= 0.5 (hexane/ethyl acetate = 4/1).

IR (film) λ_{max}: 3477, 2936, 2862, 1706, 1509, 1300, 1244, 1032, 833, 754, 658 cm^{-1}.

^{1}H NMR (400 MHz, $CDCl_3$) δ 7.11 (d, J = 8.6 Hz, 2H), 6.80 (d, J = 8.6 Hz, 2H), 3.84 (s, 1H), 3.77 (s, 3H), 3.09 (d, J = 14.0 Hz, 1H), 2.91 (d, J = 14.0 Hz, 1H), 2.67 (td, J = 13.7, 6.1 Hz, 1H), 2.58 – 2.47 (m, 1H), 2.18 (ddt, J = 13.1, 9.7, 3.1 Hz, 2H), 1.86 (dt, J = 7.3, 3.5 Hz, 2H), 1.77 – 1.58 (m, 2H).

^{13}C NMR (101 MHz, $CDCl_3$) δ 213.5, 158.7, 131.1, 127.4, 113.8, 79.5, 55.3, 42.5, 40.4, 38.7, 28.1, 22.9.

HRMS m/z (APCI): calc. for $C_{14}H_{17}O_3^-$ $[M–H]^-$: 233.1183, found: 233.1188.

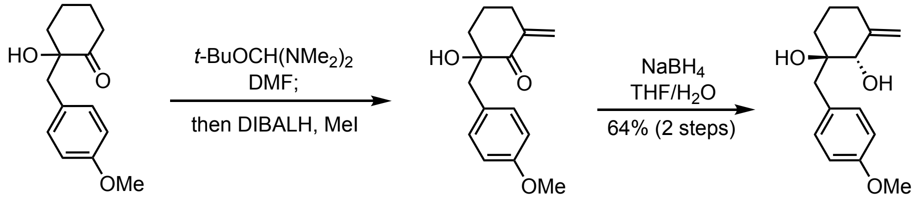

S9 **S10** **S11**

Compound **S9** (2.0 g, 8.55 mmol, 1.0 equiv.) was dissolved in DMF (30 mL). *t*-BuOCH(NMe_2)$_2$ (8.9 mL, 42.8 mmol, 5.0 equiv.) was then added to the solution. The mixture was stirred and heated at 100 °C for 1 h.[3] After TLC showed full consumption of **S9**, the reaction mixture was concentrated under reduced pressure to give the crude enamine, which was used for next step without purification.

The above crude residue was dissolved in CH_2Cl_2 (50 mL) and DIBALH (1.0 M in hexanes, 19.7 mL, 19.7 mmol, 2.3 equiv.) was added at −78 °C. The mixture was warmed up to room temperature and stirred for 30 min before MeI (1.23 mLg, 19.7 mmol, 2.3 equiv.) was added. The stirring was continued for 1 h at the same temperature. The reaction was quenched with a saturated aqueous sodium potassium tartarate (100 mL) at −78 °C. The mixture was warmed up to room temperature and stirred until the solution became clear. The layers were separeted, and the aqueous layer was extracted with DCM (30 mL × 2). The combined organic extracts were washed with brine (50 mL), dried over anhydrous Na_2SO_4, filtered and concentrated under reduced pressure. The crude product **S10** was used without further purification.

The crude product **S10** was dissolved in THF/H_2O (50 mL/5 mL). $NaBH_4$ (162 mg, 4.28 mmol, 0.5 equiv.) was then added to the solution at 0 °C. The stirring was continued for 30 min at the same temperature. After TLC showed full consumption of **S10**, the reaction mixture was quenched with saturated aqueous Na_2CO_3 (20 mL). The layers were separeted, and the aqueous layer was extracted with DCM (30 mL × 2). The combined organic extracts were washed with brine (50 mL), dried over anhydrous Na_2SO_4, filtered and concentrated under reduced pressure. The crude product was purified by flash chromatography (25~33% ethyl acetate in hexane) to yield **S11** (1.36 g, 64%) as a yellow oil.

R_f= 0.5 (hexane/ethyl acetate = 2/1).

IR (film) λ_{max}: 3428, 2934, 1654, 1610, 1511, 1300, 1244, 1177, 1107, 1032, 903, 825, 754 cm^{-1}.

^{1}H NMR (400 MHz, $CDCl_3$) δ 7.17 (d, *J* = 8.6 Hz, 2H), 6.85 (d, *J* = 8.6 Hz, 2H), 5.00 (s, 1H), 4.92 (t, *J* = 1.6 Hz, 1H), 4.00 – 3.95 (m, 1H), 3.79 (s, 3H), 2.89 (d, *J* = 14.1 Hz, 1H), 2.69 (d, *J* = 14.1 Hz, 1H), 2.41

(dt, J = 13.4, 5.6 Hz, 1H), 2.13 (dd, J = 9.0, 4.7 Hz, 2H), 1.83 – 1.72 (m, 2H), 1.71 – 1.51 (m, 2H), 1.45 – 1.34 (m, 1H).

^{13}C NMR (101 MHz, $CDCl_3$) δ 158.4, 147.3, 131.7, 128.9, 113.9, 109.3, 78.6, 75.5, 55.4, 37.9, 33.8, 32.2, 23.0.

HRMS m/z (APCI): calc. for $C_{15}H_{19}O_2^+$ $[M+H–H_2O]^+$: 231.1380, found: 231.1379.

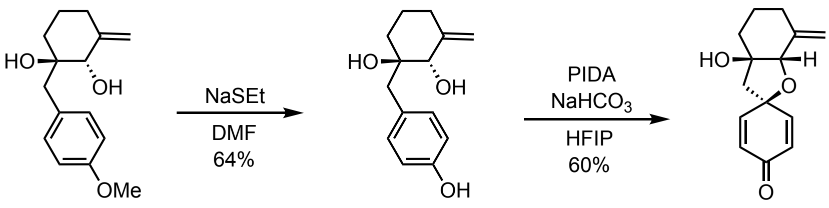

S11 **23** **24**

Compound **S11** (500 mg, 2.0 mmol, 1.0 equiv.) was dissolved in DMF (6.0 mL). Na_2SEt (672 mg, 8.0 mmol, 4.0 equiv.) was then added. The mixture was heated to 130 °C and stirred for 3 h. After TLC showed full consumption of **S11**, the reaction mixture was quenched with saturated aqueous NH_4Cl (20 mL). The layers were separeted, and the aqueous layer was extracted with Et_2O (20 mL × 2). The combined organic extracts were washed with brine (50 mL), dried over anhydrous Na_2SO_4, filtered and concentrated under reduced pressure. The crude product was purified by flash chromatography (5~10% ethyl acetate in hexane) to yield **23** (302 mg, 64%) as a yellow oil.

Compound **24** (50 mg, 0.212 mmol, 1.0 equiv.) was dissolved in HFIP (2.0 mL). $NaHCO_3$ (21.4 mg, 0.424 mmol, 2.0 equiv.) and PIDA (81.8 mg, 0.254 mmol, 1.2 equiv.) were then added at 0 °C. The mixture was stirred for 20 min, then quenched with saturated aqueous Na_2SO_3 (10 mL) and extracted with ethyl acetate (10 mL × 3). The combined organic layers were dried over anhydrous Na_2SO_4, filtered and concentrated *in vacuo.* The crude residue was purified by flash-column chromatography on silica gel (10~24% ethyl acetate in hexane) to afford **24** (30.0 mg, 60%) as a colorless oil.

R_f= 0.6 (hexane/ethyl acetate = 4/1).

IR (film) λ_{max}: 2994, 2254, 1732, 1446, 1372, 1273, 1097, 1044, 915, 847, 730, 648, 607 cm^{-1}.

^{1}H NMR (800 MHz, $CDCl_3$) δ 7.39 (dd, J = 10.2, 1.4 Hz, 1H), 6.93 (d, J = 10.1 Hz, 1H), 6.14 – 6.08 (m, 2H), 5.24 (d, J = 2.5 Hz, 1H), 5.04 (s, 1H), 4.39 (s, 1H), 2.40 (d, J = 14.3 Hz, 1H), 2.27 (d, J = 13.5 Hz, 1H), 2.20 – 2.15 (m, 1H), 2.10 – 2.04 (m, 1H), 1.94 (d, J = 13.5 Hz, 1H), 1.83 – 1.74 (m, 3H), 1.46 – 1.38 (m, 1H).

^{13}C NMR (201 MHz, $CDCl_3$) δ 186.0, 151.4, 146.2, 127.5, 126.2, 112.1, 87.7, 82.1, 77.8, 77.4, 45.6, 36.0, 32.6, 23.3.
HRMS m/z (APCI): calc. for $C_{14}H_{17}IO_3^+$ $[M+H]^+$: 233.1172, found: 233.1169.

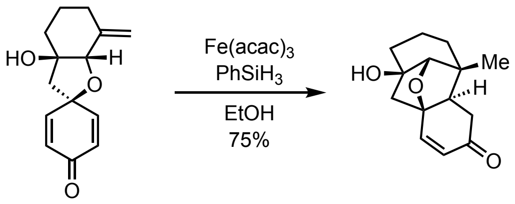

24 **25**

To a stirred solution of **24** (20 mg, 0.085 mmol, 1.0 equiv.) in EtOH (4 mL) at 78 °C were sequentially added $Fe(acac)_3$ (30.0 mg, 0.085 mmol, 1.0 equiv.) and $PhSiH_3$ (11.5 µL, 0.094 mmol, 1.1 equiv.). The resulting mixture was stirred for 1 h at the same temperature. After TLC showed full consumption of **24**, the mixture was directly concentrated *in vacuo*, and the crude residue was purified by flash-column chromatography on silica gel (15~35% ethyl acetate in hexane) to afford **25** (14.8 mg, 75%, CCDC 2450485) as a white solid.

R_f= 0.75 (hexane/ethyl acetate = 10/1).
m.p.= 181–184 °C.
IR (film) λ_{max}: 2983, 1734, 1446, 1372, 1237, 1096, 1044, 914, 847, 730, 648, 607 cm^{-1}.
^{1}H NMR (400 MHz, $CDCl_3$) δ 6.76 (d, J = 10.0 Hz, 1H), 6.13 (d, J = 10.0 Hz, 1H), 3.58 (s, 1H), 2.50 (dd, J = 14.9, 13.5 Hz, 1H), 2.24 (dd, J = 14.9, 5.5 Hz, 1H), 2.01 – 1.91 (m, 3H), 1.82 (s, 1H), 1.74 – 1.63 (m, 3H), 1.62 – 1.54 (m, 2H), 1.38 – 1.27 (m, 1H), 1.07 (s, 3H).
^{13}C NMR (101 MHz, $CDCl_3$) δ 200.7, 143.6, 132.7, 93.6, 81.5, 79.7, 53.0, 44.7, 43.7, 37.3, 33.9, 32.3, 24.1, 18.5.
HRMS m/z (APCI): calc. for $C_{14}H_{19}O_3^+$ $[M+H]^+$: 235.1329, found: 235.1327.

C. Total Synthesis of Neocucurbol C

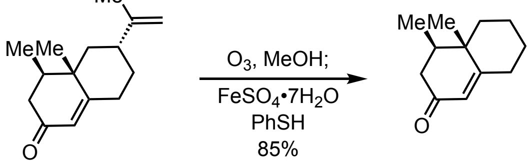

(+)-Nootkatone (**13**) **26**

A round-bottom flask equipped with a magnetic stirrer bar was charged with (+)-nootkatone (20 g, 91.6 mmol, 1.0 equiv.) and MeOH (500 mL) with Sudan Red III (5.0 mg) as a indicator. The flask was then placed in a dry ice/acetone bath and cooled to −78 ˚C while open to the air. Ozone was bubbled through the solution until complete consumption of the starting material had occurred (as indicated the red color turns light yellow). The solution was then sparged with argon for 15 min to expel excess ozone. Benzenethiol (14.0 mL, 137 mmol, 1.5 equiv.) was then added, followed by ferrous sulfate heptahydrate (30.6 g, 110 mmol, 1.2 equiv.). The mixture was stirred at −78 ˚C for 30 min before the cooling bath was removed. After warming to room temperature, the reaction mixture was transferred to a separatory funnel and water (300 mL) was added. The MeOH/water mixture was extracted with dichloromethane (400 mL × 3). The combined organic fractions were washed with brine, dried over Na_2SO_4, and concentrated under reduced pressure. The crude product was subjected to flash chromatography (10~25% ethyl acetate in hexane) to yield enone **26** (13.9 g, 85%) as a colorless oil.[5]

R*f* = 0.5 (hexane/ethyl acetate = 10/1).

$[\alpha]_D^{21}$ = +191.0 (*c* = 0.1 in MeOH).

IR (film) λ_{max}: 2930, 2860, 1665, 1615, 1451, 1288, 1183, 980, 858, 662 cm^{-1}.

^{1}H NMR (400 MHz, $CDCl_3$) δ 3.62 (s, 1H), 2.51 – 2.31 (m, 1H), 2.28 – 2.12 (m, 3H), 2.07 – 1.80 (m, 3H), 1.73 – 1.51 (m, 2H), 1.46 – 1.30 (m, 1H), 1.15 (td, *J* = 13.1, 4.7 Hz, 1H), 1.05 (s, 3H), 0.92 (d, *J* = 6.8 Hz, 3H).

^{13}C NMR (101 MHz, $CDCl_3$) δ 199.8, 171.6, 124.7, 42.3, 40.4, 39.2, 38.8, 33.2, 26.9, 22.0, 16.3, 15.0.

HRMS m/z (APCI): calc. for $C_{12}H_{19}O^+$ $[M+H]^+$: 179.1430, found: 179.1429.

Note: According to the above procedure, more than 30 g of **26** was obtained through multiple pots.

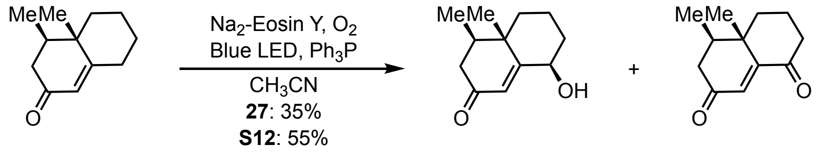

26 **27** **S12**

A 20 mL scintillation vial with a stir bar was charged with enone **26** (100 mg, 0.56 mmol, 1.0 equiv.), Na_2-Eosin Y (11.6 mg, 16.8 μmol, 0.03 equiv.), and anhydrous acetonitrile (10.0 mL). The mixture was bubbled with oxygen for 15 min. The reaction was stirred under an oxygen balloon (1 atm) while exposed to a 456 nm LED lamp (Kessil PR160-456 nm) at room temperature for 16 h. Then, PPh_3 (191 mg, 0.73 mmol, 1.3 equiv.) was added. The reaction was stirred for an additional 4 h at room temperature. Following reaction

completion, the solvent was evaporated in vacuo. The crude product was purified by column chromatography (25~50% ethyl acetate in hexane) to afford alcohol **27** as a colorless oil (38.0 mg, 35 %) and diketone **S12** as a colorless oil (59.1 mg, 55 %).

Data for **27**:

R_f = 0.6 (hexane/ethyl acetate = 1/1).

$[\alpha]_D^{21}$ = +49.0 (c = 0.2 in MeOH).

IR (film) λ_{max}: 3396, 2935, 1657, 1451, 1287, 1059, 870, 730 cm^{-1}.

^{1}H NMR (400 MHz, $CDCl_3$) δ 5.83 (s, 1H), 4.36 (s, 1H), 2.39 – 2.24 (m, 2H), 2.13 – 1.88 (m, 5H), 1.57 (t, J = 17.2 Hz, 2H), 1.27 (s, 3H), 1.22 – 1.03 (m, 1H), 0.93 (d, J = 6.8 Hz, 3H).

^{13}C NMR (101 MHz, $CDCl_3$) δ 200.9, 169.3, 127.3, 72.9, 42.6, 41.2, 38.6, 38.3, 33.0, 17.7, 16.3, 14.7.

HRMS m/z (APCI): calc. for $C_{12}H_{19}O_2^+$ $[M+H]^+$: 195.1380, found: 195.1377.

Data for **S12**:

R_f = 0.8 (hexane/ethyl acetate = 1/1).

$[\alpha]_D^{21}$ = +69.0 (c = 0.1 in MeOH).

IR (film) λ_{max}: 2961, 2358, 1680, 1452, 1413, 1260, 1238, 1126, 941, 842, 668 cm^{-1}.

^{1}H NMR (400 MHz, $CDCl_3$) δ 6.26 (s, 1H), 2.76 – 2.65 (m, 1H), 2.49 – 2.31 (m, 3H), 2.27 – 2.19 (m, 1H), 2.13 – 1.94 (m, 3H), 1.62 – 1.57 (m, 1H), 1.07 (s, 3H), 1.03 (d, J = 6.6 Hz, 3H).

^{13}C NMR (101 MHz, $CDCl_3$) δ 202.5, 199.8, 161.2, 126.5, 42.3, 41.2, 40.9, 40.5, 35.9, 19.6, 17.2, 15.4.

HRMS m/z (APCI): calc. for $C_{12}H_{17}O_2^+$ $[M+H]^+$: 193.1223, found: 193.1223.

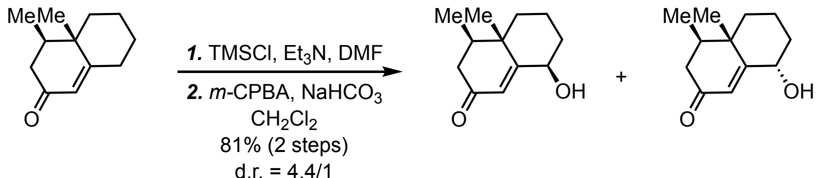

26 **27** (major) **S13** (minor)

Trimethylsilyl chloride (42.6 mL, 337 mmol, 3.0 equiv.) was added dropwise to a vigorously stirred solution of **26** (20 g, 112.3 mmol, 1.0 equiv.) and Et_3N (93 mL, 673.8 mmol, 6.0 equiv.) in DMF (600 mL) at room temperature. The resulting mixture was stirred at 130 ˚C for 16 h. The reaction mixture was cooled to room temperature, followed by the addition of a saturated $NaHCO_3$ aqueous solution (300 mL) and water (200 mL). The layers were separated and the aqueous layer was extracted with hexane (400 mL × 3). The combined organic layers were washed with brine (500 mL), dried over K_2CO_3, filtered and concentrated

under reduced pressure to afford crude silylenolether as a yellow oil that was used in the next step without further purification.

m-CPBA (30.2 g, 134.8 mmol, 1.2 equiv.) and $NaHCO_3$ (18.9 g, 224.6 mmol, 2.0 equiv.) was added to a vigorously stirred solution of the above crude silylenolether in CH_2Cl_2 (400 mL) and H_2O (50 mL) at −10 ˚C. After 10 min, a saturated Na_2SO_3 aqueous solution (300 mL) was added. The layers were separated and the aqueous layer was extracted with CH_2Cl_2 (300 mL × 3). The combined organic layers were washed with brine (500 mL), dried over Na_2SO_4, filtered and concentrated under reduced pressure. The crude product was purified by flash chromatography (25~50% ethyl acetate in hexane) to yield **27** (14.4 g, 66%) as a colorless oil along with its epimer **S13** (3.3 g, 15%). **Note: *m*-CPBA is explosive and should be used with caution!**

Data for **27**:

R_f = 0.6 (hexane/ethyl acetate = 1/1).

$[\alpha]_D^{21}$ = +49.0 (*c* = 0.2 in MeOH).

IR (film) λ_{max}: 3396, 2935, 1657, 1451, 1287, 1059, 870, 730 cm^{-1}.

^{1}H NMR (400 MHz, $CDCl_3$) δ 5.83 (s, 1H), 4.36 (s, 1H), 2.39 – 2.24 (m, 2H), 2.13 – 1.88 (m, 5H), 1.57 (t, *J* = 17.2 Hz, 2H), 1.27 (s, 3H), 1.22 – 1.03 (m, 1H), 0.93 (d, *J* = 6.8 Hz, 3H).

^{13}C NMR (101 MHz, $CDCl_3$) δ 200.9, 169.3, 127.3, 72.9, 42.6, 41.2, 38.6, 38.3, 33.0, 17.7, 16.3, 14.7.

HRMS m/z (APCI): calc. for $C_{12}H_{19}O_2^+$ $[M+H]^+$: 195.1380, found: 195.1377.

Data for **S13**:

R_f = 0.5 (hexane/ethyl acetate = 1/1).

$[\alpha]_D^{21}$ = +41.9 (*c* = 0.3 in MeOH).

IR (film) λ_{max}: 3385, 2962, 2868, 1640, 1605, 1457, 1379, 1087, 865, 715 cm^{-1}.

^{1}H NMR (400 MHz, $CDCl_3$) δ 6.23 (s, 1H), 4.36 (s, 1H), 2.39 – 2.14 (m, 4H), 2.09 – 1.88 (m, 2H), 1.82 – 1.60 (m, 2H), 1.42 (d, *J* = 12.3 Hz, 1H), 1.27 – 1.13 (m, 1H), 1.08 (s, 3H), 0.96 (d, *J* = 6.8 Hz, 3H).

^{13}C NMR (101 MHz, $CDCl_3$) δ 200.0, 172.3, 120.8, 69.3, 42.0, 40.8, 40.1, 38.6, 36.4, 20.1, 17.1, 15.3.

HRMS m/z (APCI): calc. for $C_{12}H_{19}O_2^+$ $[M+H]^+$: 195.1380, found: 195.1377.

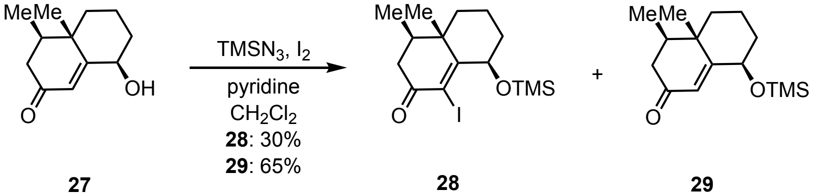

27 28 29

Compound **27** (11.3 g, 58.3 mmol, 1.0 equiv.) was dissolved in DCM (200 mL). Trimethylsilyl azide (7.6 mL, 58.3 mmol, 1.0 equiv) was added to the solution at room temperature. The reaction was stirred for 2 h. Then, pyridine (9.4 mL, 116.5 mmol, 2.0 equiv.) and I_2 (22 g, 87.4 mmol, 1.5 equiv.) were added. The mixture was stirred for another 12 h. The excess I_2 was quenched with saturated aqueous Na_2SO_3 (200 mL) at 0 °C. The layers were separeted, and the aqueous layer was extracted with DCM (200 mL × 2). The combined organic extracts were washed with saturated aqueous $CuSO_4$ (300 mL), brine (300 mL), dried over anhydrous Na_2SO_4, filtered and concentrated under reduced pressure. The crude product was purified by flash chromatography (5~10% ethyl acetate in hexane) to yield **28** (6.9 g, 30%) as a yellow oil, and **29** (10.1 g, 65%) as a yellow oil. **Note: Trimethylsilyl azide should be used with caution on large scale as azides are oftern explosive.**

Data for **28**:

R_f= 0.75 (hexane/ethyl acetate = 10/1).

$[\alpha]_D^{21}$ = –10.0 (*c* = 0.1 in MeOH).

IR (film) λ_{max}: 2939, 1683, 1450, 1248, 1081, 1021, 930, 837, 749, 713, 640 cm^{-1}.

^{1}H NMR (400 MHz, $CDCl_3$) δ 5.18 – 5.12 (m, 1H), 2.54 (s, 1H), 2.52 (d, *J* = 3.0 Hz, 1H), 2.15 – 1.91 (m, 2H), 1.89 – 1.77 (m, 2H), 1.58 – 1.41 (m, 2H), 1.25 (s, 3H), 1.16 (dd, *J* = 13.5, 3.9 Hz, 1H), 0.92 (d, *J* = 6.8 Hz, 3H), 0.15 (s, 9H).

^{13}C NMR (101 MHz, $CDCl_3$) δ 193.3, 171.2, 110.9, 79.1, 43.8, 41.4, 40.6, 39.1, 34.7, 17.3, 16.1, 14.5, 0.8.

HRMS m/z (APCI): calc. for $C_{15}H_{26}IO_2Si^+$ $[M+H]^+$: 393.0741, found: 393.0737.

Data for **29**:

R_f= 0.70 (hexane/ethyl acetate = 10/1).

$[\alpha]_D^{21}$ = +9.0 (*c* = 0.3 in MeOH).

IR (film) λ_{max}: 2937, 1672, 1450, 1248, 1107, 1062, 866, 836, 748, 698 cm^{-1}.

^{1}H NMR (400 MHz, $CDCl_3$) δ 5.76 (s, 1H), 4.28 (t, *J* = 2.9 Hz, 1H), 2.32 (d, *J* = 14.1 Hz, 1H), 2.26 – 2.16 (m, 1H), 2.15 – 1.79 (m, 4H), 1.56 – 1.43 (m, 2H), 1.24 (s, 3H), 1.13 (d, *J* = 3.6 Hz, 1H), 0.92 (d, *J* = 6.9 Hz, 3H), 0.08 (s, 9H).

^{13}C NMR (101 MHz, CDCl$_3$) δ 201.0, 169.2, 126.6, 73.3, 42.6, 41.5, 39.0, 38.8, 34.8, 17.4, 16.4, 14.7, 0.3.
HRMS m/z (APCI): calc. for $C_{15}H_{27}O_2Si^+$ $[M+H]^+$: 267.1775, found:267.1771.

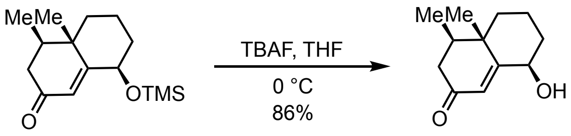

29 **27**

To a solution of **29** (10.1 g, 37.9 mmol, 1.0 equiv.) in THF (250 mL) was added TBAF (1.0 M in THF, 37.9 mL, 37.9 mmol, 1.0 equiv.) at 0 °C. The mixture was stirred for 0.5 h at same temperature. After TLC showed full consumption of **29**, the reaction mixture was quenched with saturated aqueous NH_4Cl (200 mL). The layers were separated and the aqueous layer was extracted with EtOAc (150 mL × 3). The combined organic layers were washed with brine (200 mL), dried over Na_2SO_4, filtered and concentrated under reduced pressure. The crude product was purified by flash chromatography (25~50% ethyl acetate in hexane) to yield **27** (6.4 g, 86%) as a colorless oil.

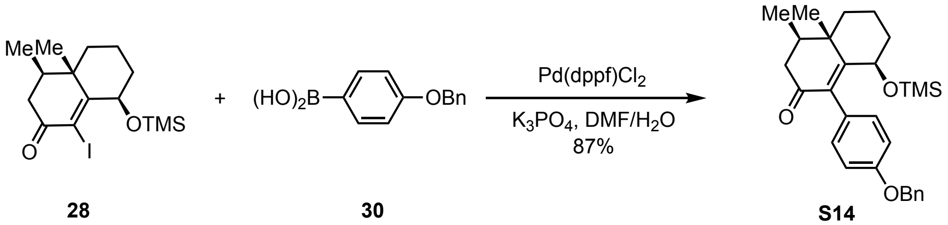

28 **30** **S14**

To a stirred solution of **28** (2.80 g, 7.2 mmol, 1.0 equiv.) in DMF/H_2O (50 mL/5 mL) was added 4-benzyloxybenzeneboronic acid **30** (2.0 g, 8.65 mmol, 1.2 equiv.) and $PdCl_2$(dppf) (531 mg, 0.72 mmol, 0.1 equiv.), K_3PO_4 (3.82 g, 18.0 mmol, 2.5 equiv.) under argon atmosphere. The reaction mixture was heated to 60 °C and stirred for 4 h. After TLC showed full consumption of **28**, the reaction was quenched with saturated aqueous $NaHCO_3$ (50 mL) and extracted with ethyl acetate (50 mL × 3). The combined organic layers were dried over anhydrous Na_2SO_4, filtered and concentrated *in vacuo*. The crude residue was purified by flash-column chromatography on silica gel (5~20% ethyl acetate in hexane) to afford **S14** (2.81 g, 87%) as a yellow oil.

R_f= 0.45 (hexane/ethyl acetate = 10/1).
$[\alpha]_D^{21}$ = +16.0 (*c* = 0.1 in MeOH).
IR (film) λ_{max}: 2935, 1672, 1507, 1452, 1246, 1175, 1080, 908, 835, 734, 647 cm^{-1}.

^{1}H NMR (400 MHz, CDCl$_3$) δ 7.55 – 7.31 (m, 5H), 7.10 – 6.75 (m, 4H), 5.08 (s, 2H), 4.57 (s, 1H), 2.51 (dd, J = 17.0, 14.4 Hz, 1H), 2.34 (dd, J = 17.0, 3.8 Hz, 1H), 2.13 – 1.94 (m, 3H), 1.83 – 1.72 (m, 1H), 1.52 – 1.37 (m, 2H), 1.35 (s, 3H), 1.24 (td, J = 13.4, 3.7 Hz, 1H), 0.96 (d, J = 6.8 Hz, 3H), -0.13 (s, 9H).

^{13}C NMR (101 MHz, CDCl$_3$) δ 199.8, 163.4, 158.1, 137.2, 136.7, 131.5, 128.7, 128.1, 128.0, 127.6, 114.4, 70.1, 68.6, 42.6, 40.7, 39.6, 39.5, 34.7, 17.5, 16.0, 14.9, 0.4.

HRMS m/z (APCI): calc. for $C_{28}H_{37}O_3Si^+$ [M+H]$^+$: 449.2506, found: 449.2497.

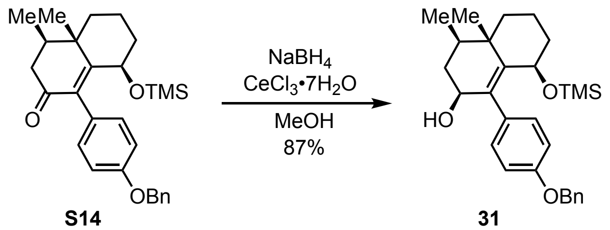

S14 **31**

To a solution of **S14** (7.50 g, 16.8 mmol, 1.0 equiv.) in MeOH (200 mL) was added $CeCl_3 \cdot 7H_2O$ (7.5 g, 20.1 mmol, 1.2 equiv.) at 0 °C. After stirring at the same temperature for 10 min, $NaBH_4$ (950 mg, 25.1 mmol, 1.5 equiv.) was added at 0 °C. The resulting reaction mixture was stirred for 1.5 h before it was quenched with saturated aqueous $NaHCO_3$ (100 mL) and extracted with CH_2Cl_2 (100 mL × 3). The combined organic layers were dried over anhydrous Na_2SO_4, filtered and concentrated *in vacuo.* The crude residue was purified by flash-column chromatography on silica gel (10~30% ethyl acetate in hexane) to afford **31** (6.5 g, 87%) as a colorless oil.

R_f= 0.55 (hexane/ethyl acetate = 4/1).

$[\alpha]_D^{21}$ = –13.0 (c = 0.1 in MeOH).

IR (film) λ_{max}: 3031, 2928, 2863, 1605, 1506, 1380, 1247, 1062, 1025, 836, 738, 696 cm^{-1}.

^{1}H NMR (400 MHz, CDCl$_3$) δ 7.48 – 7.29 (m, 5H), 7.23 (s, 1H), 6.97 (d, J = 8.9 Hz, 3H), 5.07 (s, 2H), 4.38 (ddd, J = 9.6, 6.5, 2.8 Hz, 1H), 4.24 – 4.18 (m, 1H), 1.91 – 1.83 (m, 3H), 1.72 – 1.56 (m, 3H), 1.37 (d, J = 3.2 Hz, 2H), 1.26 (d, J = 4.3 Hz, 1H), 1.24 (s, 3H), 1.08 (dd, J = 13.6, 4.3 Hz, 1H), 0.90 (d, J = 6.2 Hz, 3H), -0.03 (d, J = 0.7 Hz, 9H).

^{13}C NMR (101 MHz, CDCl$_3$) δ 157.9, 142.2, 137.4, 137.1, 132.0, 131.5, 129.1, 128.7, 128.1, 127.7, 115.3, 114.5, 70.6, 70.2, 69.6, 40.0, 39.5, 38.3, 35.6, 35.2, 19.5, 16.3, 15.5, 0.9.

HRMS m/z (APCI): calc. for $C_{28}H_{38}O_3Si^+$ [M]$^+$: 450.2585, found: 450.2586.

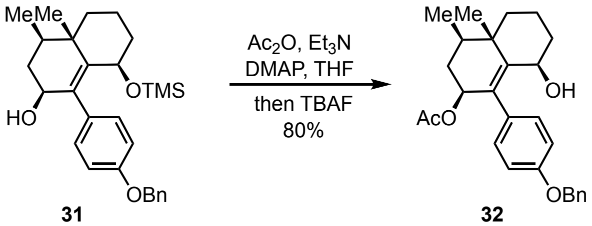

31 **32**

To a solution of **31** (6.25 g, 13.9 mmol, 1.0 equiv.) in THF (80 mL) was added Et_3N (9.63 mL, 69.4 mmol, 5.0 equiv.), DMAP (339 mg, 2.78 mmol, 0.2 equiv.), and Ac_2O (2.58 mL, 27.8 mmol, 2.0 equiv.) at room temperature. The solution was heated to 60 °C and stirred for 1 h. After TLC showed full consumption of **31**, TBAF (1.0 M in THF, 69.4 mL, 69.4 mmol, 5.0 equiv.) was added to the reaction mixture at 60 °C. The resulting reaction mixture was stirred for 2.5 h before quenched with saturated aqueous NH_4Cl (80 mL) and extracted with EtOAc (80 mL × 3). The combined organic layers were dried over anhydrous Na_2SO_4, filtered and concentrated *in vacuo.* The crude residue was purified by flash-column chromatography on silica gel (10~30% ethyl acetate in hexane) to afford **32** (4.63 g, 80%) as a colorless oil.

R_f= 0.3 (hexane/ethyl acetate = 4/1).

$[\alpha]_D^{22}$ = –91.6 (*c* = 0.1 in MeOH).

IR (film) λ_{max}: 3545, 2927, 2865, 1731, 1713, 1505, 1371, 1234, 1177, 1028, 994, 746 cm^{-1}.

^{1}H NMR (400 MHz, $CDCl_3$) δ 7.47 – 7.32 (m, 5H), 7.11 (s, 1H), 6.97 – 6.88 (m, 3H), 5.66 (dd, *J* = 9.5, 6.9 Hz, 1H), 5.04 (s, 2H), 4.25 (q, *J* = 2.9 Hz, 1H), 2.11 – 1.84 (m, 3H), 1.82 – 1.72 (m, 1H), 1.71 – 1.69 (m, 2H), 1.69 (s, 3H), 1.50 – 1.30 (m, 2H), 1.29 (s, 3H), 1.23 – 1.18 (m, 1H), 1.10 (td, *J* = 13.6, 3.8 Hz, 1H), 0.91 (d, *J* = 6.0 Hz, 3H).

^{13}C NMR (101 MHz, $CDCl_3$) δ 170.6, 157.7, 144.4, 137.1, 136.7, 131.6, 130.5, 129.1, 128.7, 128.1, 127.7, 114.6, 72.6, 70.1, 68.1, 39.3, 39.1, 37.5, 33.6, 32.8, 21.0, 19.8, 16.3, 15.3.

HRMS m/z (APCI): calc. for $C_{27}H_{32}O_6^-$ $[M+O_2]^-$: 452.2204, found: 452.2215.

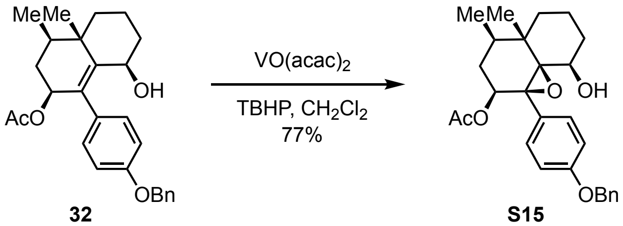

32 **S15**

To a solution of **32** (4.45 g, 10.4 mmol, 1.0 equiv.) in DCM (150 mL) was added $VO(acac)_2$ (2.71 g, 10.4 mmol, 1.0 equiv.) and TBHP (5.5 M in decane, 18.9 mL, 104 mmol, 10 equiv.) at 0 °C. The solution was

stirred for 5 h at room temperature. The mixture was then quenched with saturated aqueous Na_2SO_3 (100 mL) and extracted with ethyl acetate (100 mL × 3). The combined organic layers were dried over anhydrous Na_2SO_4, filtered and concentrated *in vacuo.* The crude residue was purified by flash-column chromatography on silica gel (20~33% ethyl acetate in hexane) to afford **S15** (3.43 g, 77%) as a yellow oil. **Note: TBHP should be used in caution on large scale.**

R_f= 0.3 (hexane/ethyl acetate = 4/1).
$[\alpha]_D^{22}$ = –30.2 (c = 0.1 in MeOH).
IR (film) λ_{max}: 3513, 2934, 2869, 1734, 1513, 1238, 1072, 1026, 925, 830, 698 cm^{-1}.
^{1}H NMR (400 MHz, $CDCl_3$) δ 7.45 – 7.20 (m, 6H), 7.12 (dd, J = 8.4, 1.6 Hz, 1H), 6.96 – 6.87 (m, 2H), 5.42 (dd, J = 10.5, 5.3 Hz, 1H), 5.03 (s, 2H), 3.09 – 3.03 (m, 1H), 2.03 (dt, J = 6.3, 4.5 Hz, 2H), 1.85 – 1.77(m, 2H), 1.79 (s, 3H), 1.64 (dd, J = 12.8, 10.5 Hz, 1H), 1.56 – 1.44 (m, 2H), 1.42 – 1.35 (m, 1H), 1.27 – 1.25 (m, 3H), 1.19 (s, 3H), 0.86 (d, J = 6.7 Hz, 3H).
^{13}C NMR (101 MHz, $CDCl_3$) δ 170.3, 158.4, 136.9, 128.9, 128.8, 128.7, 128.2, 127.7, 126.5, 114.9, 114.5, 74.3, 73.5, 73.4, 70.2, 69.3, 40.6, 39.8, 35.8, 31.2, 30.8, 20.9, 16.0, 15.5, 15.4.
HRMS m/z (APCI): calc. for $C_{27}H_{32}O_5^+$ $[M]^+$: 436.2244, found: 436.2239.

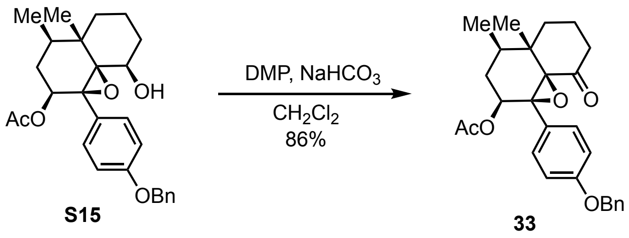

To a solution of **S15** (3.04 g, 6.98 mmol, 1.0 equiv.) in CH_2Cl_2 (100 mL) was added DMP (4.45 g, 10.5 mmol, 1.5 equiv.) and $NaHCO_3$ (1.48 g, 17.5 mmol, 2.5 equiv.) at 0 °C. The solution was stirred for 2 h at room temperature. After TLC showed full consumption of **S15**, the mixture was quenched with saturated aqueous Na_2SO_3 (100 mL) and extracted with ethyl acetate (100 mL × 3). The combined organic layers were dried over anhydrous Na_2SO_4, filtered and concentrated *in vacuo.* The crude residue was purified by flash-column chromatography on silica gel (10~30% ethyl acetate in hexane) to afford **33** (2.6 g, 86%) as a yellow oil.

R_f= 0.55 (hexane/ethyl acetate = 4/1).
$[\alpha]_D^{22}$ = +13.6 (c = 0.1 in MeOH).

IR (film) λ_{max}: 2938, 2871, 1722, 1611, 1512, 1236, 1027, 830, 741, 697 cm^{-1}.

^{1}H NMR (400 MHz, $CDCl_3$) δ 7.46 – 7.29 (m, 5H), 7.24 (d, *J* = 8.4 Hz, 2H), 6.91 – 6.84 (m, 2H), 5.63 (dd, *J* = 10.5, 5.3 Hz, 1H), 4.99 (d, *J* = 1.6 Hz, 2H), 2.35 – 2.23 (m, 1H), 2.03 – 1.84 (m, 4H), 1.81 (s, 3H), 1.77 – 1.59 (m, 4H), 0.98 (s, 3H), 0.93 (d, *J* = 6.8 Hz, 3H).

^{13}C NMR (101 MHz, $CDCl_3$) δ 204.4, 170.5, 158.7, 136.9, 128.7, 128.2, 127.8, 126.1, 114.5, 76.6, 72.9, 70.1, 69.4, 41.5, 38.9, 37.7, 37.2, 30.4, 20.9, 19.3, 17.5, 15.9.

HRMS m/z (APCI): calc. for $C_{27}H_{30}O_5^+$ $[M]^+$: 434.2088, found: 434.2084.

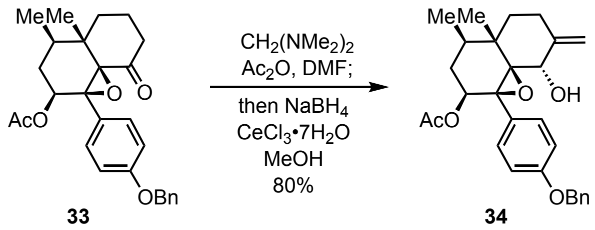

To a stirred solution of ketone **33** (2.5 g, 5.75 mmol, 1.0 equiv.) in DMF (30 mL) at room temperature were sequentially added $CH_2(NMe_2)_2$ (7.9 mL, 57.5 mmol, 10.0 equiv.) and Ac_2O (4.3 mL, 46.1 mmol, 8.0 equiv.). The resulting mixture was heated to 95 °C and stirred for 12 h. After TLC showed full consumption of **33**, the mixture was directly concentrated *in vacuo*. The residue was dissolved in MeOH (40 mL). $CeCl_3\cdot 7H_2O$ (3.22 g, 8.65 mmol, 1.5 equiv.) was added at 0 °C. After stirring at the same temperature for 10 min, the reaction mixture was added $NaBH_4$ (545 mg, 14.4 mmol, 2.5 equiv.) at 0 °C. The resulting reaction mixture was stirred for 1.5 h before quenched with saturated aqueous $NaHCO_3$ (30 mL) and extracted with CH_2Cl_2 (40 mL × 3). The combined organic layers were dried over anhydrous Na_2SO_4, filtered and concentrated *in vacuo.* The crude residue was purified by flash-column chromatography on silica gel (10~30% ethyl acetate in hexane) to afford **34** (2.05 g, 80%) as a colorless oil.

R_f= 0.5 (hexane/ethyl acetate = 3/1).

$[\alpha]_D^{22}$ = –84.0 (*c* = 0.1 in MeOH).

IR (film) λ_{max}: 3674, 2970, 2900, 2360, 2341, 1735, 1512, 1405, 1026, 920, 734, 696 cm^{-1}.

^{1}H NMR (600 MHz, $CDCl_3$) δ 7.46 – 7.30 (m, 7H), 6.96 (d, *J* = 8.2 Hz, 2H), 5.62 – 5.56 (m, 1H), 5.04 (s, 2H), 4.98 – 4.89 (m, 2H), 3.65 (d, *J* = 3.7 Hz, 1H), 2.53 (ddd, *J* = 17.0, 7.2, 3.1 Hz, 1H), 2.45 – 2.36 (m, 1H), 2.01 (ddd, *J* = 13.6, 10.6, 7.3 Hz, 1H), 1.80 (s, 3H), 1.80 – 1.72 (m, 1H), 1.63 – 1.57 (m, 3H), 1.31 (d, *J* = 3.7 Hz, 1H), 0.95 (s, 3H), 0.86 (d, *J* = 6.1 Hz, 3H).

^{13}C NMR (151 MHz, CDCl$_3$) δ 170.5, 158.6, 144.1, 136.8, 129.1, 128.7, 128.5, 128.2, 127.8, 114.8, 113.0, 76.3, 74.3, 74.0, 70.2, 68.0, 37.8, 36.4, 30.5, 30.1, 24.9, 20.9, 15.6, 15.0.

HRMS m/z (APCI): calc. for $C_{28}H_{32}O_5^+$ $[M]^+$: 448.2244, found: 448.2237.

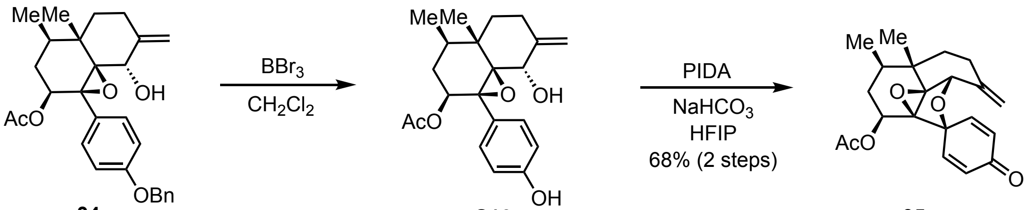

34 **S16** **35**

To a solution of **34** (600 mg, 1.34 mmol, 1.0 equiv.) in DCM (40 mL) was added BBr_3 (1.0 M in CH_2Cl_2, 2.7 mL, 2.68 mmol, 2.0 equiv.) at −78 °C. After stirring for 10 min at same temperature, the reaction mixture was quenched with saturated aqueous $NaHCO_3$ (20 mL) and extracted with ethyl acetate (40 mL × 3). The combined organic layers were dried over anhydrous Na_2SO_4, filtered and concentrated *in vacuo.* The crude residue was filtered over silica gel, washing with EtOAc/hexanes (1/2) to afford crude **S16** as a yellow oil, which is unstable and was used in the next step directly.

The crude residue obtained above was dissolved in HFIP (10 mL), then $NaHCO_3$ (225 mg, 2.68 mmol, 2.0 equiv.) and PIDA (475 mg, 1.47 mmol, 1.1 equiv.) were added at 0 °C. The mixture was stirred for 20 min, then quenched with saturated aqueous Na_2SO_3 (10 mL) and extracted with ethyl acetate (20 mL × 3). The combined organic layers were dried over anhydrous Na_2SO_4, filtered and concentrated *in vacuo.* The crude residue was purified by flash-column chromatography on silica gel (10~24% ethyl acetate in hexane) to afford **35** (324 mg, 68% 2 steps) as a colorless oil.

R_f = 0.75 (hexane/ethyl acetate = 3/1).

$[\alpha]_D^{22}$ = +24.0 (*c* = 0.1 in MeOH).

IR (film) λ_{max}: 3674, 2969, 2899, 2360, 1741, 1691, 1393, 1231, 1065, 1048, 915 cm^{-1}.

^{1}H NMR (600 MHz, CDCl$_3$) δ 6.94 (dd, *J* = 10.3, 2.9 Hz, 1H), 6.74 (dd, *J* = 10.4, 2.9 Hz, 1H), 6.25 (ddd, *J* = 10.4, 8.7, 1.9 Hz, 2H), 5.20 (dd, *J* = 10.2, 5.4 Hz, 1H), 5.07 (t, *J* = 2.1 Hz, 1H), 4.97 (dt, *J* = 2.8, 1.6 Hz, 1H), 4.68 (t, *J* = 2.5 Hz, 1H), 2.36 – 2.29 (m, 1H), 2.20 – 2.05 (m, 2H), 1.88 (s, 3H), 1.61 – 1.53 (m, 2H), 1.53 – 1.35 (m, 3H), 1.10 (s, 3H), 0.86 (d, *J* = 6.9 Hz, 3H).

^{13}C NMR (151 MHz, CDCl$_3$) δ 185.0, 170.0, 146.8, 146.4, 145.1, 130.2, 129.3, 108.2, 79.6, 77.68, 77.65, 74.0, 69.3, 41.8, 41.0, 35.4, 31.5, 28.5, 20.5, 16.7, 15.2.

HRMS m/z (APCI): calc. for $C_{21}H_{25}O_5^+$ $[M+H]^+$: 357.1696, found: 357.1695.

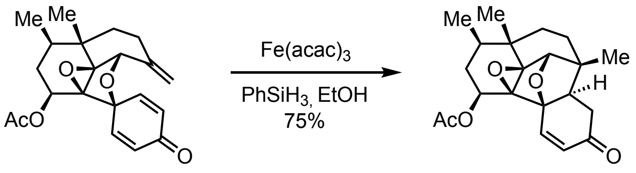

To a stirred solution of **35** (300 mg, 0.843 mmol, 1.0 equiv.) in EtOH (17 mL) at 78 °C were sequentially added Fe(acac)$_3$ (297 mg, 0.843 mmol, 1.0 equiv.) and PhSiH$_3$ (114 μL, 0.927 mmol, 1.1 equiv.) under argon atmosphere. The resulting mixture was stirred for 1 h at the same temperature. After TLC showed full consumption of **35**, the mixture was directly concentrated *in vacuo*, and the crude residue was purified by flash-column chromatography on silica gel (10~25% ethyl acetate in hexane) to afford **36** (226 mg, 75%, CCDC 2424690) as a white solid.

R$_f$= 0.5 (hexane/ethyl acetate = 3/1).

m.p.= 168–170 °C.

[α]$_D^{22}$ = +137.4 (*c* = 0.1 in MeOH).

IR (film) λ_{max}: 3674, 2970, 2899, 2361, 1739, 1648, 1405, 1233, 1066, 1047, 936, 903, 668 cm^{-1}.

^{1}H NMR (400 MHz, CDCl$_3$) δ 6.83 (d, *J* = 10.0 Hz, 1H), 6.14 (d, *J* = 10.1 Hz, 1H), 5.33 (dd, *J* = 9.2, 6.2 Hz, 1H), 4.03 (s, 1H), 2.49 – 2.27 (m, 3H), 2.04 (s, 3H), 1.81 (dd, *J* = 15.1, 9.1 Hz, 1H), 1.68 (dd, *J* = 6.8, 4.5 Hz, 1H), 1.59 – 1.56 (m, 3H), 1.54 – 1.41 (m, 2H), 1.11 (s, 3H), 1.04 (s, 3H), 0.83 (d, *J* = 6.8 Hz, 3H).

^{13}C NMR (101 MHz, CDCl$_3$) δ 199.0, 170.5, 138.8, 133.4, 82.8, 81.1, 69.3, 68.0, 63.9, 52.2, 46.6, 37.7, 34.5, 34.0, 32.8, 32.6, 31.4, 23.8, 21.0, 17.7, 15.5.

HRMS m/z (APCI): calc. for $C_{21}H_{27}O_5^+$ $[M+H]^+$: 359.1853, found: 359.1853.

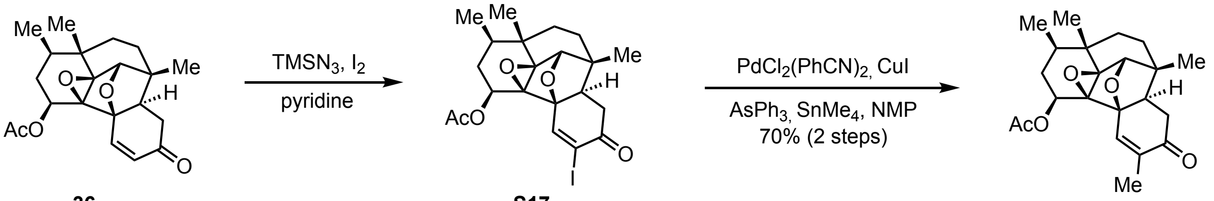

Compound **36** (282 mg, 0.788 mmol, 1.0 equiv.) was dissolved in DCM (10 mL). Trimethylsilyl azide (TMSN$_3$) (207 μL, 1.58 mmol, 2.0 equiv.) was then added to the solution at room temperature. The mixture was stirred for 2 h before pyridine (253 μL, 3.15 mmol, 4.0 equiv.) and I$_2$ (0.40 g, 1.58 mmol, 2.0 equiv.)

were added. The mixture was stirred for another 12 h. The excess I_2 was quenched with saturated aqueous Na_2SO_3 (10 mL) at 0 °C. The layers were separeted, and the aqueous layer was extracted with DCM (10 mL × 2). The combined organic extracts were washed with saturated aqueous $CuSO_4$ (20 mL), brine (20 mL), dried over anhydrous Na_2SO_4, filtered and concentrated under reduced pressure. The crude residue was filtered over silica gel, washing with EtOAc/hexanes (1/10) to afford crude **S17** as a yellow oil, which was used in the next step directly. **Note: Trimethylsilyl azide should be used with caution on large scale as azides are oftern explosive.**

The crude residue obtained above was dissolved in NMP (10 mL) was added $AsPh_3$ (48.4 mg, 0.156 mmol, 0.2 equiv.), CuI (30 mg, 0.156 mmol, 0.2 equiv.), $SnMe_4$ (1.10 mL, 7.88 mmol, 10.0 equiv.), and $PdCl_2(PhCN)_2$ (30.3 mg, 0.079 mmol, 0.1 equiv.) under argon atmosphere. The reaction mixture was heated to 80 °C and stirred for 4 h. After TLC showed full consumption of **S17**, the reaction was quenched with saturated aqueous $NaHCO_3$ (20 mL) and extracted with ethyl acetate (20 mL × 3). The combined organic layers were dried over anhydrous Na_2SO_4, filtered and concentrated *in vacuo* and the crude residue was purified by flash-column chromatography on silica gel (10~20% ethyl acetate in hexane) to afford **37** (207 g, 70% 2 steps) as a yellow oil.

R_f= 0.55 (hexane/ethyl acetate = 3/1).
$[\alpha]_D^{22}$ = +87.0 (c = 0.1 in MeOH).
IR (film) λ_{max}: 3674, 2969, 2938, 2361, 1740, 16831455, 1405, 1240, 1065, 1046, 928 cm^{-1}.
^{1}H NMR (400 MHz, $CDCl_3$) δ 6.56 (d, J = 1.5 Hz, 1H), 5.36 (dd, J = 9.8, 5.6 Hz, 1H), 4.00 (s, 1H), 2.53 – 2.38 (m, 2H), 2.36 – 2.26 (m, 1H), 2.03 (s, 3H), 1.90 – 1.75 (m, 4H), 1.73 – 1.50 (m, 4H), 1.50 – 1.42 (m, 2H), 1.09 (s, 3H), 1.04 (s, 3H), 0.84 (d, J = 6.6 Hz, 3H).
^{13}C NMR (101 MHz, $CDCl_3$) δ 199.2, 170.4, 140.7, 133.7, 82.6, 82.0, 69.2, 68.0, 63.8, 52.4, 46.5, 37.8, 34.6, 34.1, 32.8, 32.6, 31.5, 23.8, 20.9, 17.8, 16.1, 15.5.
HRMS m/z (APCI): calc. for $C_{22}H_{29}O_5^+$ $[M+H]^+$: 373.2009, found: 373.2006.

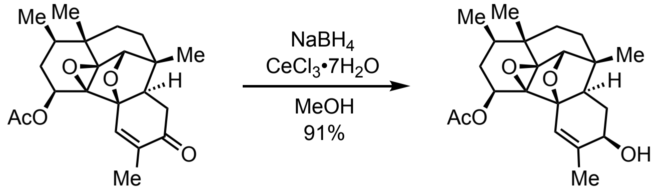

37 **S18**

To a solution of **37** (170 mg, 0.457 mmol, 1.0 equiv.) in MeOH (10 mL) was added $CeCl_3 \cdot 7H_2O$ (255 mg, 0.685 mmol, 1.5 equiv.) at 0 °C. After stirring at the same temperature for 10 min, $NaBH_4$ (34.5 mg, 0.913 mmol, 2.0 equiv.) was added at 0 °C. The resulting reaction mixture was stirred for 1.5 h before it was quenched with saturated aqueous $NaHCO_3$ (10 mL) and extracted with CH_2Cl_2 (15 mL × 3). The combined organic layers were dried over anhydrous Na_2SO_4, filtered and concentrated *in vacuo.* The crude residue was purified by flash-column chromatography on silica gel (10~30% ethyl acetate in hexane) to afford **S18** (155 mg, 91%) as a colorless oil.

R*f*= 0.5 (hexane/ethyl acetate = 1/1).
[α]D²² = +20.8 (*c* = 0.1 in MeOH).
IR (film) λ_{max}: 3674, 3457, 2969, 2361, 1739, 1405, 1373, 1240, 1065, 1050, 922, 668 cm^{-1}.
^{1}H NMR (400 MHz, $CDCl_3$) δ 5.53 – 5.47 (m, 1H), 5.44 (dd, *J* = 10.1, 5.2 Hz, 1H), 4.17 – 4.07 (m, 1H), 3.85 (s, 1H), 2.03 (s, 3H), 1.98 – 1.86 (m, 2H), 1.85 – 1.77 (m, 1H) 1.80 (s, 3H), 1.73 – 1.59 (m, 3H), 1.54 (d, *J* = 15.0 Hz, 1H), 1.48 – 1.38 (m, 4.6 Hz, 4H), 1.07 (s, 3H), 1.00 (s, 3H), 0.82 (d, *J* = 5.9 Hz, 3H).
^{13}C NMR (201 MHz, $CDCl_3$) δ 170.8, 147.3, 117.0, 83.0, 81.5, 70.8, 69.1, 67.7, 63.6, 52.0, 45.9, 34.8, 34.3, 33.0, 32.6, 32.4, 31.6, 23.8, 21.0, 18.9, 17.8, 15.6.
HRMS m/z (APCI): calc. for $C_{22}H_{31}O_5^+$ $[M+H]^+$: 375.2166, found: 375.2167.

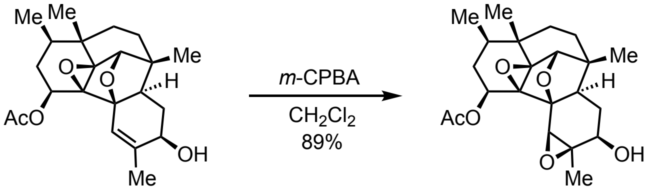

S18 **38**

The **S18** (132 mg, 0.352 mmol, 1.0 equiv.) obtained above was dissolved in CH_2Cl_2 (6 mL). *m*-CPBA (77%, 118 mg, 0.528 mmol, 1.5 equiv.) was then added at 0 °C. The mixture was stirred for 2 h at room temperature. After TLC showed full consumption of **S18** the reaction mixture was quenched with saturated aqueous Na_2SO_3 (10 mL) and extracted with ethyl acetate (10 mL × 3). The combined organic layers were dried over anhydrous Na_2SO_4, filtered and concentrated *in vacuo.* The crude residue was purified by flash-column chromatography on silica gel (30~50% ethyl acetate in hexane) to afford **38** (122 mg, 89%) as a colorless oil. **Note: *m*-CPBA is explosive and should be used with caution!**

R*f*= 0.4 (hexane/ethyl acetate = 1/1).

[α]D[22] = +13.0 (c = 0.1 in MeOH).

IR (film) λ_{max}: 3674, 2970, 2899, 2360, 2087, 1733, 1405, 1241, 1065, 1048, 1027, 879 cm^{-1}.

^{1}H NMR (400 MHz, $CDCl_3$) δ 5.53 (dd, J = 10.1, 5.1 Hz, 1H), 3.88 (q, J = 3.2 Hz, 2H), 3.22 (s, 1H), 2.08 (s, 3H), 1.75 – 1.61 (m, 6H), 1.53 (dt, J = 14.9, 3.6 Hz, 1H), 1.45 (s, 3H), 1.46 – 1.32 (m, 4H), 1.04 (s, 3H), 1.00 (s, 3H), 0.83 (d, J = 6.2 Hz, 3H).

^{13}C NMR (101 MHz, $CDCl_3$) δ 170.8, 83.1, 80.7, 71.6, 69.9, 67.9, 62.6, 60.3, 58.2, 53.0, 46.2, 34.6, 34.2, 32.8, 32.5, 31.4, 28.6, 23.7, 21.1, 18.1, 17.7, 15.5.

HRMS m/z (APCI): calc. for $C_{22}H_{31}O_6^+$ $[M+H]^+$: 391.2115, found: 391.2113.

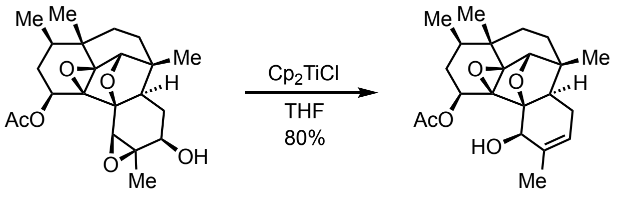

38 **39**

Preparation of Cp_2TiCl: A mixture of Cp_2TiCl_2 (280 mg, 1.13 mmol, 4.0 equiv.) and Zn (148 mg, 2.26 mmol, 8.0 equiv.) in strictly deoxygenated THF (4.0 mL) was stirred at room temperature for 1 h, until the red solution turned green. Carefully draw out the supernatant with a syringe for later use.

To a solution of **38** (110 mg, 0.282 mmol, 1.0 equiv.) in THF (5.0 mL) was added the obove Cp_2TiCl solution slowly at room temperature. The reaction mixture was stirred at the same temperature for about 2 h. After TLC showed full consumption of **38**, the reaction mixture was quenched with saturated aqueous $NaHCO_3$ (5 mL) and extracted with CH_2Cl_2 (5 mL × 3). The combined organic layers were dried over anhydrous Na_2SO_4, filtered and concentrated *in vacuo.* The crude residue was purified by flash-column chromatography on silica gel (10~25% ethyl acetate in hexane) to afford **39** (84.2 mg, 80%) as a colorless oil.

R*f* = 0.75 (hexane/ethyl acetate = 1/1).

[α]D[22] = –34.6 (c = 0.1 in MeOH).

IR (film) λ_{max}: 3674, 3395, 2968, 2032, 2360, 1733, 1454, 1393, 1236, 1044, 732 cm^{-1}.

^{1}H NMR (600 MHz, $CDCl_3$) δ 5.51 – 5.44 (m, 2H), 4.35 (d, J = 6.6 Hz, 1H), 3.87 (s, 1H), 2.17 (d, J = 7.0 Hz, 1H), 2.13 (s, 3H), 2.09 – 1.96 (m, 3H), 1.82 – 1.81 (m, 1H), 1.78 (s, 3H), 1.73 – 1.59 (m, 3H), 1.52 (dd, J = 14.8, 3.6 Hz, 1H), 1.48 – 1.43 (m, 2H), 1.03 (s, 3H), 1.01 (s, 3H), 0.83 (d, J = 6.8 Hz, 3H).

^{13}C NMR (151 MHz, $CDCl_3$) δ 171.2, 135.7, 121.6, 88.4, 80.7, 69.0, 68.6, 68.2, 62.2, 52.2, 48.0, 34.6, 33.7, 32.9, 32.4, 31.5, 24.8, 22.3, 21.3, 19.3, 17.5, 15.4.

HRMS m/z (APCI): calc. for $C_{22}H_{30}NaO_5^+$ $[M+Na]^+$: 397.1696, found: 397.1695.

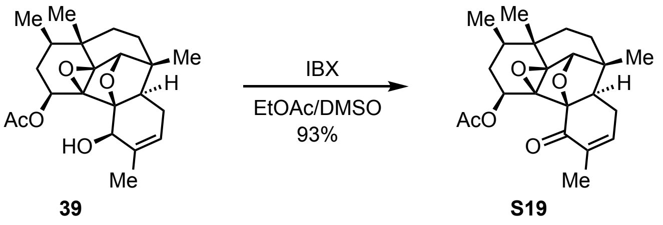

To a solution of **39** (78.1 mg, 0.209 mmol, 1.0 equiv.) in EtOAc (5.0 mL) was added DMSO (0.5 mL) and IBX (234 mg, 0.835 mmol, 4.0 equiv.) at room temperature. The reaction mixture was stirred at 82 °C for about 4 h. After TLC showed full consumption of **39**, the reaction mixture was quenched with saturated aqueous Na_2SO_3 (5.0 mL) and extracted with CH_2Cl_2 (5.0 mL × 3). The combined organic layers were dried over anhydrous Na_2SO_4, filtered and concentrated *in vacuo* and the crude residue was purified by flash-column chromatography on silica gel (15~35% ethyl acetate in hexane) to afford **S19** (72.4 mg, 93%) as a colorless oil.

R_f = 0.5 (hexane/ethyl acetate = 3/1).

$[\alpha]_D^{22}$ = –74.0 (c = 0.1 in MeOH).

IR (film) λ_{max}: 3674, 2958, 2923, 2361, 1725, 1670, 1452, 1371, 1246, 1065, 1046, 934, 739 cm^{-1}.

^{1}H NMR (400 MHz, $CDCl_3$) δ 6.71 (ddd, J = 5.8, 3.2, 1.5 Hz, 1H), 5.42 (dd, J = 10.8, 5.2 Hz, 1H), 3.97 (s, 1H), 2.42 (t, J = 8.7 Hz, 1H), 2.35 – 2.23 (m, 2H), 2.02 (s, 3H), 1.89 – 1.73 (m, 5H), 1.73 – 1.53 (m, 2H), 1.48 – 1.35 (m, 3H), 1.13 (s, 3H), 1.03 (s, 3H), 0.84 (d, J = 6.9 Hz, 3H).

^{13}C NMR (101 MHz, $CDCl_3$) δ 191.7, 171.5, 144.2, 136.7, 85.4, 82.6, 67.7, 67.6, 63.0, 53.6, 46.9, 34.8, 34.1, 32.7, 32.6, 31.5, 24.5, 24.4, 21.0, 17.8, 15.8, 15.5.

HRMS m/z (APCI): calc. for $C_{22}H_{29}O_5^+$ $[M+H]^+$: 373.2009, found: 373.2007.

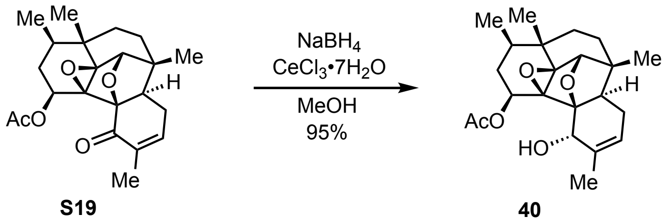

To a solution of **S19** (50 mg, 0.127 mmol, 1.0 equiv.) in MeOH (4.0 mL) was added $CeCl_3 \cdot 7H_2O$ (94.4 mg, 0.253 mmol, 2.0 equiv.) at 0 °C. After stirring at the same temperature for 10 min, $NaBH_4$ (19.1 mg, 0.507 mmol, 4.0 equiv.) was added at 0 °C. The resulting reaction mixture was stirred for 0.5 h before it was quenched with saturated aqueous $NaHCO_3$ (5.0 mL) and extracted with CH_2Cl_2 (5.0 mL × 3). The combined organic layers were dried over anhydrous Na_2SO_4, filtered and concentrated *in vacuo* and the crude residue was purified by flash-column chromatography on silica gel (20~50% ethyl acetate in hexane) to afford **40** (45.0 mg, 95%) as a colorless oil.

R_f = 0.4 (hexane/ethyl acetate = 5/1).
$[\alpha]_D^{22}$ = +3.0 (*c* = 0.1 in MeOH).
IR (film) λ_{max}: 3674, 3474, 2968, 2929, 2361, 1740, 1452, 1404, 1252, 1046, 668 cm^{-1}.
^{1}H NMR (400 MHz, $CDCl_3$) δ 5.64 – 5.58 (m, 1H), 5.36 (dd, *J* = 10.1, 5.4 Hz, 1H), 4.33 – 4.26 (m, 1H), 3.88 (s, 1H), 2.40 (d, *J* = 8.3 Hz, 1H), 2.13 (s, 3H), 2.04 (d, *J* = 7.6 Hz, 1H), 1.80 (dd, *J* = 7.5, 4.7 Hz, 1H), 1.76 (s, 3H), 1.70 – 1.42 (m, 7H), 1.05 (s, 3H), 1.00 (s, 3H), 0.81 (d, *J* = 6.7 Hz, 3H).
^{13}C NMR (101 MHz, $CDCl_3$) δ 170.1, 137.9, 122.7, 93.5, 80.0, 70.6, 69.5, 69.2, 62.1, 54.6, 45.8, 34.1, 33.4, 32.7, 32.4, 31.2, 24.1, 23.0, 21.4, 18.2, 17.5, 15.3.
HRMS m/z (APCI): calc. for $C_{22}H_{31}O_5^+$ $[M+H]^+$: 375.2166, found: 375.2162.

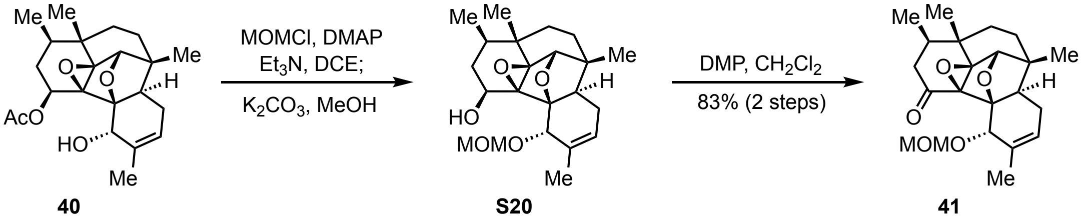

To a solution of **40** (32.6 mg, 0.087 mmol, 1.0 equiv.) in DCE (1.0 mL) was added DMAP (2.15 mg, 0.0175 mmol, 0.2 equiv.), Et_3N (226 μL, 1.75 mmol, 20.0 equiv.) and MOMCl (13.2 μL, 0.174 mmol, 2.0 equiv.). After TLC showed full consumption of **40**, MeOH (2.0 mL) and K_2CO_3 (60 mg, 0.435 mmol, 5.0 equiv.) were added. The resulting reaction mixture was stirred for 4 h at the same temperature before quenched with saturated aqueous $NaHCO_3$ (4 mL) and extracted with CH_2Cl_2 (4 mL × 3). The combined organic layers were dried over anhydrous Na_2SO_4, filtered and concentrated *in vacuo* and the crude residue was purified by flash-column chromatography on silica gel (33~50% ethyl acetate in hexane) to afford crude **S20** as a colorless oil.

The crude **S20** residue obtained above was dissolved in CH_2Cl_2 (4.0 mL). DMP (55.5 mg, 0.131 mmol, 1.5 equiv.) and $NaHCO_3$ (22.0 mg, 0.261 mmol, 3.0 equiv.) were added at 0 °C. The solution was stirred for 2 h at room temperature. After TLC showed full consumption of **S20**, the mixture was quenched with saturated aqueous Na_2SO_3 (5 mL) and extracted with ethyl acetate (5 mL × 3). The combined organic layers were dried over anhydrous Na_2SO_4, filtered and concentrated *in vacuo* and the crude residue was purified by flash-column chromatography on silica gel (10~25% ethyl acetate in hexane) to afford **41** (27.0 mg, 86%) as a yellow oil.

R_f = 0.5 (hexane/ethyl acetate = 4/1).
$[\alpha]_D^{22}$ = –110.4 (*c* = 0.1 in MeOH).
IR (film) λ_{max}: 2968, 2930, 2361, 1700, 1457, 1375, 1150, 1050, 1026, 919, 734 cm^{-1}.
^{1}H NMR (400 MHz, $CDCl_3$) δ 5.70 – 5.63 (m, 1H), 4.91 (dd, *J* = 6.7, 1.1 Hz, 1H), 4.63 (d, *J* = 6.6 Hz, 1H), 4.41 – 4.35 (m, 1H), 3.85 (s, 1H), 3.42 (S, 3H), 2.40 (dd, *J* = 6.9, 4.6 Hz, 2H), 1.94 (s, 3H), 1.93 – 1.90 (m, 2H), 1.76 – 1.61 (m, 4H), 1.37 (d, *J* = 6.1 Hz, 2H), 1.19 (s, 3H), 1.15 (d, *J* = 7.2 Hz, 3H), 1.05 (s, 3H).
^{13}C NMR (101 MHz, $CDCl_3$) δ 205.0, 137.9, 121.3, 96.3, 93.4, 81.1, 73.7, 73.5, 63.1, 56.0, 49.4, 46.2, 43.5, 40.0, 35.1, 34.7, 31.7, 23.8, 23.1, 19.6, 18.5, 18.0.
HRMS m/z (APCI): calc. for $C_{22}H_{30}NaO_5^+$ $[M+Na]^+$: 397.1985, found: 397.1983.

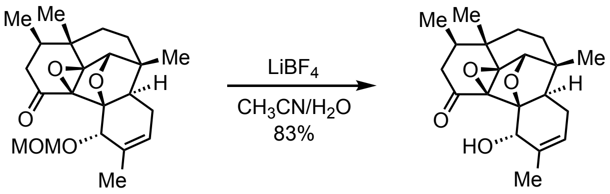

41 **S21**

To a solution of the **41** (14.4 mg, 0.039 mmol, 1.0 equiv.) in CH_3CN (2 mL) were added distilled water (200 μL) and $LiBF_4$ (72.0 mg, 0.77 mmol, 20.0 equiv.) at room temperature. The mixture was stirred for 2.5 h at 82 °C. After TLC showed full consumption of **41**, the reaction was quenched with saturated aqueous $NaHCO_3$ (2.0 mL) and extracted with CH_2Cl_2 (5 mL × 3). The combined organic layers were dried over anhydrous Na_2SO_4, filtered and concentrated *in vacuo*. The residue was purified by flash-column chromatography on silica gel (15%~25% ethyl acetate in hexane) to afford **S21** (11.5 mg, 83%) as a white powder.

R$_f$= 0.5 (hexane/ethyl acetate = 3/1).

[α]$_D^{22}$ = –63.0 (*c* = 0.1 in MeOH).

IR (film) λ_{max}: 3674, 3500, 2969, 2921, 2363, 1689, 1454, 1404, 1250, 1073, 1026, 948, 801, 689 cm^{-1}.

^{1}H NMR (600 MHz, $CDCl_3$) δ 5.64 (d, *J* = 5.2 Hz, 1H), 4.23 (d, *J* = 9.1 Hz, 1H), 3.87 (s, 1H), 3.06 (d, *J* = 9.3 Hz, 1H), 2.49 – 2.45 (m, 2H), 2.05 – 1.92 (m, 2H), 1.92 (s, 3H), 1.81 (t, *J* = 13.0 Hz, 1H), 1.75 – 1.64 (m, 3H), 1.50 – 1.37 (m, 2H), 1.22 (s, 3H), 1.16 (d, *J* = 7.2 Hz, 3H), 1.06 (s, 3H).

^{13}C NMR (151 MHz, $CDCl_3$) δ 208.1, 137.0, 121.8, 92.4, 80.9, 72.9, 69.6, 63.3, 47.2, 47.0, 42.9, 39.4, 34.6, 34.4, 31.9, 24.4, 22.9, 19.4, 19.0, 18.0.

HRMS m/z (APCI): calc. for $C_{20}H_{27}O_4^+$ $[M+H]^+$: 331.1904, found: 331.1903.

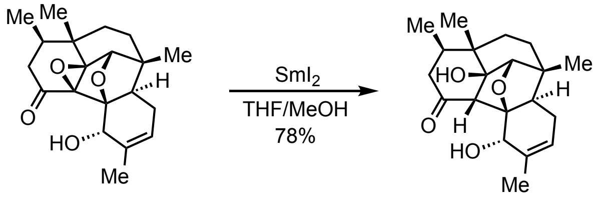

S21 Neocucurbol C (**3**)

To a solution of **S21** (7.0 mg, 0.021 mmol, 1.0 equiv.) in THF/MeOH (2.0 mL/200 uL) was added SmI_2 (0.1 M in THF, 630 μL, 2.10 mmol, 3.0 equiv.) at −78 °C. After stirring for 10 min, the reaction mixture was quenched with saturated aqueous $NaHCO_3$ (5.0 mL) and extracted with CH_2Cl_2 (2.0 mL × 3). The combined organic layers were dried over anhydrous Na_2SO_4, filtered and concentrated *in vacuo*. The crude residue was purified by flash-column chromatography on silica gel (25~50% ethyl acetate in hexane) to afford **3** (5.5 mg, 78%, CCDC 2424691) as a colorless solid.

R$_f$= 0.5 (hexane/ethyl acetate = 1/1).

m.p.= 164–166 °C

[α]$_D^{22}$ = –18.0 (*c* = 0.1 in MeOH).

Natural [α]$_D^{25}$ = +28.0 (*c* = 0.1 in MeOH)[6].

IR (film) λ_{max}: 3428, 2924, 2872, 2853, 1690, 1554, 1458, 1374, 1326, 1246, 1186, 1092, 1029, 991, 957, 938, 804, 775, 734, 701, 607 cm^{-1}.

^{1}H NMR (600 MHz, CD_3OD) δ 5.62 – 5.57 (m, 1H), 4.28 (s, 1H), 3.50 (s, 1H), 2.72 – 2.64 (m, 1H), 2.17 (dd, *J* = 17.4, 3.8 Hz, 1H), 2.00 – 1.93 (m, 1H), 1.88 (dt, *J* = 6.2, 3.4 Hz, 1H), 1.83 (S, 3H), 1.74 (t, *J* = 12.6 Hz, 1H), 1.61 (dd, *J* = 9.6, 6.1 Hz, 1H), 1.55 (td, *J* = 13.9, 4.7 Hz, 1H), 1.43 (m, 1H), 1.35 – 1.32 (m, 4H), 1.28 – 1.20 (m, 1H), 1.17 – 1.15 (m, 1H), 1.14 (s, 3H), 0.97 (S, 3H).

^{13}C NMR (151 MHz, CD_3OD) δ 215.3, 139.9, 122.3, 95.4, 91.5, 86.4, 72.1, 64.8 (m), 46.5, 43.3, 43.2, 42.9, 40.3, 33.8, 30.9, 24.1, 23.6, 21.1, 19.5, 18.8.

HRMS m/z (APCI): calc. for $C_{20}H_{29}O_4^+$ $[M+H]^+$: 333.2060, found: 333.2055.

Note: The optical rotation of neocucurbol C was revised to be $[\alpha]_D^{22}$ = –18.0 (*c* = 0.1 in MeOH), and the reasons are as follows:

1. The specific optical rotation value of our synthetic neocucurbol C had been re-evaluated to be $[\alpha]_D^{22}$ = –18.0 (*c* = 0.1 in MeOH) instead of +28.0 (*c* = 0.1 in MeOH) by different people and different instruments for multiple times.
2. A single crystal of our synthetic neocucurbol C was obtained. Consequently, the structure and absolute configuration of our synthetic neocucurbol C were unambiguously confirmed by the X-ray crystallographic analysis.
3. Although single crystal data with a Flack Parameter of 0.02(11) has been reported in the previous literature, our synthesized single crystal exhibits a Flack Parameter of 0.005(5). Clearly, our data are more reliable due to the smaller Flack Parameter and the lower associated uncertainty.
4. Our starting material (+)-nootkatone was purchased from Sigma-Aldrich, and it was isolated from plants, while (−)-Nootkatone does not exist in nature and is not commercially available. We tested (+)-nootkatone optical rotation, which was consistent with the optical rotation of (+)-Nootkatone in the literature.
5. In addition, we obtained single crystals of compound **36** and determined its absolute configuration (Flack Parameter -0.03(3)). This also provides strong support for our conclusions.
6. The isolated natural sample is not pure (as indicated by the NMR spectra, which show many unidentified impurity peaks). These impurities may cause the differences between two observed optical rotations (see S33).
7. We obtained the ECD spectrum of the synthetic sample, which is consistent with the one reported in isolation literature.

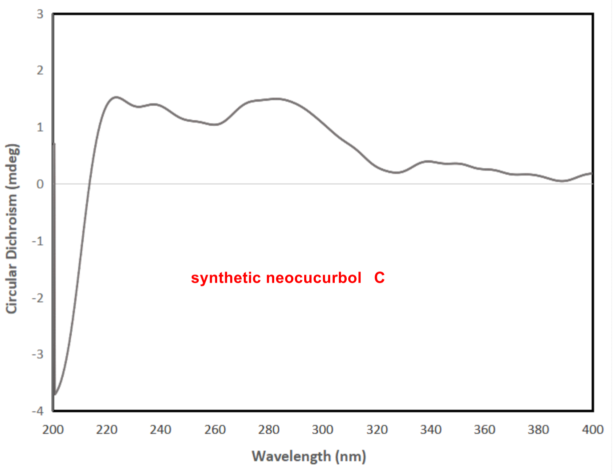

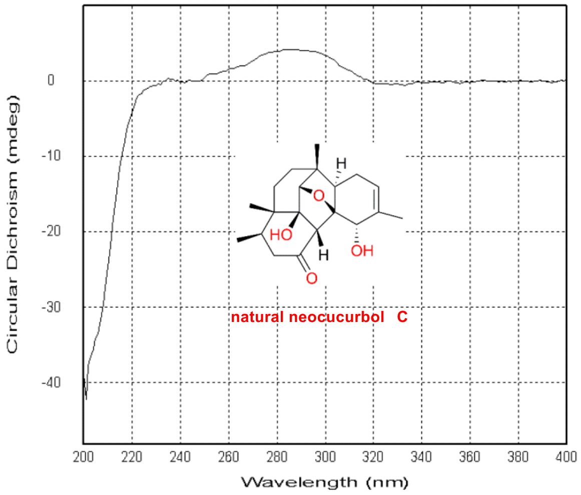

Part 2. NMR Comparison

Table S1. ^{1}H NMR (CD_3OD) Comparison of Natural & Our Synthetic Neocucurbol C.

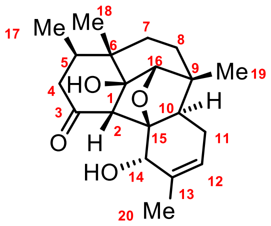

Neocucurbol C (**3**)

**No.**	**Natural, 500 MHz**[6] δ (ppm, *J* in Hz)	**Synthetic, 600 MHz** δ (ppm, *J* in Hz)	**Error** [a] **(Syn. – Nat.)**
2	1.33, m	3.02, s	**b**
4α	2.67, dd(9.5, 17.4)	2.72 – 2.64, m	–
4β	2.16, dd (3.8, 17.4)	2.17, dd (3.8, 17.4)	0.01
5	1.86, m	1.87, dt (6.2, 3.4)	0.01
7α	1.23, m	1.24, m	0.01
7β	1.13, m	1.17 – 1.15, m	**c**
8α	1.42, m	1.43, m	0.01
8β	1.53, m	1.55, m	0.02
10	1.60, m	1.61, dd (9.6, 6.1)	0.01
11α	1.95, m	1.96, m	0.01
11β	1.73, m	1.74, m	0.01
12	5.58, m	5.62 – 5.57, m	–
14	4.27, m	4.28, s	0.01
16	3.49, brs	3.50, brs	0.01
17	1.32, d (7.4)	1.33, (overlap)	0.01
18	1.13, s	1.14, s	0.01
19	0.97, s	0.97, s	0
20	1.82, s	1.83, s	0.01

a. Synthetic spacturm was calibrated the solvent peak at 3.31 ppm, but the isolated literature did not mention the calibration. **b.** As the proton at the α-position of the carbonyl group, it should neither appear at such a high field as 1.33 ppm nor be a multiplet (m), because there are no adjacent protons to couple with it. From the C-H HSQC spectrum in the isolation literature[6], it is clear that they mistakenly assigned the C2 H to 1.33 ppm. **c.** From the HSQC spectrum in the isolated literature, it can be seen that the peak assigned by the authors at 1.13 ppm should belong to impurity peaks.

The C2 proton has relatively strong acidity and is quickly replaced by D atom in CD_3OD, causing the deuterium atom to split C2, resulting in a multiplet for C2 carbon.

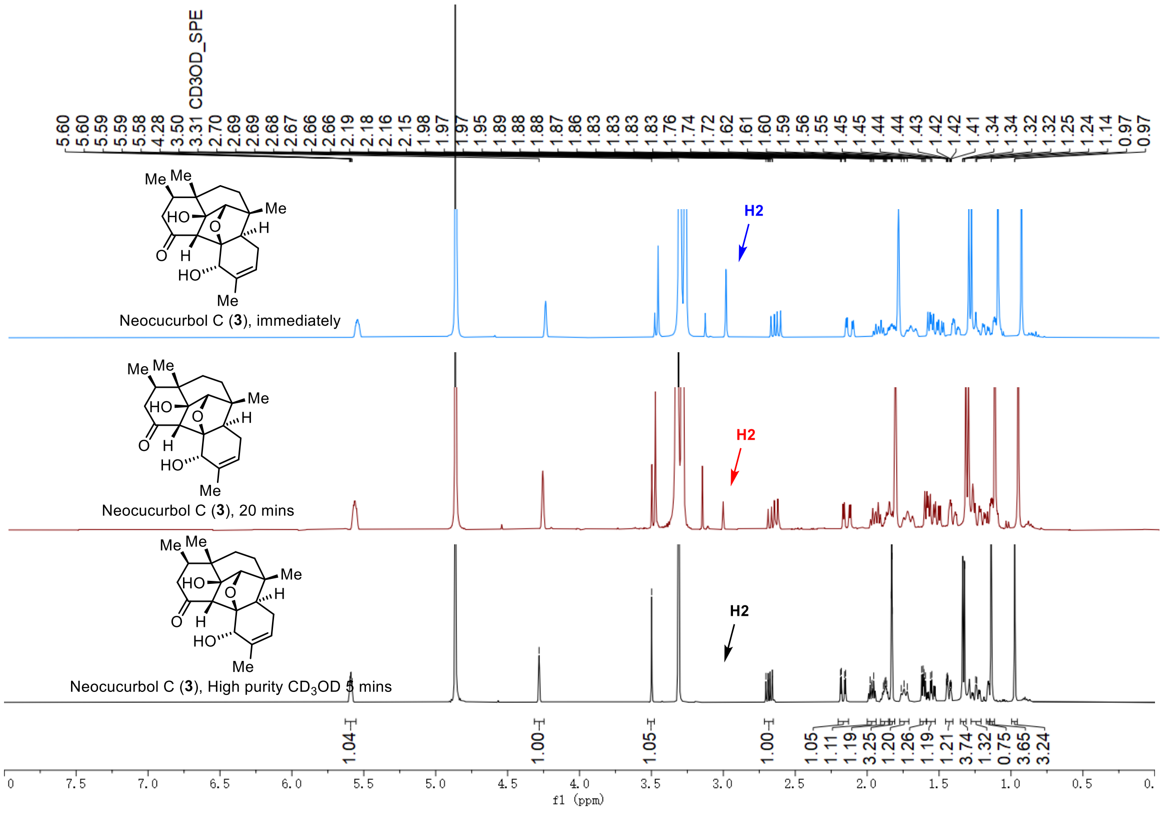

Table S2. ^{13}C NMR (CD_3OD) Comparison of Natural & Our Synthetic Neocucurbol C.

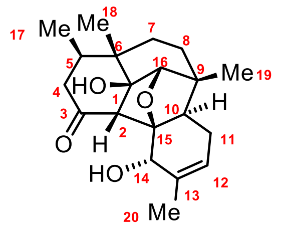

Neocucurbol C (**3**)

No.	Natural, 125 MHz[6] δ (ppm)	Synthetic, 151 MHz δ (ppm)	Error (Syn. – Nat.)
1	86.4	86.4	0
2	54.9[a]	64.8 (m)[b]	–
3	215.3	215.3	0
4	46.5	46.5	0
5	43.2	43.2	0
6	40.3	40.3	0
7	33.8	33.8	0
8	30.9	30.9	0
9	43.3	43.2	–0.1
10	42.9	42.9	0
11	23.6	23.6	0
12	122.3	122.3	0
13	139.9	139.9	0
14	72.1	72.1	0
15	95.4	95.4	0
16	91.5	91.5	0
17	21.1	21.1	0
18	18.8	18.8	0
19	24.1	24.1	0
20	19.5	19.5	0

a. The peak at 54.9 ppm here should be assigned to be dichloromethane.
b. The C2 proton has replaced by D atom in CD_3OD, causing the deuterium atom to split C2, resulting in a multiplet for C2.

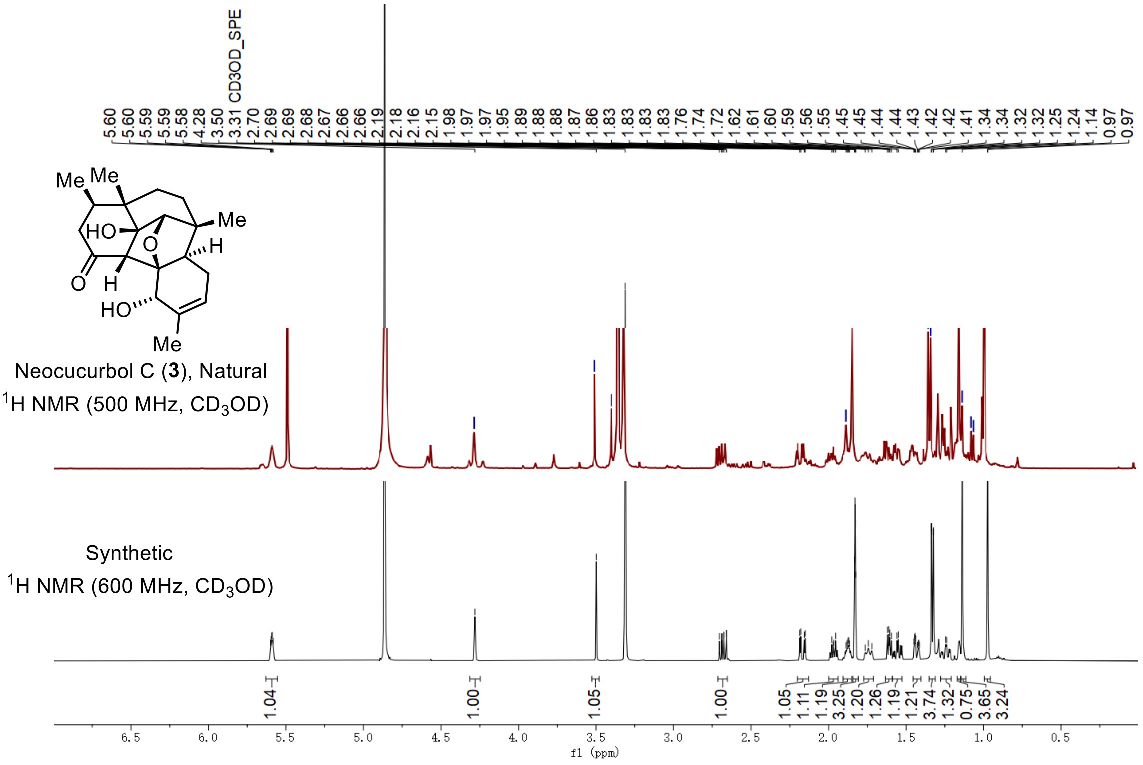
Neocucurbol C (3), Natural
^{1}H NMR (500 MHz, CD_3OD)
Synthetic
^{1}H NMR (600 MHz, CD_3OD)
f1 (ppm)

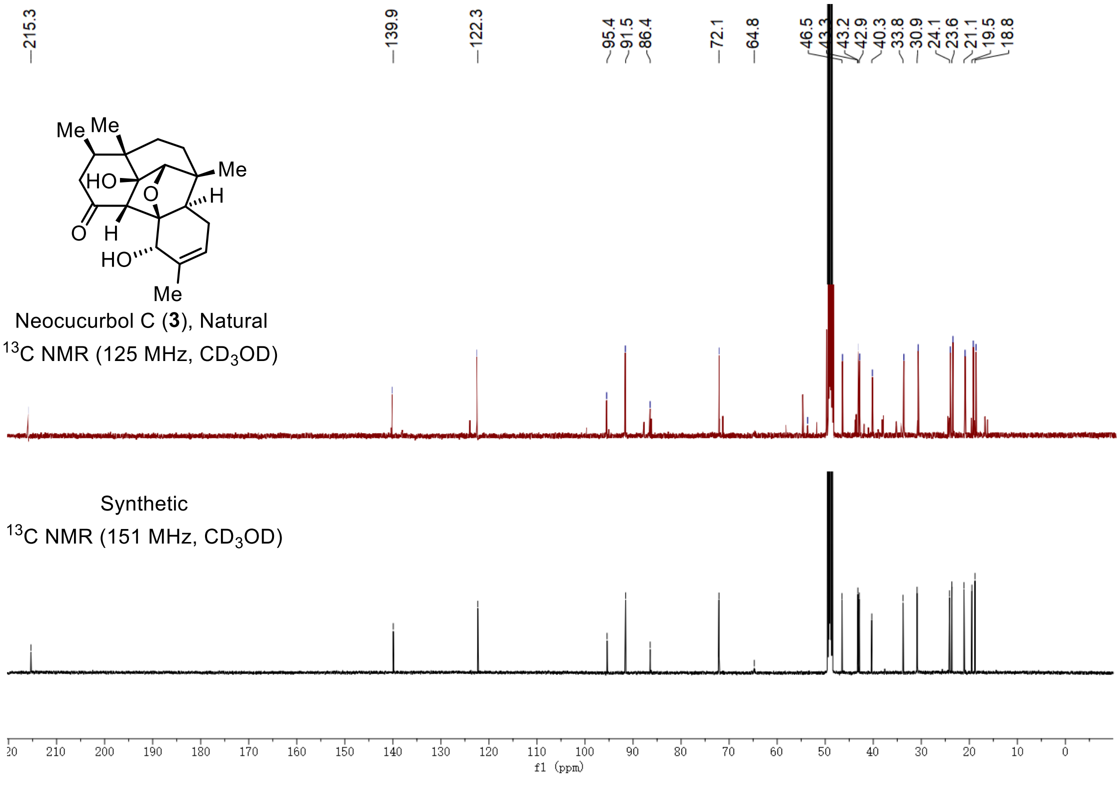
Neocucurbol C (3), Natural
^{13}C NMR (125 MHz, CD_3OD)
Synthetic
^{13}C NMR (151 MHz, CD_3OD)
f1 (ppm)

Part 3. X-ray Crystal Data

Crystal Data and Experimental for **25**

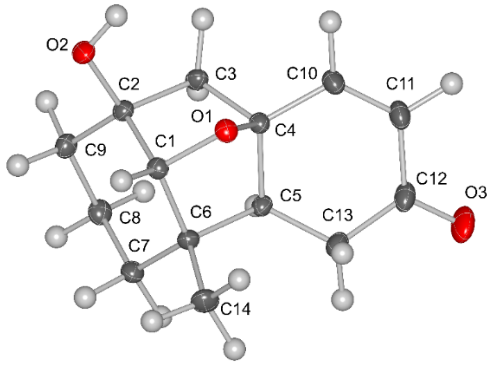

Experimental. Single colorless prism-shaped crystals of **25** recrystallized from MeOH by slow evaporation. A suitable crystal with dimensions 0.24 × 0.18 × 0.12 mm^3 was selected and mounted on a loop with paratone on a XtaLAB AFC11 (RCD3): quarter-chi single diffractometer. The crystal was kept at a steady *T* = 173(1) K during data collection. The structure was solved with the ShelXT 2018/2 (Sheldrick, 2018) solution program using iterative methods and by using Olex2 1.5-alpha (Dolomanov et al., 2009) as the graphical interface. The model was refined with olex2.refine 1.5-alpha (Bourhis et al., 2015) using full matrix least squares minimisation on $\boldsymbol{F}^2$.

Crystal Data. $C_{14}H_{18}O_3$, M_r = 234.297, monoclinic, $P2_1/n$ (No. 14), a = 8.8469(2) Å, b = 26.9816(5) Å, c = 19.5593(4) Å, *b* = 94.690(2)°, *a* = *g* = 90°, *V* = 4653.24(17) Å^3, *T* = 173.03(16) K, *Z* = 16, *Z'* = 4, *m*(Cu K_a) = 0.750, 160281 reflections measured, 8924 unique (R_{int} = 0.0417) which were used in all calculations. The final wR_2 was 0.0299 (all data) and R_1 was 0.0165 (I≥2 *s*(I)).

**Compound**	**25**
CCDC2450485	
Formula	$C_{14}H_{18}O_3$
$D_{calc.}$/ g cm^{-3}	1.338
m/mm^{-1}	0.750
Formula Weight	234.297
Color	colorless
Shape	prism-shaped
Size/mm^3	0.24×0.18×0.12
T/K	173.03(16)
Crystal System	monoclinic
Space Group	$P2_1/n$
a/Å	8.8469(2)
b/Å	26.9816(5)
c/Å	19.5593(4)
a/°	90
b/°	94.690(2)
g/°	90
V/Å^3	4653.24(17)
Z	16
Z'	4
Wavelength/Å	1.54184
Radiation type	Cu K_a
Q_{min}/°	2.80
Q_{max}/°	72.49
Measured Refl's.	160281
Indep't Refl's	8924
Refl's I≥2 *s*(I)	7965
R_{int}	0.0417
Parameters	1262
Restraints	1380
Largest Peak	0.1247
Deepest Hole	-0.1410
GooF	1.1056
wR_2 (all data)	0.0299
wR_2	0.0289
R_1 (all data)	0.0213
R_1	0.0165

Crystal Data and Experimental for **36**

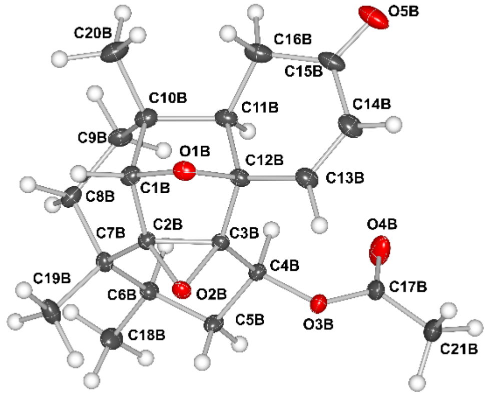

Experimental. Single colorless prism-shaped crystals of **36** recrystallized from MeOH by slow evaporation. A suitable crystal with dimensions 0.29 × 0.16 × 0.05 mm^3 was selected and mounted on a loop with IVH oil on a XtaLAB Synergy, Dualflex, HyPix diffractometer. The crystal was kept at a steady T = 100.00(10) K during data collection. The structure was solved with the ShelXT (Sheldrick, 2015) solution program using dual methods and by using Olex2 1.5-alpha (Dolomanov et al., 2009) as the graphical interface. The model was refined with olex2.refine 1.5-alpha (Bourhis et al., 2015) using full matrix least squares minimisation on $\boldsymbol{F}^2$.

Crystal Data. $C_{21}H_{26}O_5$, M_r = 358.437, monoclinic, $P2_1$ (No. 4), a = 8.2453(1) Å, b = 17.1153(2) Å, c = 12.6423(1) Å, b = 90.066(1)°, a = g = 90°, V = 1784.09(3) $Å^3$, T = 100.00(10) K, Z = 4, Z' = 2, m(Cu K_a) = 0.770, 41997 reflections measured, 7331 unique (R_{int} = 0.0335) which were used in all calculations. The final wR_2 was 0.0840 (all data) and R_1 was 0.0337 (I≥2 s(I)).

**Compound**	**36**
CCDC 2424690	
Formula	$C_{21}H_{26}O_5$
$D_{calc.}$/ g cm^{-3}	1.334
m/mm^{-1}	0.770
Formula Weight	358.437
Color	colorless
Shape	plate-shaped
Size/mm^3	0.29×0.16×0.05
T/K	100.00(10)
Crystal System	monoclinic
Flack Parameter	-0.03(3)
Hooft Parameter	-0.03(3)
Space Group	$P2_1$
a/Å	8.2453(1)
b/Å	17.1153(2)
c/Å	12.6423(1)
a/°	90
b/°	90.066(1)
g/°	90
V/$Å^3$	1784.09(3)
Z	4
Z'	2
Wavelength/Å	1.54184
Radiation type	Cu K_a
Q_{min}/°	4.35
Q_{max}/°	77.16
Measured Refl's.	41997
Indep't Refl's	7331
Refl's I≥2 s(I)	7253
R_{int}	0.0335
Parameters	822
Restraints	1040
Largest Peak	0.4753
Deepest Hole	-0.2352
GooF	1.0849
wR_2 (all data)	0.0840
wR_2	0.0839
R_1 (all data)	0.0339
R_1	0.0337

Crystal Data and Experimental for **3**

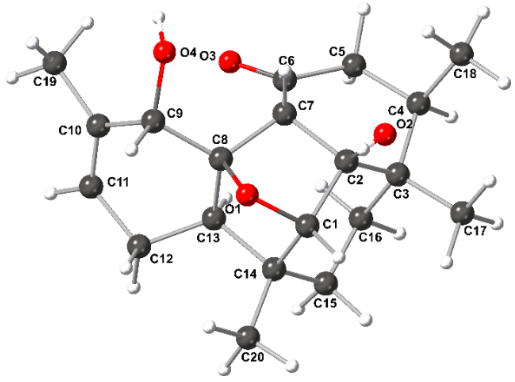

Experimental. Single colorless prism-shaped crystals of **3** recrystallized from EtOAc by slow evaporation. A suitable crystal with dimensions 0.22 × 0.16 × 0.07 mm^3 was selected and mounted on a loop with paratone on a XtaLAB Synergy-S diffractometer. The crystal was kept at a steady T = 100.01(10) K during data collection. The structure was solved with the ShelXT (Sheldrick, 2015) solution program and by using Olex2 1.5-alpha (Dolomanov et al., 2009) as the graphical interface. The model was refined with olex2.refine 1.5-alpha (Bourhis et al., 2015) using full matrix least squares minimisation on $\boldsymbol{F^2}$.

Crystal Data. $C_{182}H_{268}Cl_4O_{47}$, M_r = 3349.939, triclinic, P1 (No. 1), a = 13.9265(2) Å, b = 13.9510(2) Å, c = 24.6322(4) Å, a = 95.807(1)°, b = 91.746(1)°, g = 116.223(1)°, V = 4256.00(12) Å^3, T = 100.01(10) K, Z = 1, Z' = 1, m(Cu K_a) = 1.313, 74541 reflections measured, 25809 unique (R_{int} = 0.0439) which were used in all calculations. The final wR_2 was 0.1637 (all data) and R_1 was 0.0592 (I≥2 s(I)).

**Compound**	**3**
CCDC 2424691	
Formula	$C_{182}H_{268}Cl_4O_{47}$
$D_{calc.}$/ g cm^{-3}	1.307
m/mm^{-1}	1.313
Formula Weight	3349.939
Color	colorless
Shape	plate-shaped
Size/mm^3	0.22×0.16×0.07
T/K	100.01(10)
Crystal System	triclinic
Flack Parameter	0.005(5)
Hooft Parameter	0.005(5)
Space Group	P1
a/Å	13.9265(2)
b/Å	13.9510(2)
c/Å	24.6322(4)
a/°	95.807(1)
b/°	91.746(1)
g/°	116.223(1)
V/Å^3	4256.00(12)
Z	1
Z'	1
Wavelength/Å	1.54184
Radiation type	Cu K_a
Q_{min}/°	3.55
Q_{max}/°	77.12
Measured Refl's.	74541
Indep't Refl's	25809
Refl's I≥2 s(I)	22621
R_{int}	0.0439
Parameters	1956
Restraints	5473
Largest Peak	0.3412
Deepest Hole	-0.5831
GooF	1.0133
wR_2 (all data)	0.1637
wR_2	0.1594
R_1 (all data)	0.0639
R_1	0.0592

Part 4. Biological evaluations

Cytotoxicity of Neocucurbol C and synthetic intermediates

We evaluated the cytotoxic effects of neocucurbol C and ten synthetic intermediates using high-content imaging with DAPI staining[7] in four carcinoma cell lines: A431 (epidermoid adenocarcinoma), Caco-2 (colorectal carcinoma), HeLa (cervical carcinoma), and MDA-MB-231 (breast adenocarcinoma). Cell viability was assessed after treatment with 50 μM of each compound. Staurosporine was included as a positive control.[8,9] Among the compounds tested, **35** and **36** exhibited the highest cytotoxicity, reducing cell viability to below 5% across all four cell lines (Table S3).

	**A431**	**Caco-2**	**HeLa**	**MDA-MB-231**
**35**	0 ± 0	2 ± 1	0 ± 0	1 ± 1
**36**	1 ± 0	4 ± 2	2 ± 0	3 ± 0
**37**	100 ± 3	52 ± 8	81 ± 5	122 ± 3
**S18**	54 ± 2	97 ± 2	87 ± 3	101 ± 6
**38**	45 ± 19	115 ± 3	67 ± 3	110 ± 9
**39**	90 ± 3	77 ± 11	61 ± 5	110 ± 5
**S19**	77 ± 0	84 ± 5	75 ± 2	105 ± 3
**40**	93 ± 2	105 ± 4	111 ± 3	120 ± 3
**41**	90 ± 0	103 ± 2	99 ± 2	95 ± 4
**S21**	85 ± 8	90 ± 9	118 ± 2	119 ± 4
**3**	86 ± 6	94 ± 3	115 ± 8	98 ± 1

Table S3. Cytotoxicity of neocucurbol C and ten synthetic intermediates. Cells were treated with 0.5% DMSO (vehicle), 1 μM staurosporine (positive control), or 50 μM of each compound for 72 hours. Following treatment, cells were stained with DAPI and imaged. Data are presented as percent viable cells ± SD (n = 3).

Next, EC_{50} values for cytotoxicity were determined for compounds **35** and **36** (Figure S1). Compound **35** showed similar EC_{50} values of approximately 13 μM in A431, HeLa, and MDA-MB-231 cells, and 22 μM in Caco-2 cells. In comparison, compound **36** was slightly less potent, with EC_{50} values of 14 μM, 18 μM, and 17 μM in A431, HeLa, and MDA-MB-231 cells, respectively, and 38 μM in Caco-2 cells. We further evaluated the cytotoxicity of compounds **35**, **36**, and neocucurbol C (**3**) in four additional carcinoma cell

lines: A375-MA2 (melanoma), Ishikawa (endometrial adenocarcinoma), Huh7 (hepatocellular carcinoma), and HCT116 (colorectal carcinoma). Compounds **35** and **36** were most potent in A375-MA2 cells, with EC_{50} values of 3.71 µM and 1.64 µM, respectively (Figure S2). Both also exhibited moderate activity in Ishikawa and HCT116 cells, with EC_{50} values around 5 µM. In contrast, Huh7 cells were the least sensitive, with EC_{50} values of 20–22 µM. Neocucurbol C showed consistently lower potency across all four cell lines, with EC_{50} values greater than 50 µM.

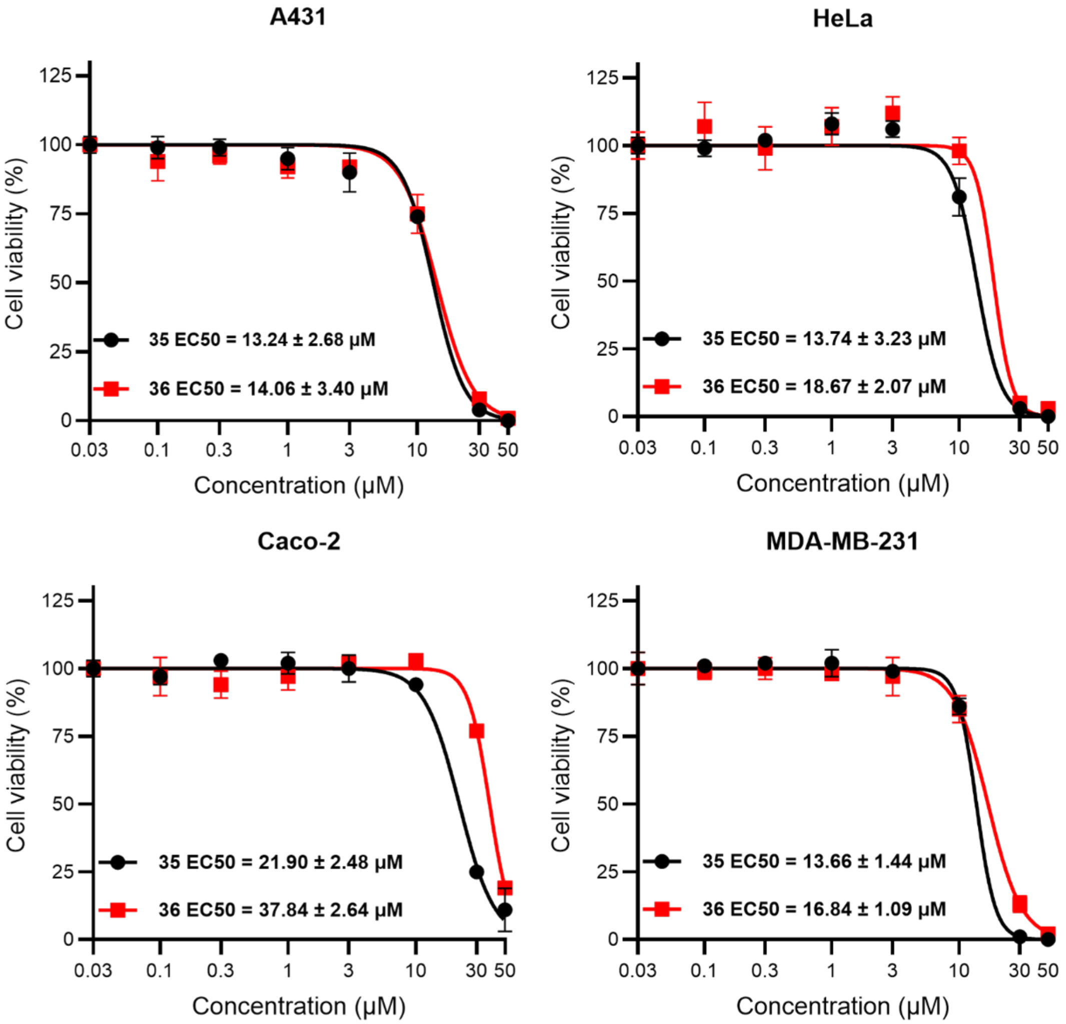

Figure S1. Cytotoxicity EC_{50} curves of compounds **35** and **36** in A431, Caco-2, HeLa, and MDA-MB-231 cells. Cells were treated with 0.5% DMSO, 1 µM staurosporine, and compounds **35** and **36** for 72 hours. After treatment, cells were stained with DAPI and imaged. Data are presented as normalized values ± SD.

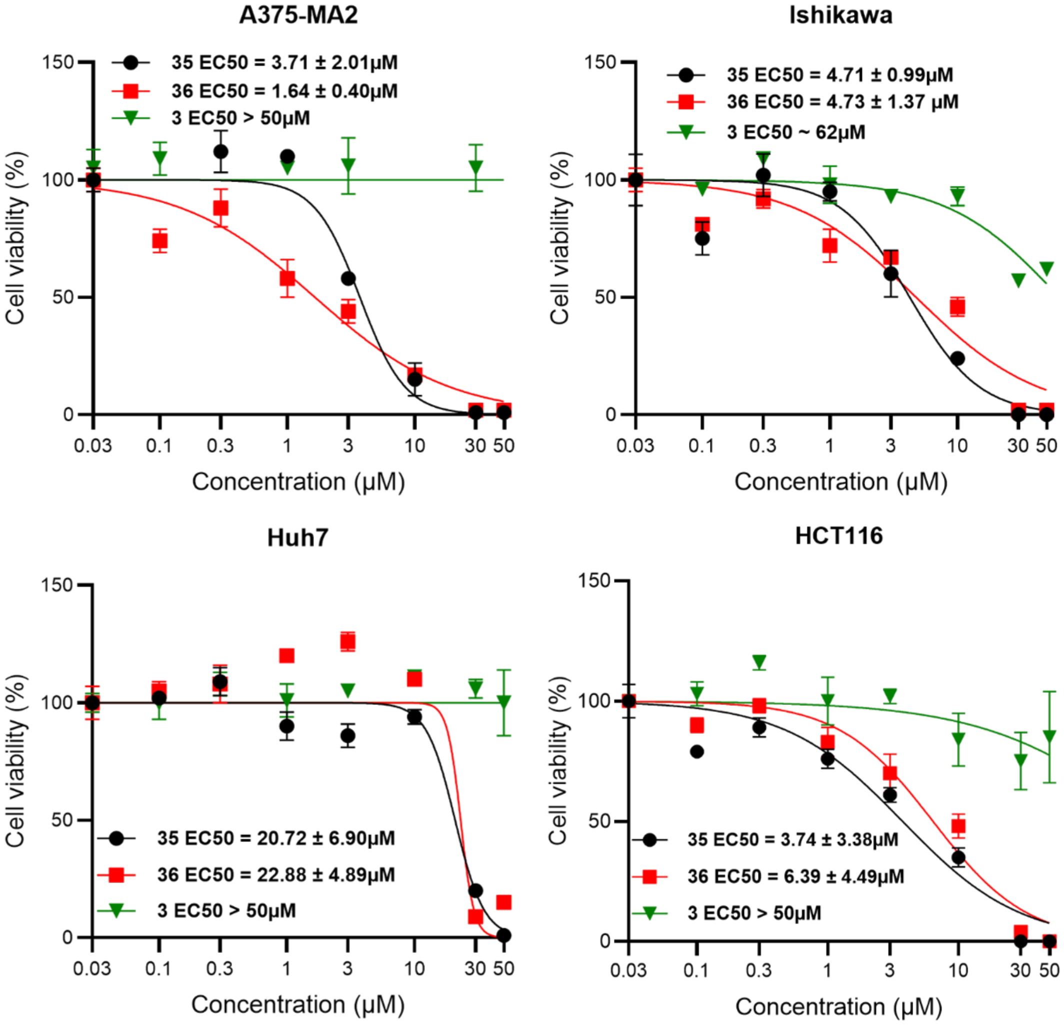

Figure S2. EC_{50} curves of compounds **35**, **36**, and neocucurbol C (**3**) in A375-MA2, Ishikawa, Huh7, and HCT116 cells. Cells were treated with 0.5% DMSO, 1 μM staurosporine, and the compounds for 72 hours. After treatment, cells were stained with DAPI and imaged. Data are presented as normalized values ± SD (n = 3).

Methods

Cell culture

A431, Caco-2, HeLa, MDA-MB-231, A375-MA2, Ishikawa, Huh7, and HCT116 cells were cultured in DMEM containing 10% fetal bovine serum, penicillin-streptomycin, and non-essential amino acids. The cells were incubated at 37°C in a 5% CO_2 atmosphere.

Cytotoxicity assay with DAPI staining

Cells were seeded into 96-well plates using an automated reagent dispenser (Thermo Scientific Multidrop Combi+) at the following densities: 20,000 cells/mL for A431, HeLa, MDA-MB-231, A375-MA2, and Huh7; 40,000 cells/mL for HCT116 and Ishikawa; and 200,000 cells/mL for Caco-2. After overnight incubation at 37°C in a 5% CO_2 atmosphere to allow for cell attachment, treatments were applied: 0.5% DMSO, 1 µM staurosporine, 50 µM neocucurbol C (**3**), and synthetic intermediates (**35–41**, **S18**, **S19**, **S21**) (from 200x DMSO stock solutions). Cells were incubated under standard conditions for 3 days. Post-treatment, cells were washed five times with PBS^{++} (phosphate-buffered saline supplemented with 0.1 g/L calcium and 0.1 g/L magnesium) using a Biotek 406 FX automated washer and dispenser integrated with a Biostack microplate stacker. Fixation was performed with 3.7% formaldehyde for 30 minutes at room temperature, followed by five PBS^{++} washes. Nuclear staining was carried out with 5 µg/mL DAPI in 0.05% saponin for 20 minutes, followed by an additional five PBS^{++} washes. Cells were imaged in 100 µL PBS^{++} using an automated confocal fluorescence microscope (Molecular Devices ImageXpress Confocal HT.ai). Nuclei were quantified using IN Carta software, and data visualization was performed with GraphPad Prism.

Part 5. References

1. Anilkumar, G.; Nambu, H.; Kita, Y. A Simple and Efficient Iodination of Alcohols on Polymer-Supported Triphenylphosphine. *Org. Proc. Res. Dev.* **2002***, 6,* 190–191.

2. Wang, Y.; Sun, W.; Lu, R.; Wen, Z.; Yao, J.; Li, H. Inorganic Bases Enhanced Organocatalysis for Aerobic α-Hydroxylation of Aliphatic Cycloketones. *Asian Journal of Organic Chemistry.* **2022**, *11*, e202200443.

3. Tanino, K.; Onuki, K.; Asano, K.; Miyashita, M.; Nakamura, T.; Takahashi, Y.; Kuwajima, I. Total Synthesis of Ingenol. *J. Am. Chem. Soc.* **2003**, *125*, 1498−1500.

4. Alvarez-Manzaneda, E. J.; Chahboun, R.; Torres, E. C.; Alvarez, E.; Alvarez-Manzaneda, R.; Haidour, A.; Ramos López, J. M. Reaction of allylic and benzylic alcohols and esters with PPh_3/I_2: one-pot synthesis of β,γ-unsaturated compounds. *Tetrahedron Lett.* **2005**, *46*, 3755−3759.

5. Smaligo, A. J.; Swain, M.; Quintana, J. C.; Tan, M.; Kim, D. A.; Kwon, O. Hydrodealkenylative C(sp3)–C(sp2) bond fragmentation *Science* ***2019****, 364,* 681–685.

6. Hu, J.; Zou, Z.; Chen, Y.; Li, S.; Gao, X.; Liu, Z.; Wang, Y.; Liu, H.; Zhang, W. Neocucurbols A−H, Phomactin Diterpene Derivatives from the Marine-Derived Fungus Neocucurbitaria unguis-hominis FS685. *J. Org. Chem.* **1993**, *58*, 11−13. (b) Piers, E.; Renaud, J.; Rettig, S. J. *J. Nat. Prod.* **2022***, 85,* 1967−1975.

7. Lagoutte, R.; Serba, C.; Abegg, D.; Hoch, D. G.; Adibekian, A.; Winssinger, N., Divergent synthesis and identification of the cellular targets of deoxyelephantopins. *Nat. Commun.* **2016**, *7*, 12470.

8. Chae, H. J.; Kang, J. S.; Byun, J. O.; Han, K. S.; Kim, D. U.; Oh, S. M.; Kim, H. M.; Chae, S. W.; Kim, H. R., Molecular mechanism of staurosporine-induced apoptosis in osteoblasts. *Pharmacol. Res.* **2000**, *42*, 373−81.

9. Falcieri, E.; Martelli, A. M.; Bareggi, R.; Cataldi, A.; Cocco, L., The protein kinase inhibitor staurosporine induces morphological changes typical of apoptosis in MOLT-4 cells without concomitant DNA fragmentation. *Biochem. Biophys. Res. Commun.* **1993**, *193*, 19−25.

Part 6. ^{1}H and ^{13}C NMR spectra

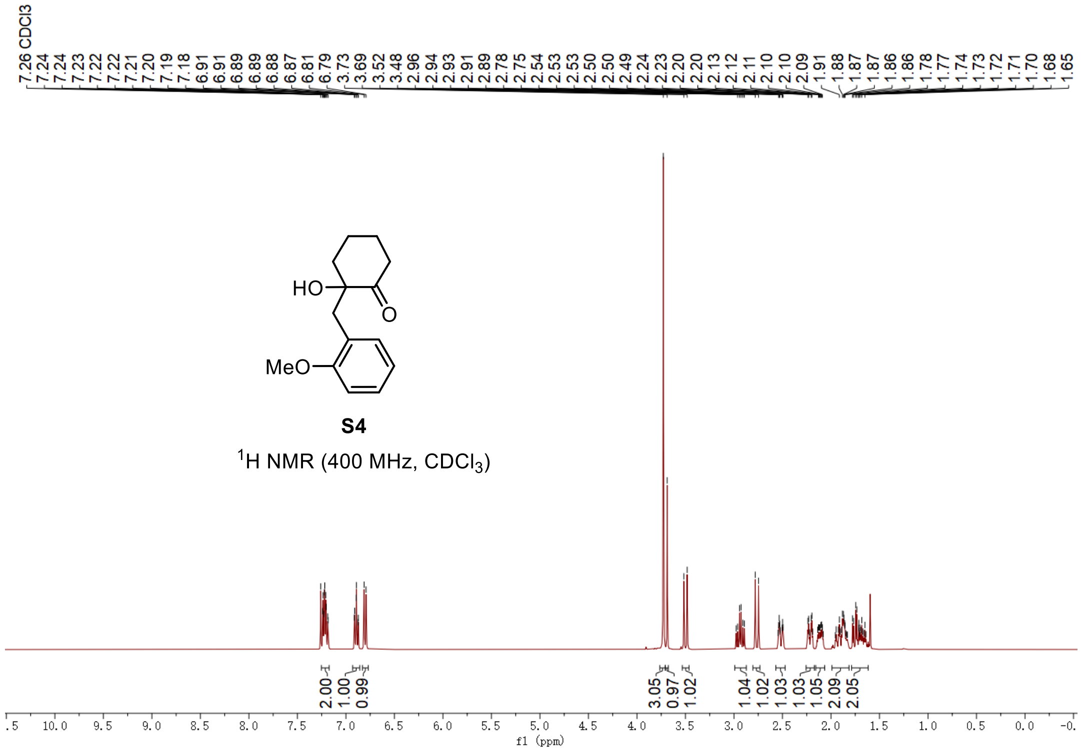

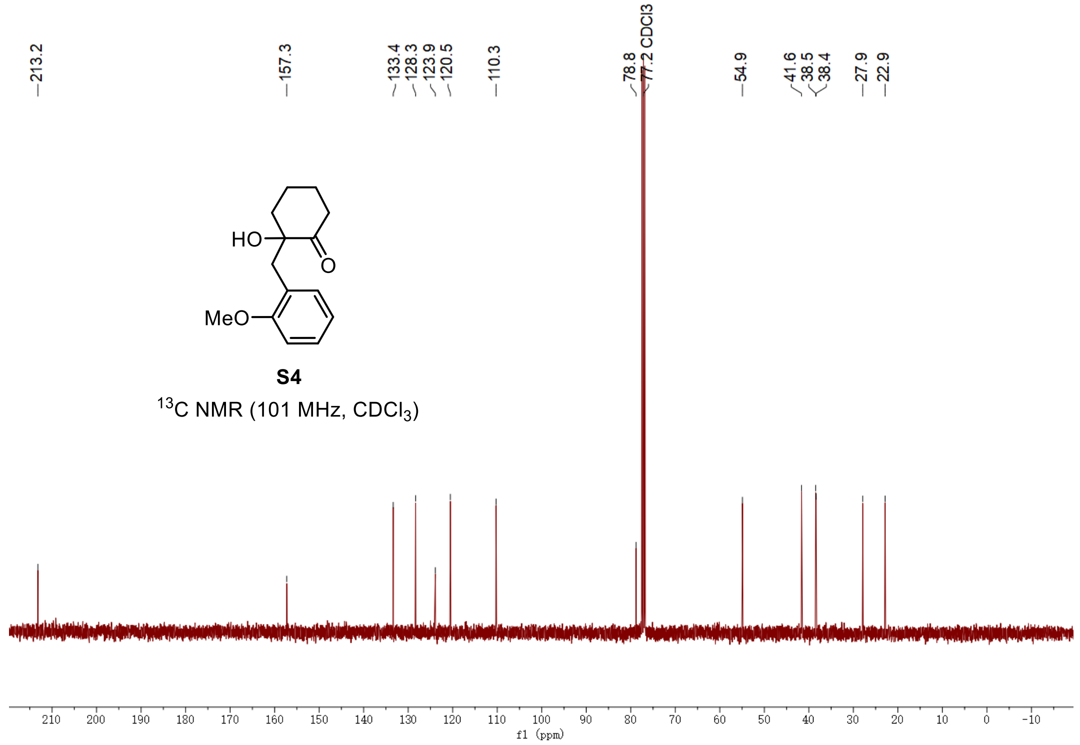

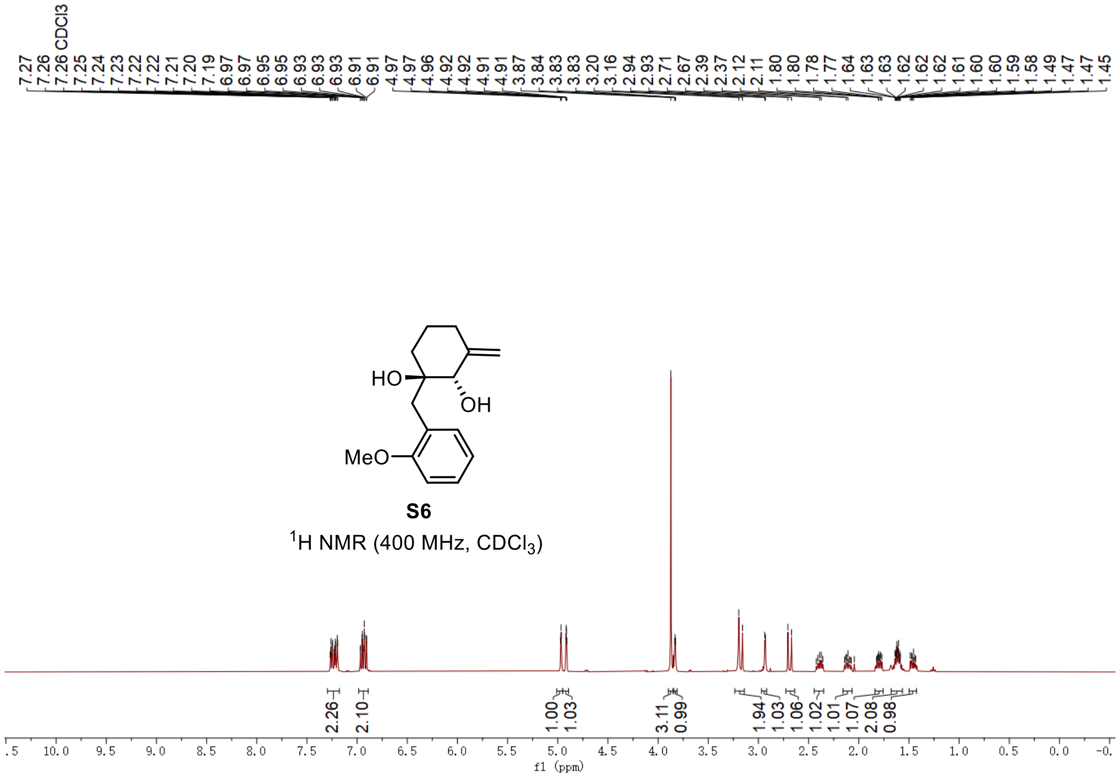
7.27 7.26 CDCl3 7.25 7.24 7.23 7.22 7.21 7.20 7.19 6.97 6.97 6.95 6.95 6.93 6.93 6.93 6.91 6.91 4.97 4.97 4.96 4.92 4.92 4.91 4.91 4.91 3.87 3.84 3.83 3.83 3.20 3.16 2.94 2.93 2.71 2.67 2.39 2.37 2.12 2.11 1.80 1.80 1.78 1.77 1.64 1.63 1.63 1.62 1.62 1.62 1.61 1.60 1.60 1.59 1.58 1.49 1.47 1.45
HO
OH
MeO
S6
¹H NMR (400 MHz, CDCl₃)
2.26 2.10 1.00 1.03 3.11 0.99 1.94 1.03 1.06 1.02 1.01 1.07 2.08 0.98
f1 (ppm)

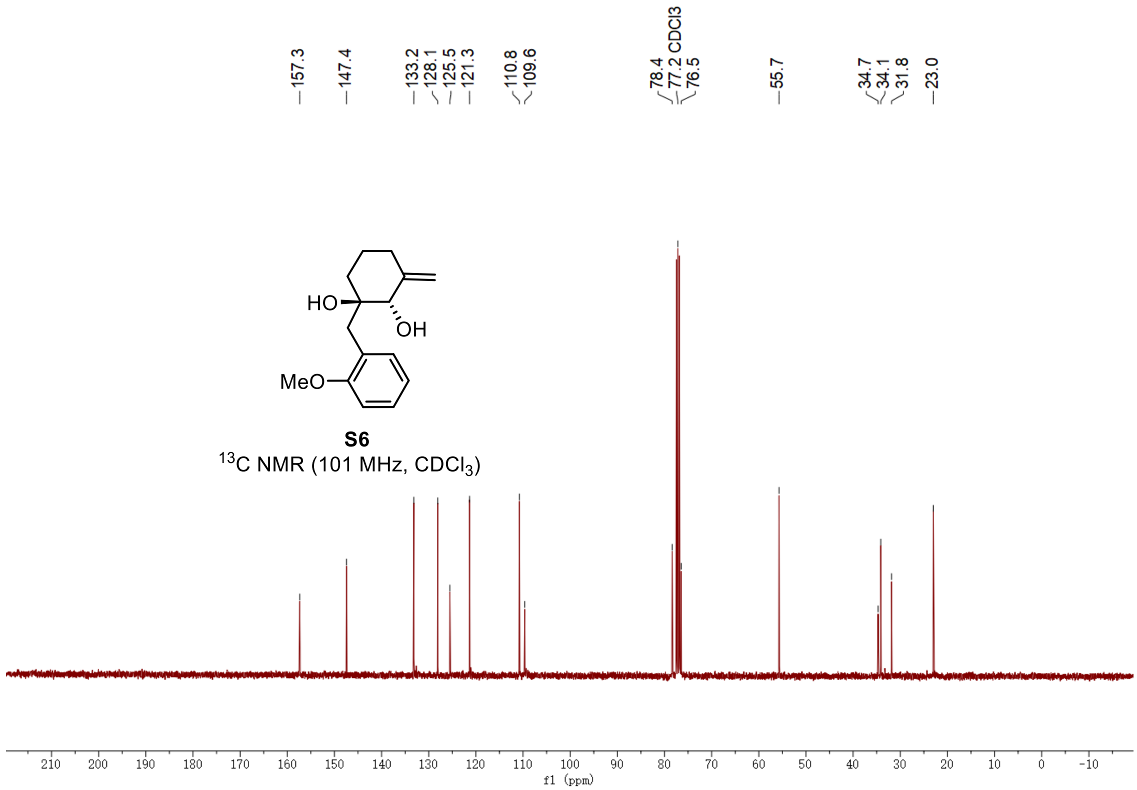
157.3 147.4 133.2 128.1 125.5 121.3 110.8 109.6 78.4 77.2 CDCl3 76.5 55.7 34.7 34.1 31.8 23.0
HO
OH
MeO
S6
¹³C NMR (101 MHz, CDCl₃)
f1 (ppm)

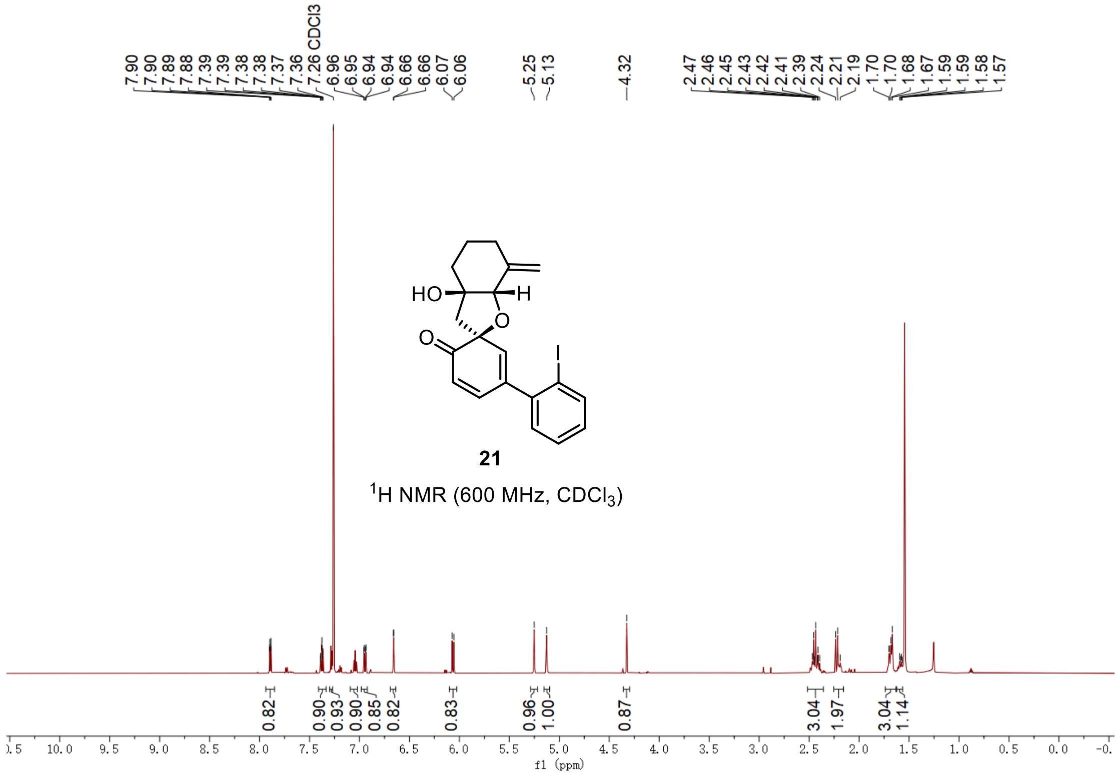

21

^{1}H NMR (600 MHz, $CDCl_3$)

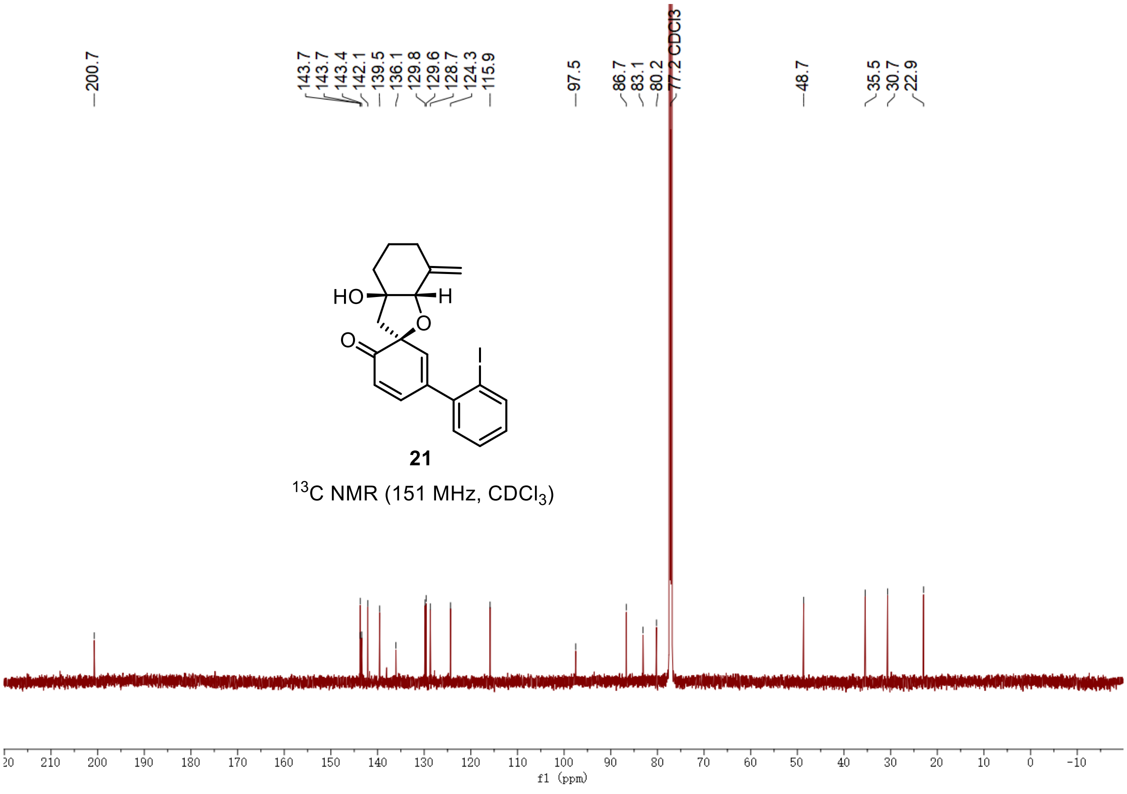

21

^{13}C NMR (151 MHz, $CDCl_3$)

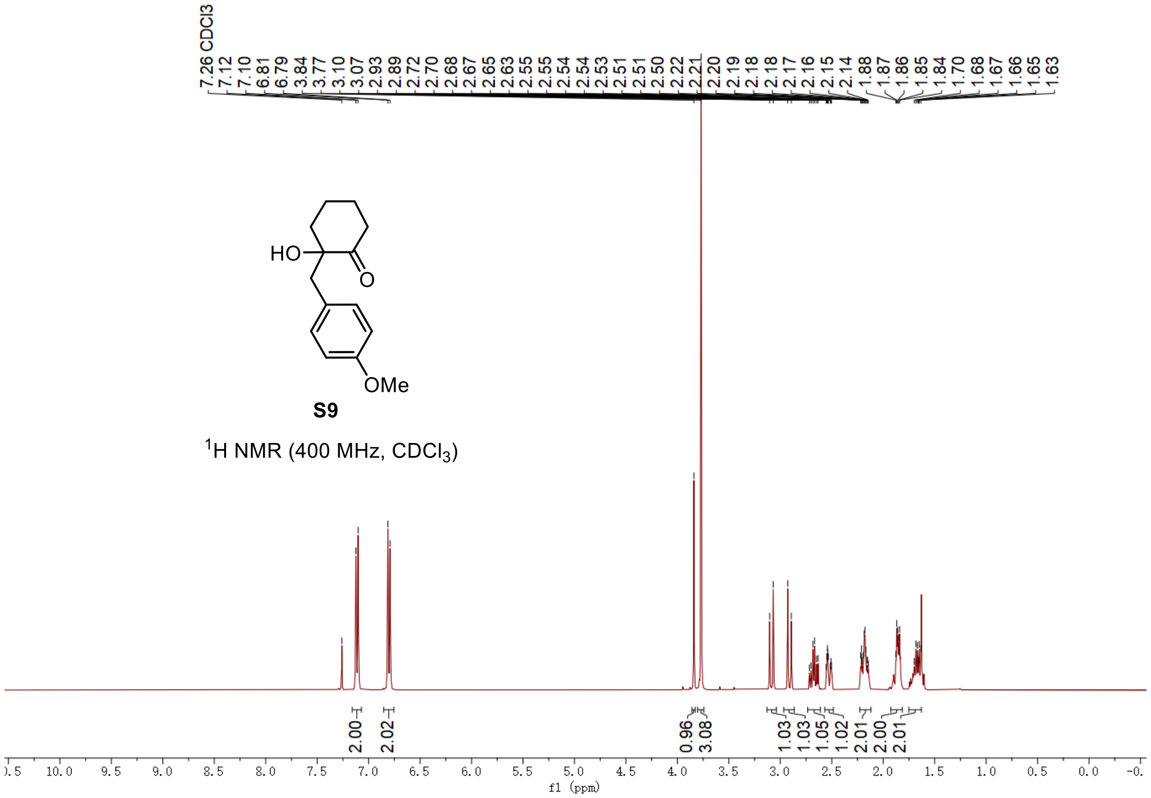
7.26 CDCl3
7.12
7.10
6.81
6.79
3.84
3.77
3.10
3.07
2.93
2.89
2.72
2.70
2.68
2.67
2.65
2.63
2.55
2.54
2.54
2.53
2.51
2.51
2.50
2.22
2.21
2.20
2.19
2.18
2.18
2.17
2.16
2.15
2.14
1.88
1.87
1.86
1.85
1.84
1.70
1.68
1.67
1.66
1.65
1.63
HO
O
OMe
S9
¹H NMR (400 MHz, CDCl₃)
2.00
2.02
0.96
3.08
1.03
1.03
1.05
1.02
2.01
2.00
2.01
f1 (ppm)

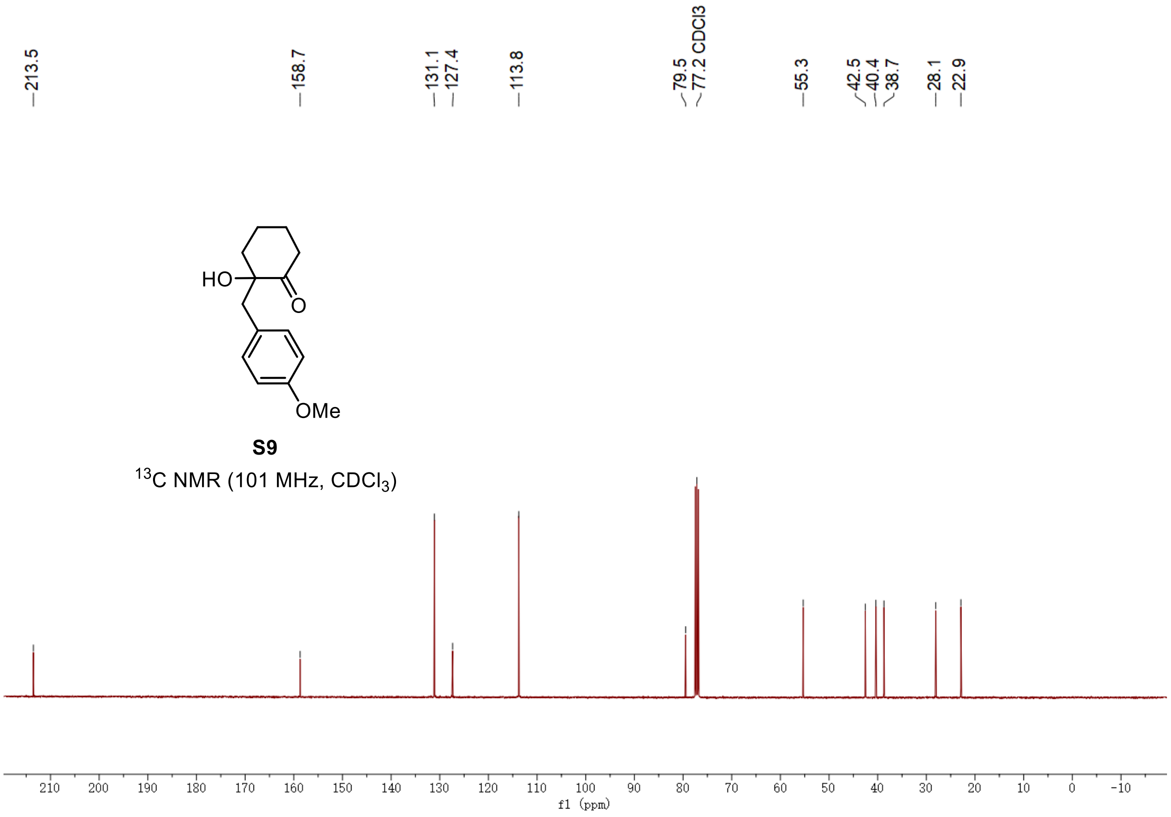
213.5
158.7
131.1
127.4
113.8
79.5
77.2 CDCl3
55.3
42.5
40.4
38.7
28.1
22.9
HO
O
OMe
S9
¹³C NMR (101 MHz, CDCl₃)
f1 (ppm)

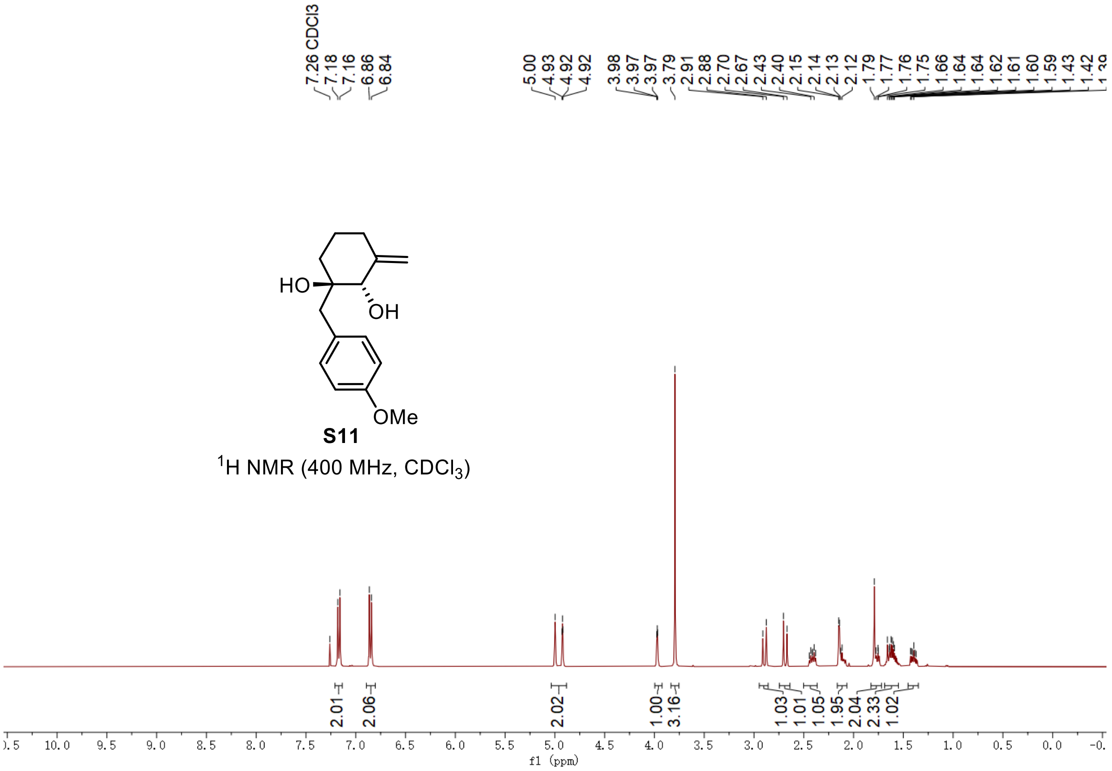

S11

^{1}H NMR (400 MHz, $CDCl_3$)

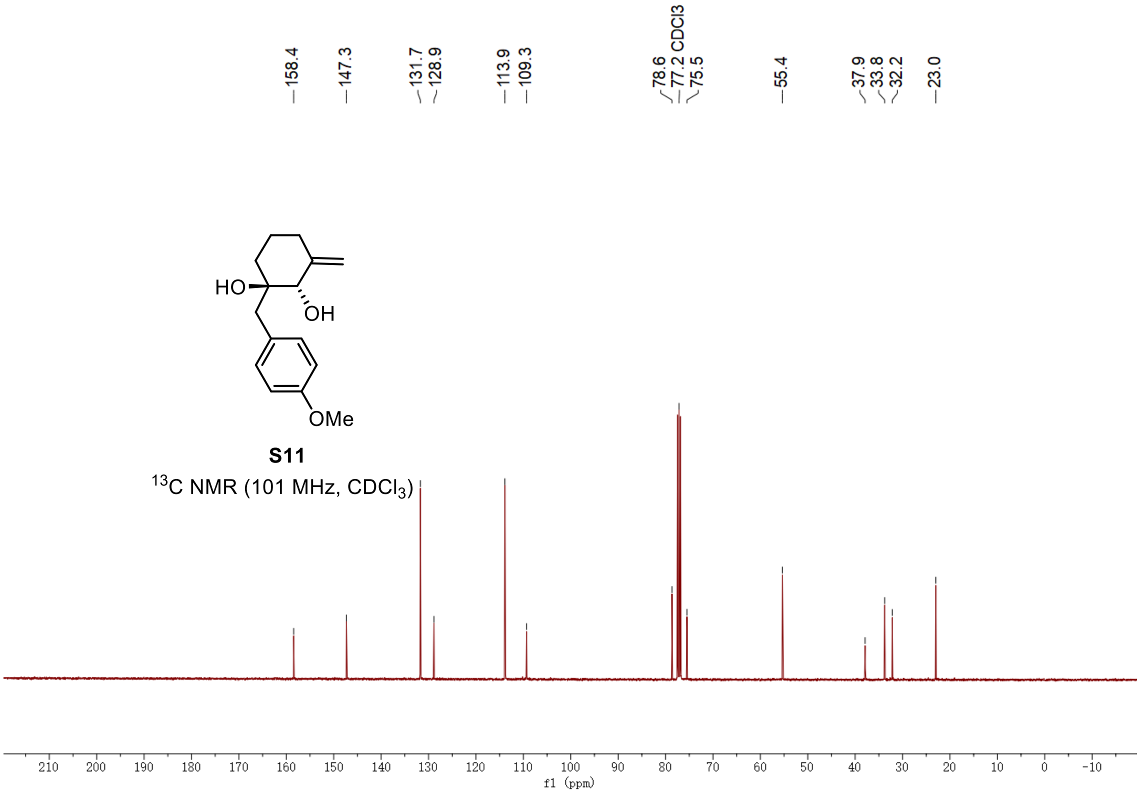

S11

^{13}C NMR (101 MHz, $CDCl_3$)

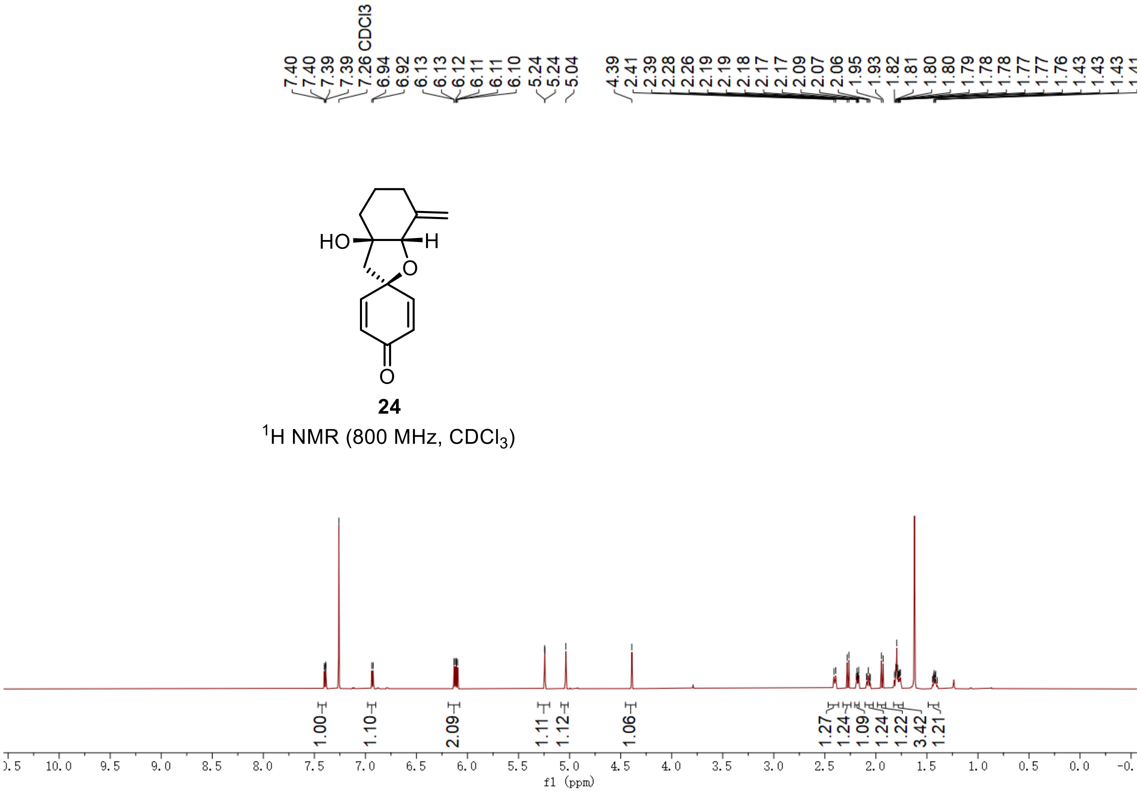

24

^{1}H NMR (800 MHz, $CDCl_3$)

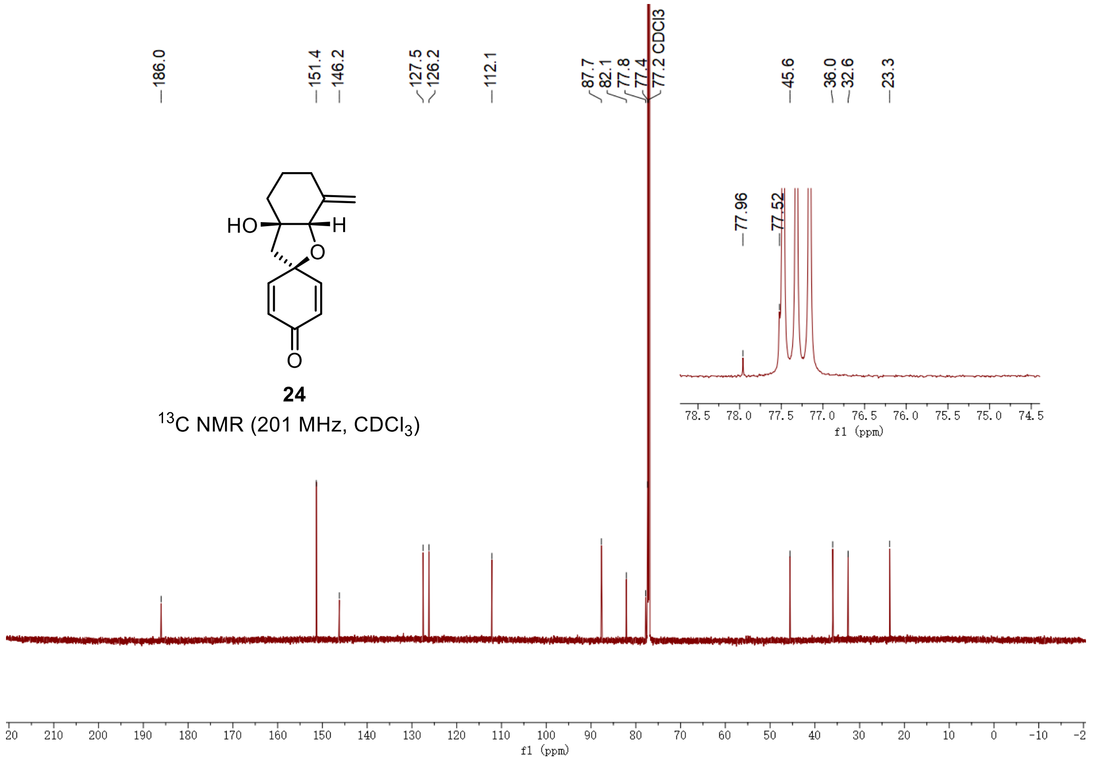

24

^{13}C NMR (201 MHz, $CDCl_3$)

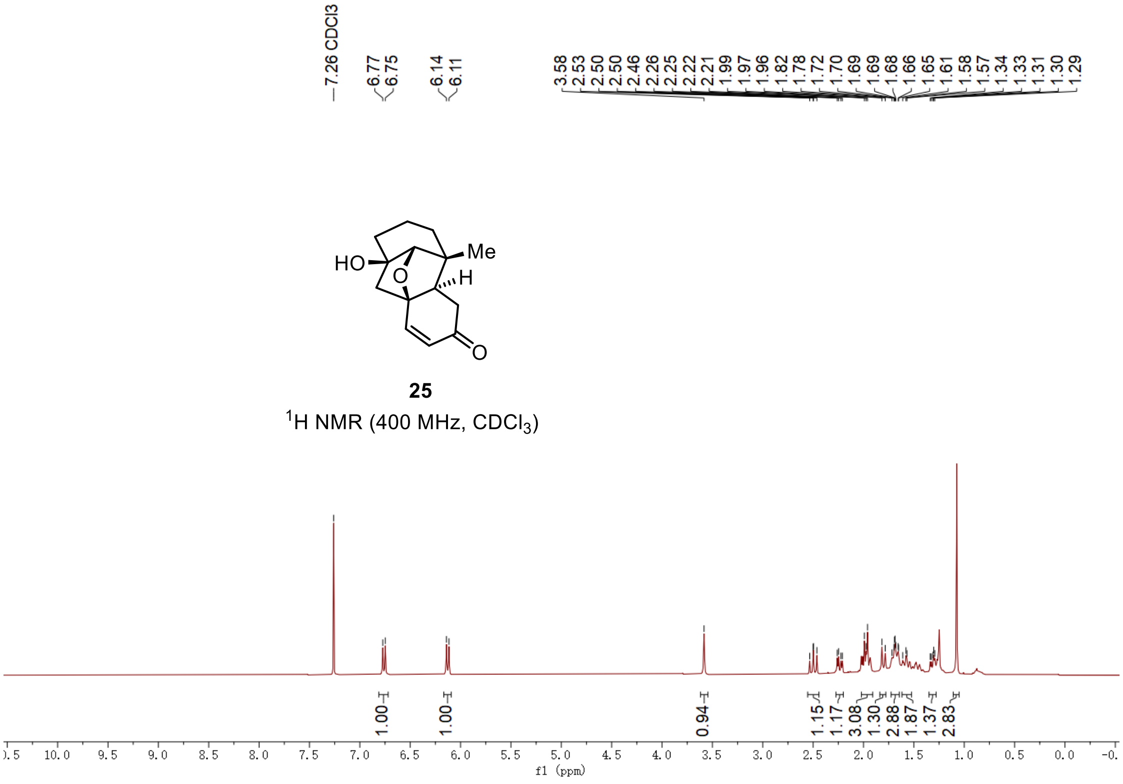

25

^{1}H NMR (400 MHz, $CDCl_3$)

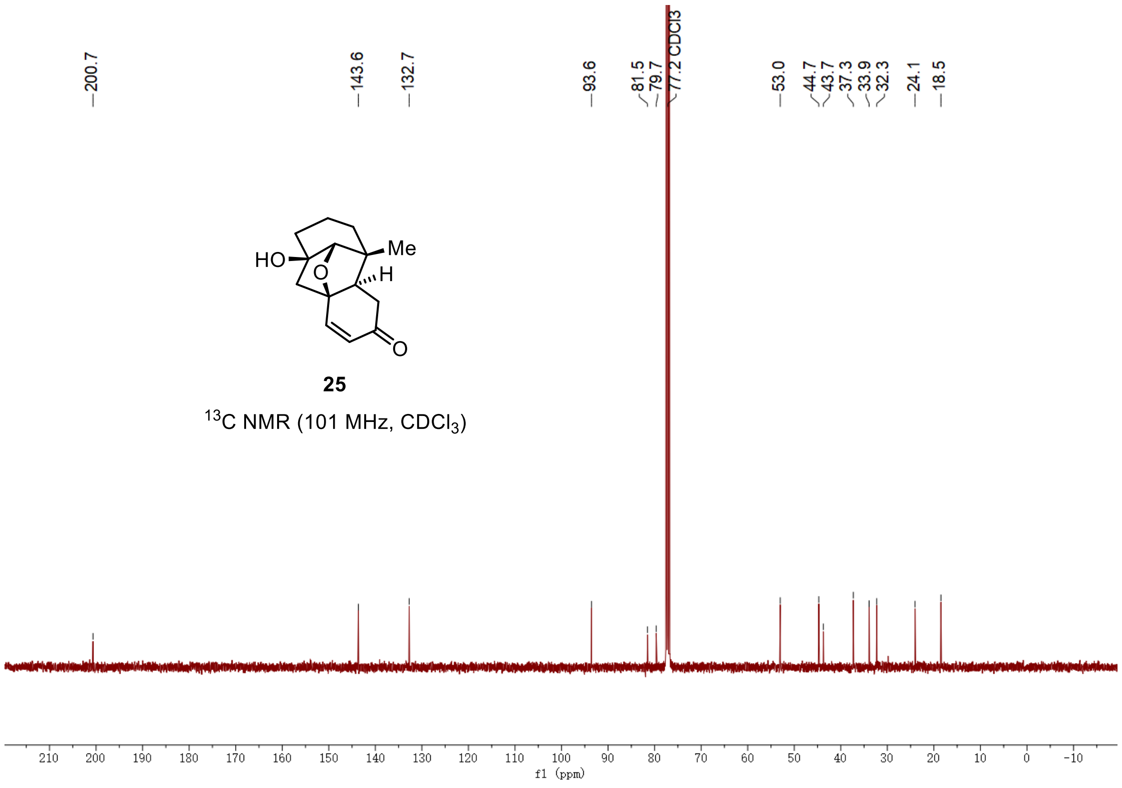

25

^{13}C NMR (101 MHz, $CDCl_3$)

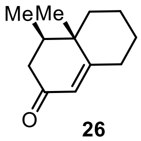

26

^{1}H NMR (400 MHz, $CDCl_3$)

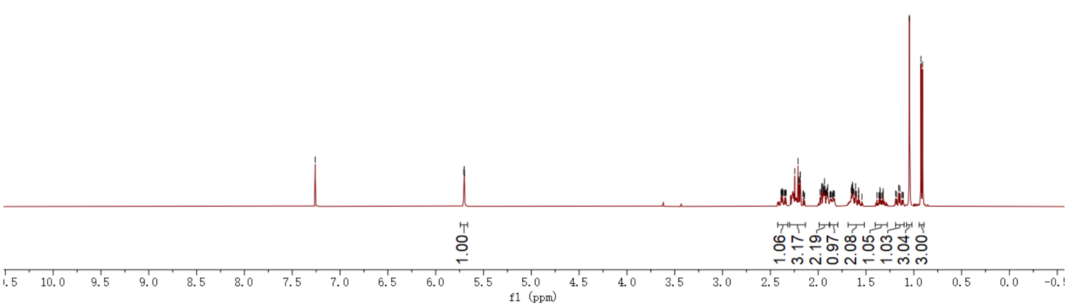

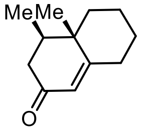

26

^{13}C NMR (101 MHz, $CDCl_3$)

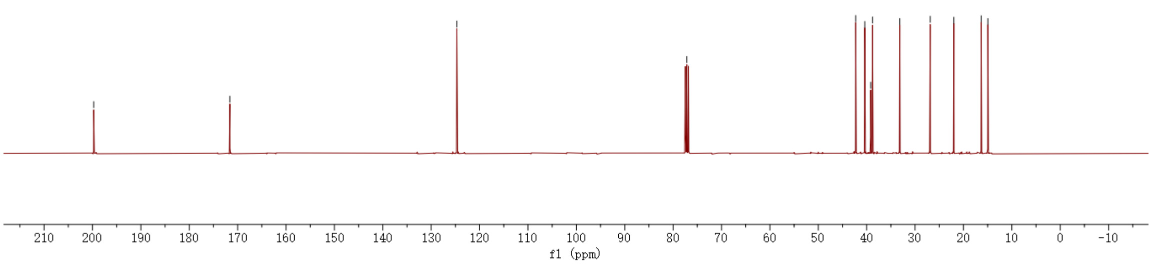

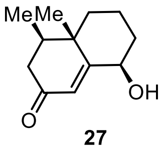

27

^{1}H NMR (400 MHz, $CDCl_3$)

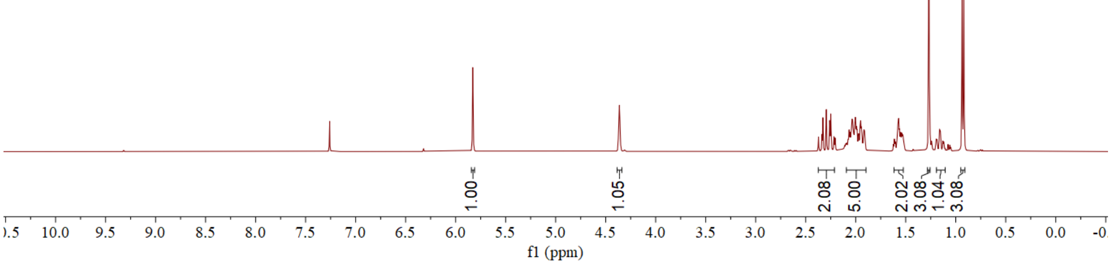

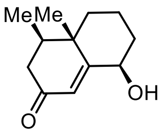

27

^{13}C NMR (101 MHz, $CDCl_3$)

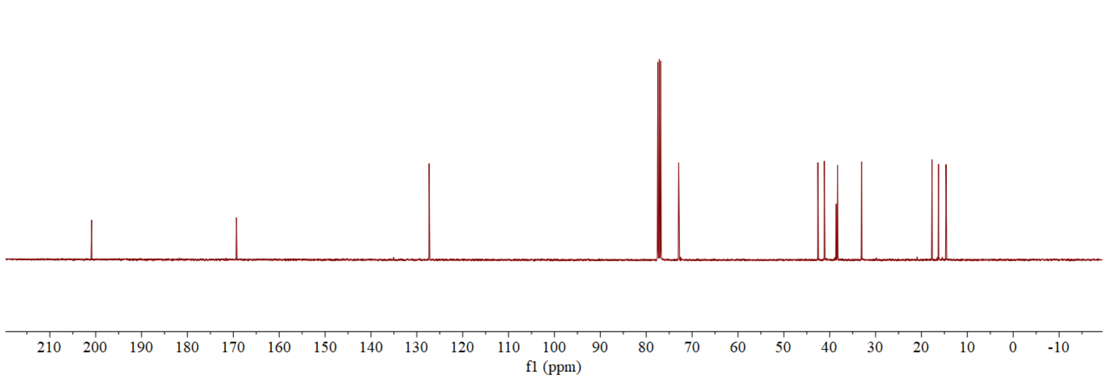

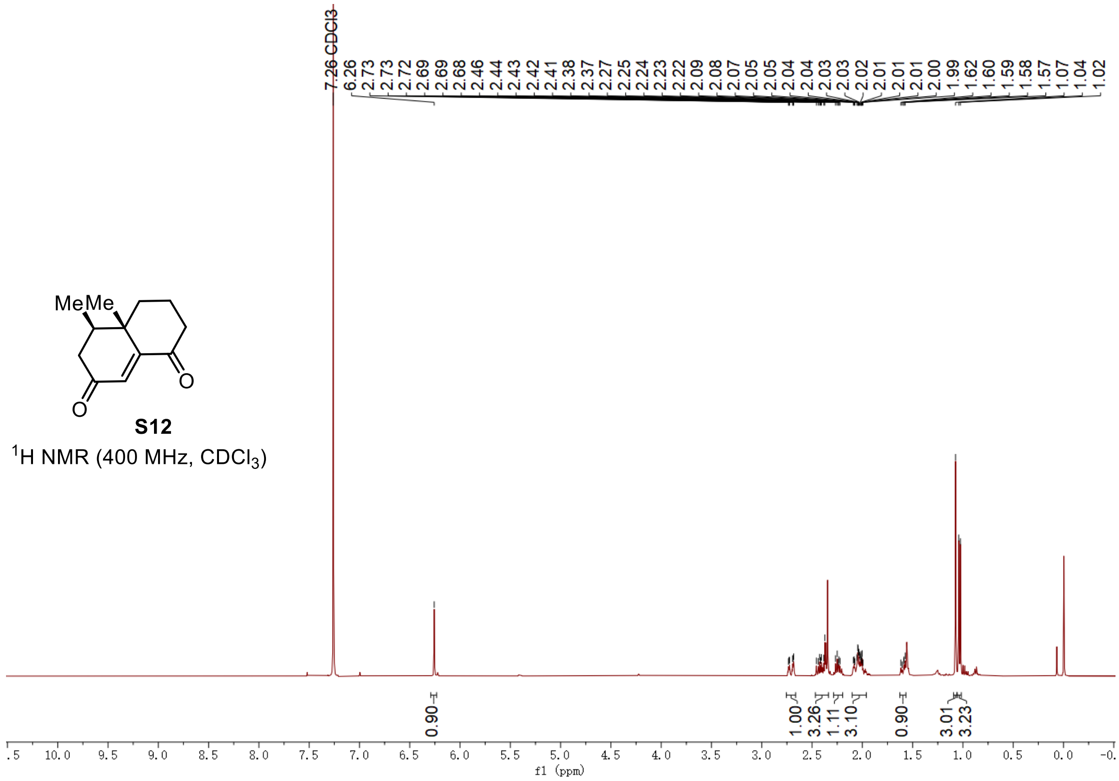

S12

^{1}H NMR (400 MHz, $CDCl_3$)

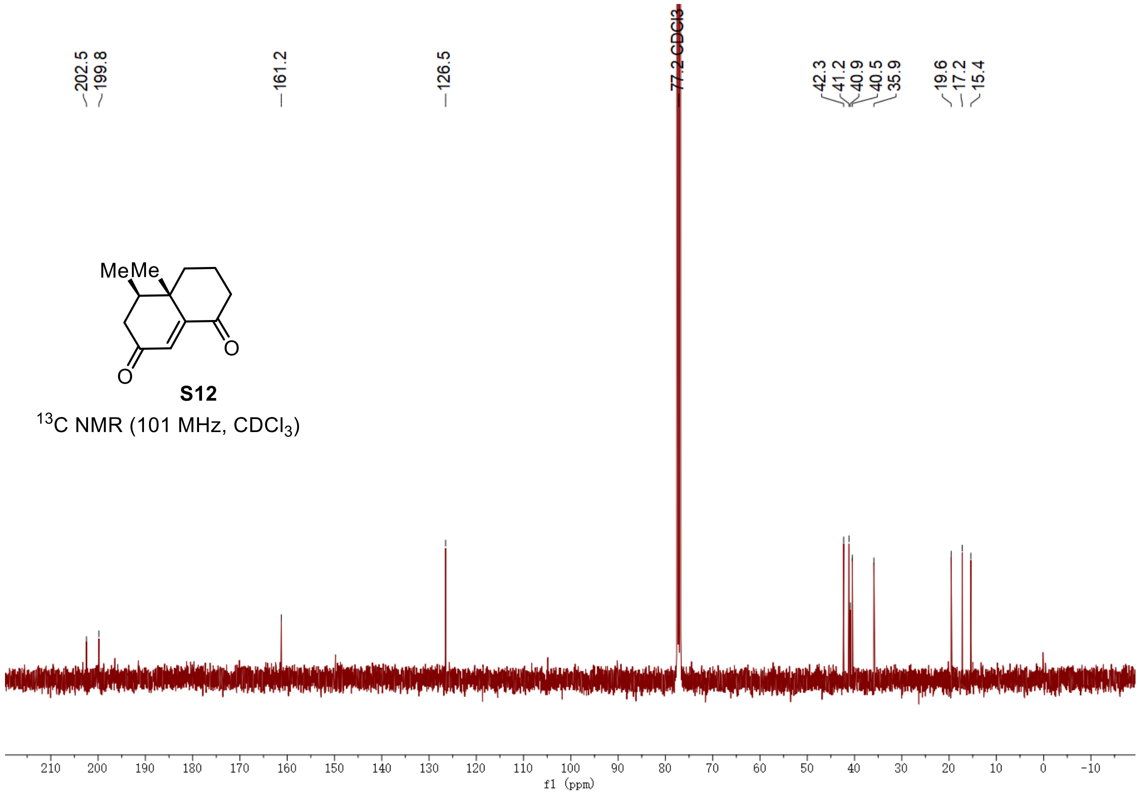

S12

^{13}C NMR (101 MHz, $CDCl_3$)

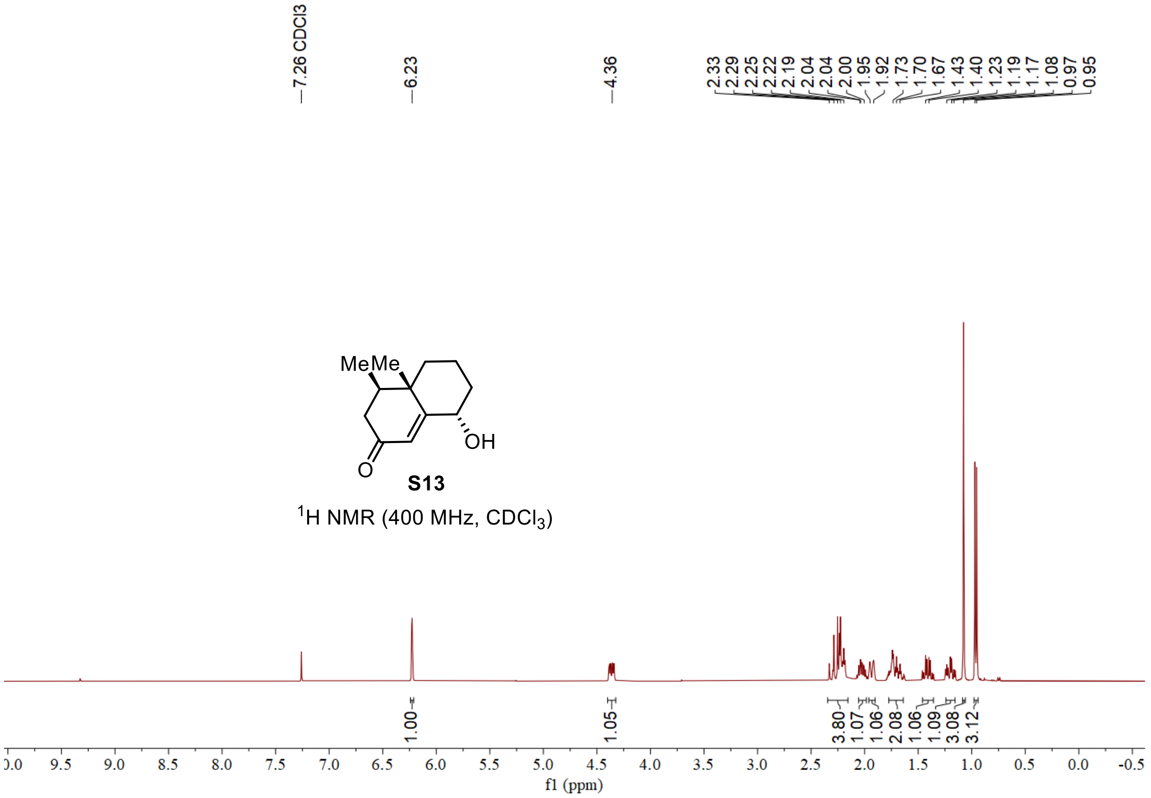
7.26 CDCl3
6.23
4.36
2.33
2.29
2.25
2.22
2.19
2.04
2.04
2.00
1.95
1.92
1.73
1.70
1.67
1.43
1.40
1.23
1.19
1.17
1.08
0.97
0.95
MeMe
OH
O
S13
1H NMR (400 MHz, CDCl3)
1.00
1.05
3.80
1.07
1.06
2.08
1.06
1.09
3.08
3.12
f1 (ppm)

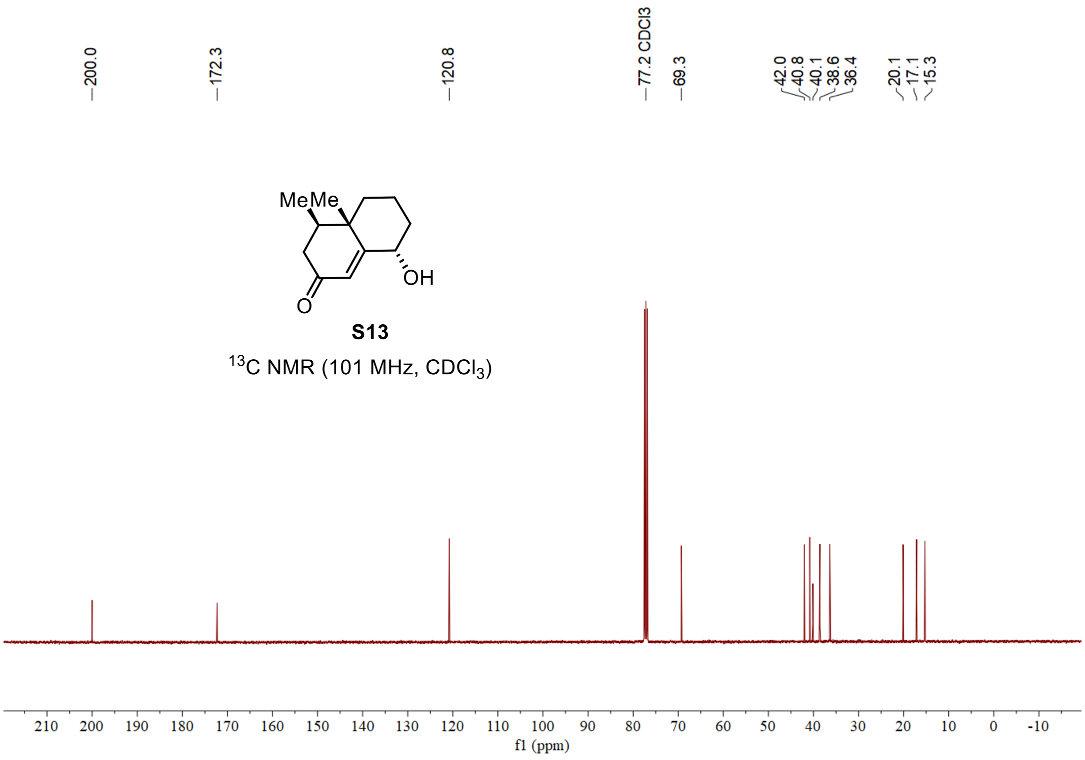
200.0
172.3
120.8
77.2 CDCl3
69.3
42.0
40.8
40.1
38.6
36.4
20.1
17.1
15.3
MeMe
OH
O
S13
13C NMR (101 MHz, CDCl3)
f1 (ppm)

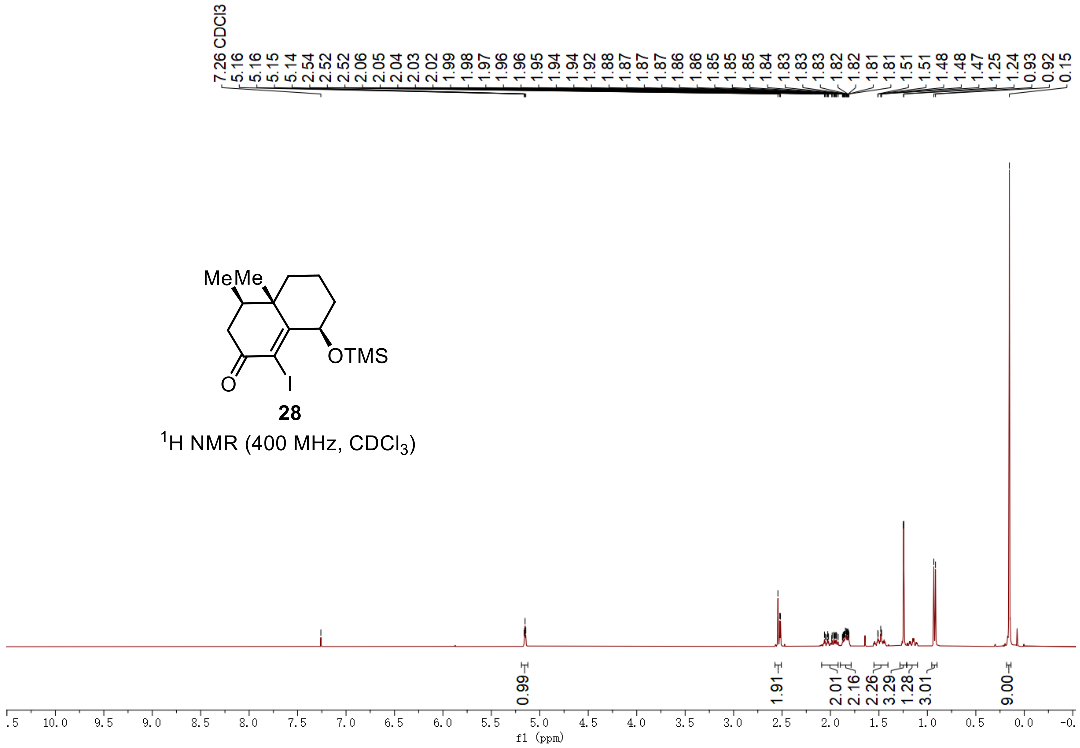

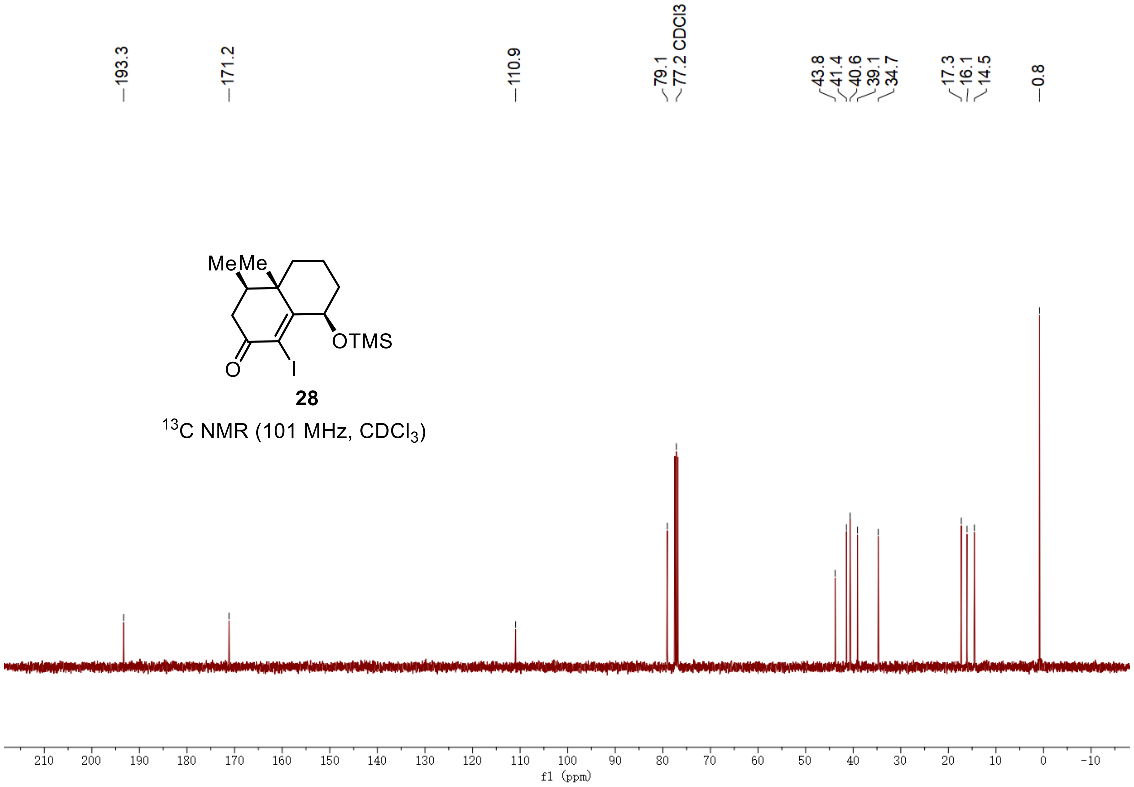

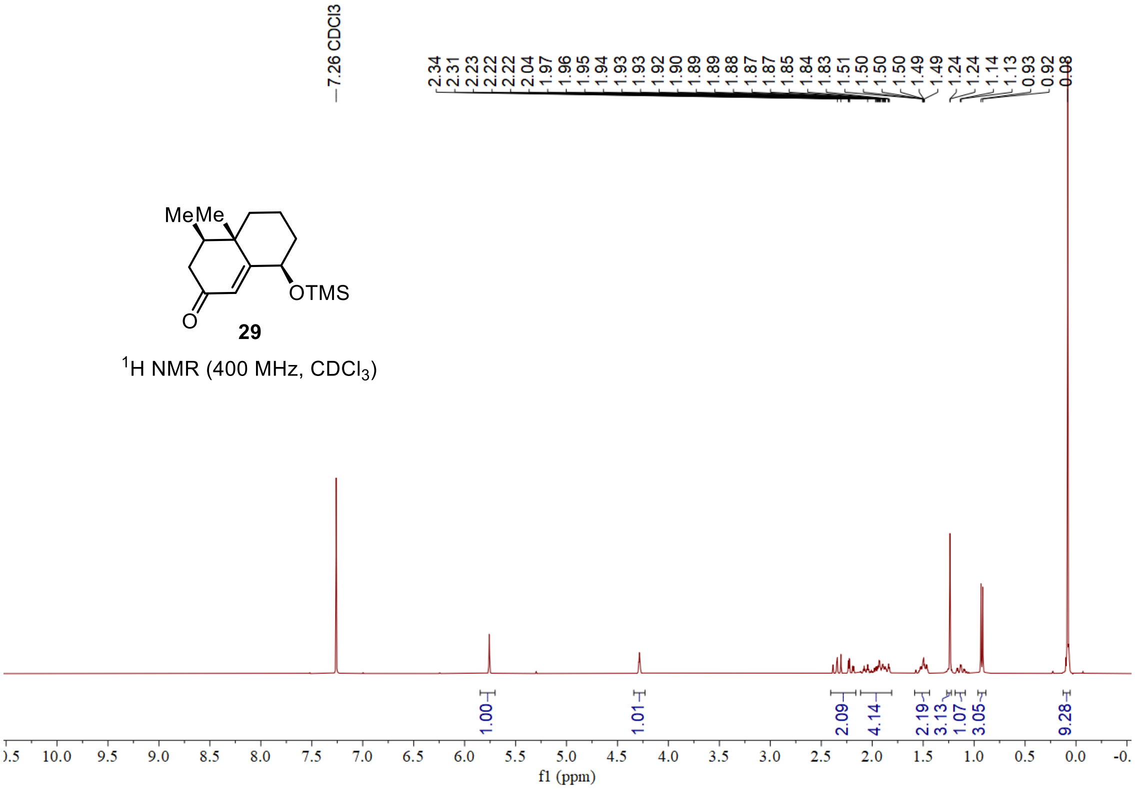
7.26 CDCl3
2.34
2.31
2.23
2.22
2.22
2.04
1.97
1.96
1.95
1.94
1.93
1.93
1.92
1.90
1.89
1.89
1.88
1.87
1.87
1.85
1.84
1.83
1.51
1.50
1.50
1.50
1.49
1.49
1.24
1.24
1.14
1.13
0.93
0.92
0.08
MeMe
OTMS
O
29
1H NMR (400 MHz, CDCl3)
1.00
1.01
2.09
4.14
2.19
3.13
1.07
3.05
9.28
f1 (ppm)

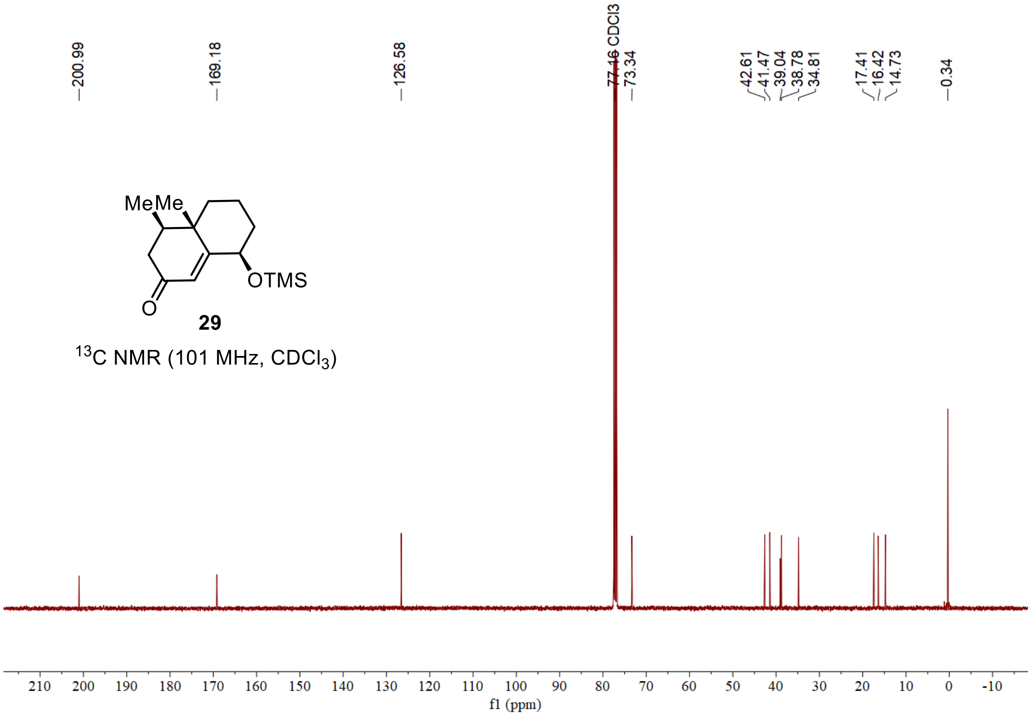
200.99
169.18
126.58
77.46 CDCl3
73.34
42.61
41.47
39.04
38.78
34.81
17.41
16.42
14.73
0.34
MeMe
OTMS
O
29
13C NMR (101 MHz, CDCl3)
f1 (ppm)

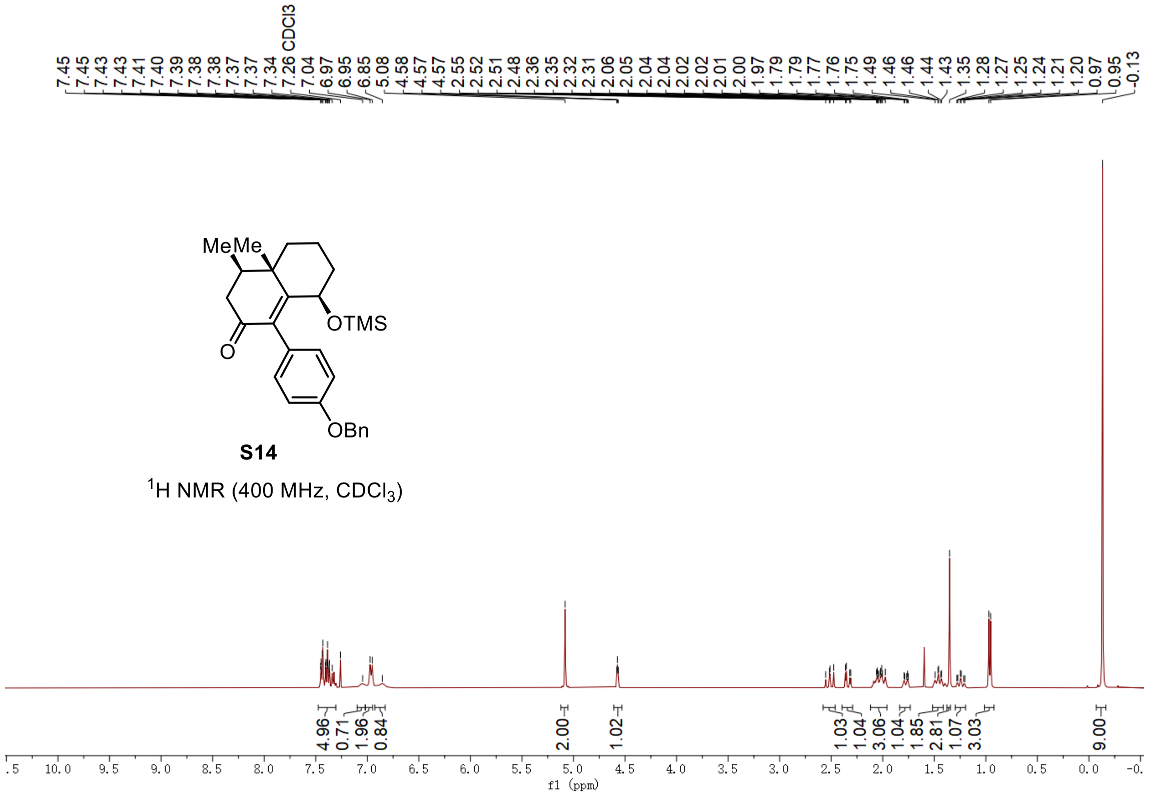

S14

^{1}H NMR (400 MHz, $CDCl_3$)

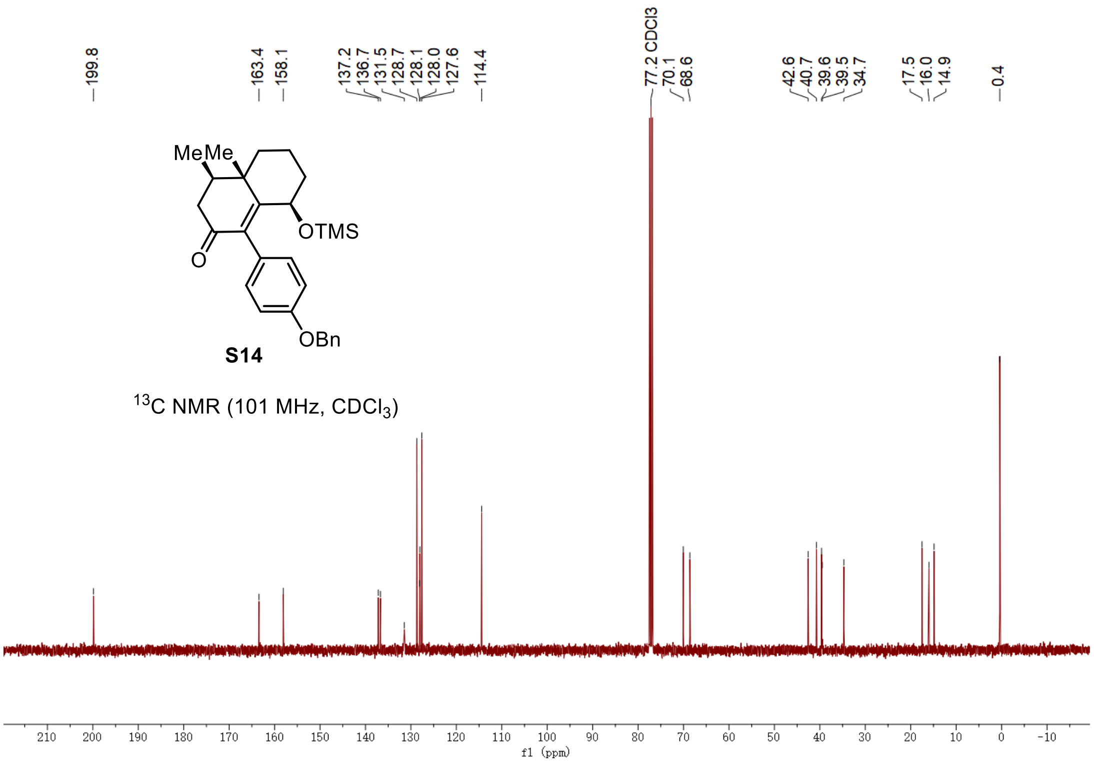

S14

^{13}C NMR (101 MHz, $CDCl_3$)

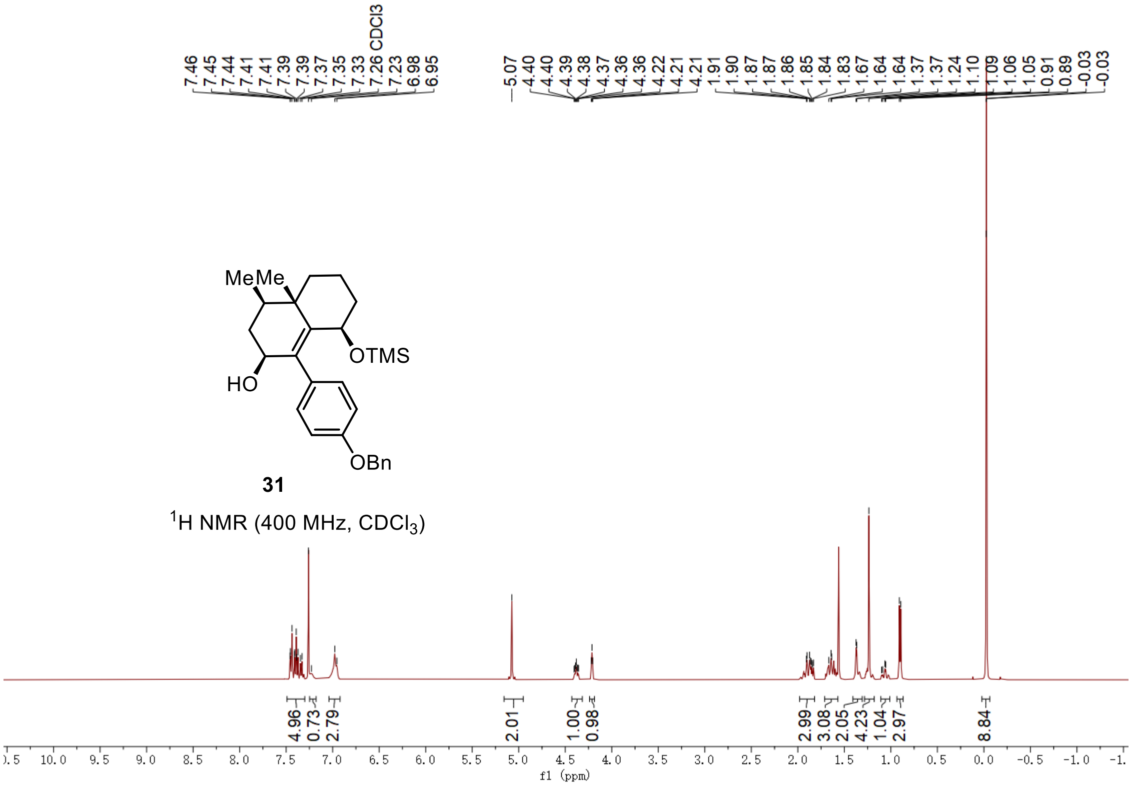

31

^{1}H NMR (400 MHz, $CDCl_3$)

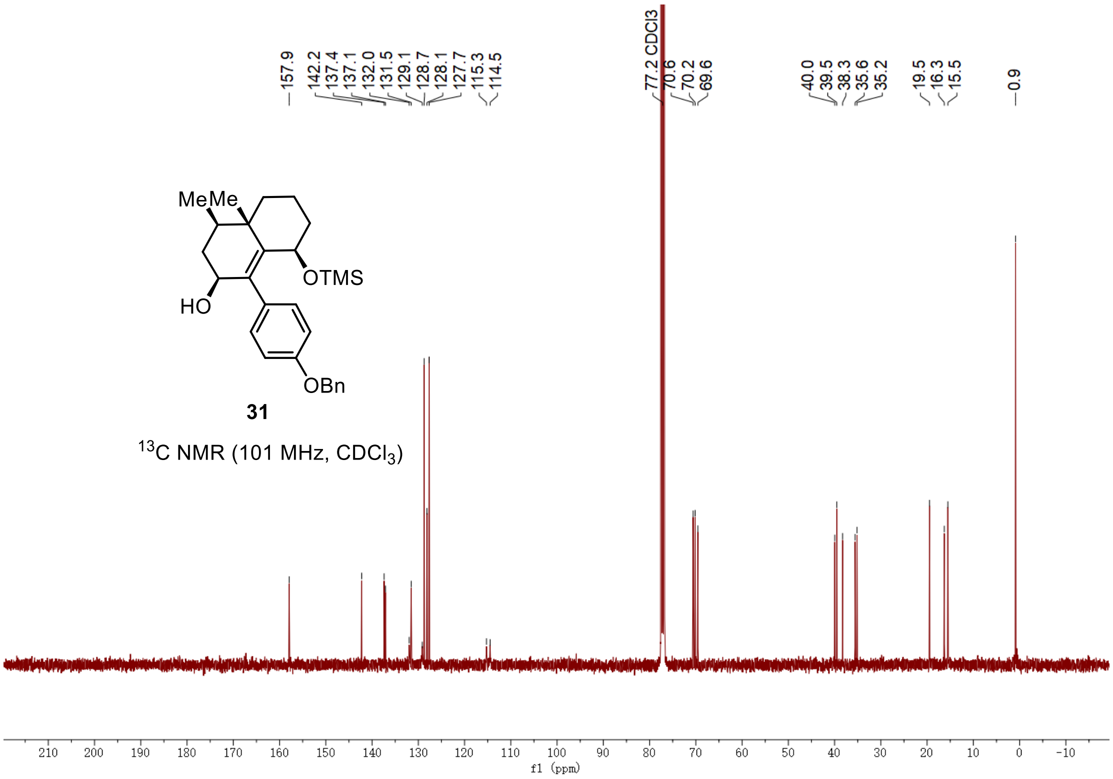

31

^{13}C NMR (101 MHz, $CDCl_3$)

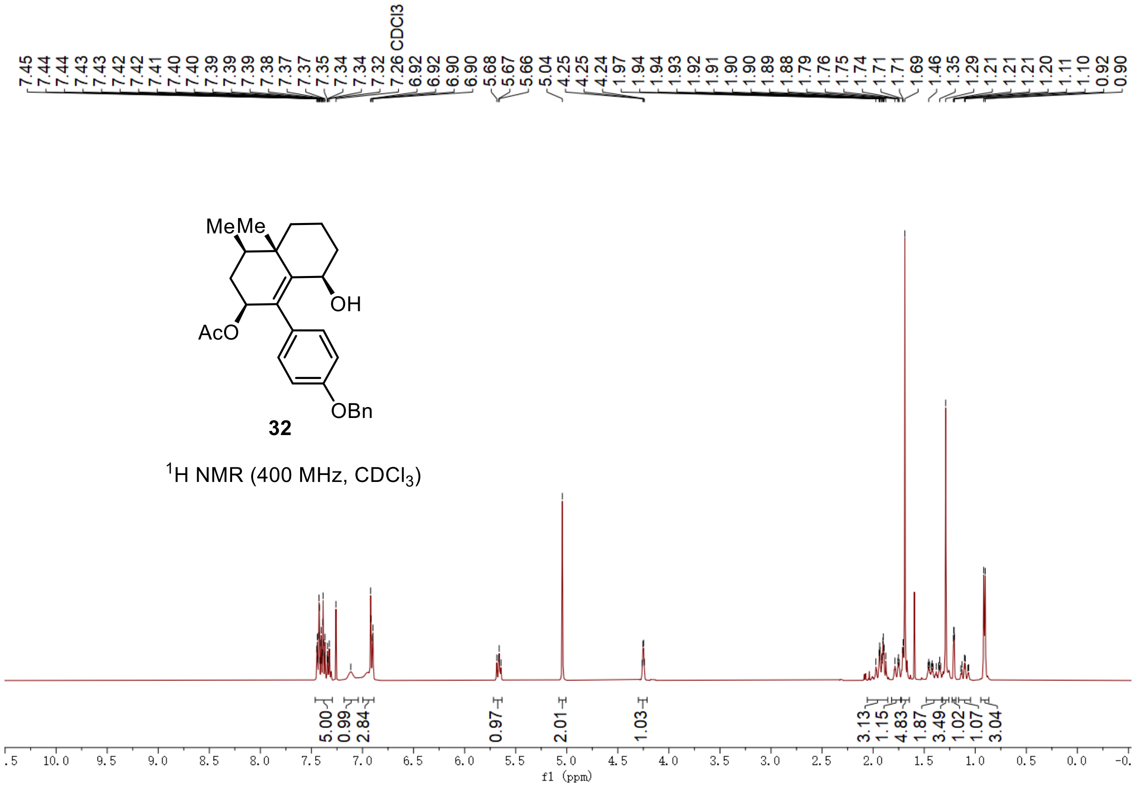
7.45 7.44 7.44 7.43 7.43 7.42 7.42 7.41 7.40 7.40 7.39 7.39 7.39 7.38 7.37 7.37 7.35 7.34 7.34 7.32 7.26 CDCl3 6.92 6.92 6.92 6.90 6.90 5.68 5.67 5.66 5.04 4.25 4.25 4.24 1.97 1.94 1.94 1.93 1.92 1.91 1.90 1.90 1.89 1.88 1.79 1.76 1.75 1.74 1.71 1.71 1.69 1.46 1.35 1.29 1.21 1.21 1.20 1.11 1.10 0.92 0.90
MeMe
OH
AcO
OBn
32
1H NMR (400 MHz, CDCl3)
5.00 0.99 2.84 0.97 2.01 1.03 3.13 1.15 4.83 1.87 3.49 1.02 1.07 3.04
f1 (ppm)

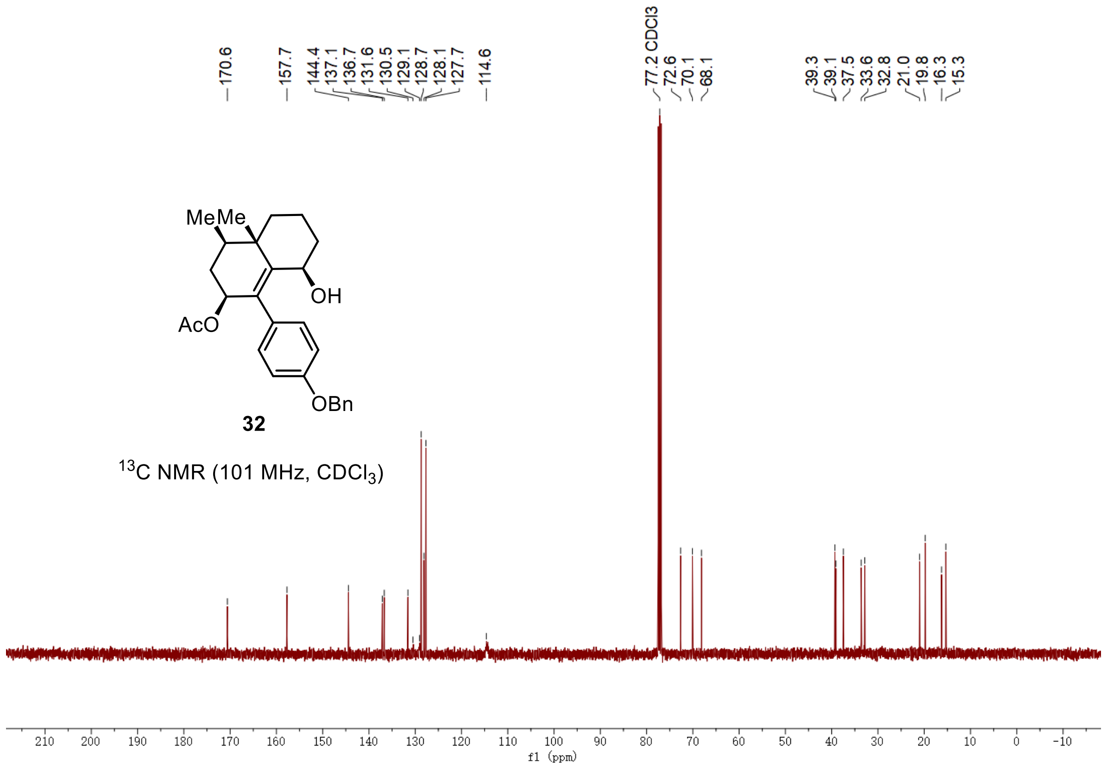
170.6 157.7 144.4 137.1 136.7 131.6 130.5 129.1 128.7 128.1 127.7 114.6 77.2 CDCl3 72.6 70.1 68.1 39.3 39.1 37.5 33.6 32.8 21.0 19.8 16.3 15.3
MeMe
OH
AcO
OBn
32
13C NMR (101 MHz, CDCl3)
f1 (ppm)

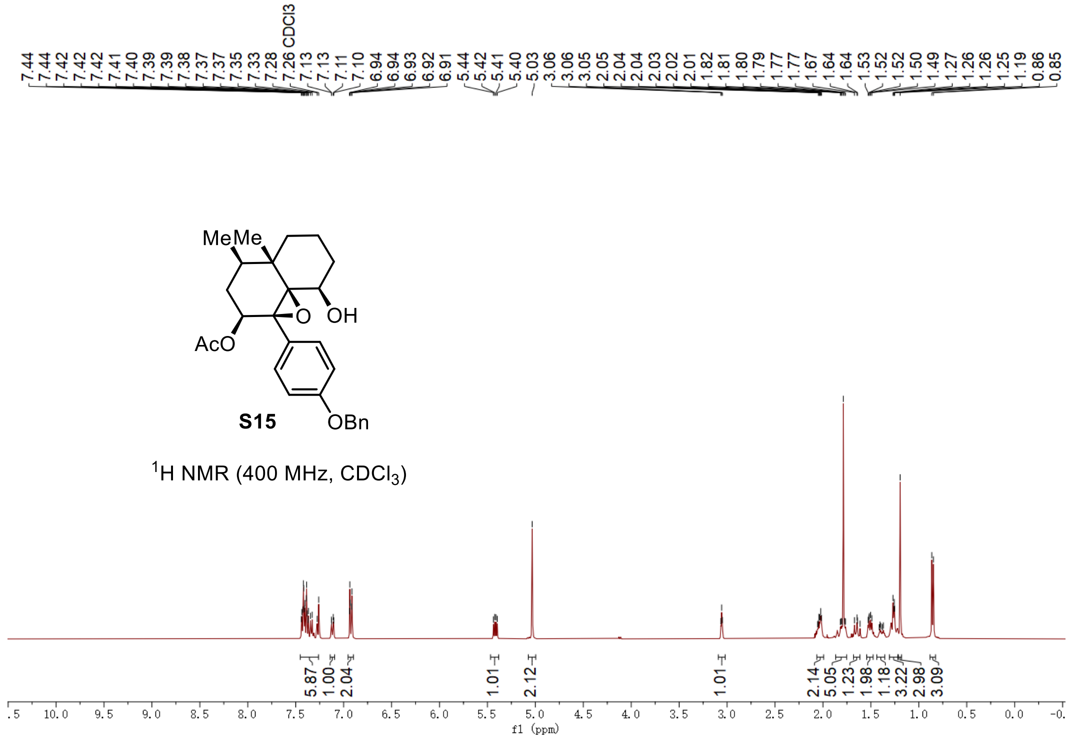
7.44 7.44 7.42 7.42 7.41 7.40 7.39 7.39 7.38 7.37 7.37 7.35 7.33 7.28 7.26 CDCl3 7.13 7.13 7.11 7.10 6.94 6.94 6.93 6.92 6.91 5.44 5.42 5.41 5.40 5.03 3.06 3.05 2.05 2.04 2.04 2.03 2.02 2.01 1.82 1.81 1.80 1.79 1.77 1.77 1.67 1.64 1.64 1.53 1.52 1.52 1.50 1.49 1.27 1.26 1.26 1.25 1.19 0.86 0.85
MeMe
O
OH
AcO
OBn
S15
^{1}H NMR (400 MHz, CDCl$_3$)
5.87 1.00 2.04 1.01 2.12 1.01 2.14 5.05 1.23 1.98 1.18 3.22 2.98 3.09
f1 (ppm)

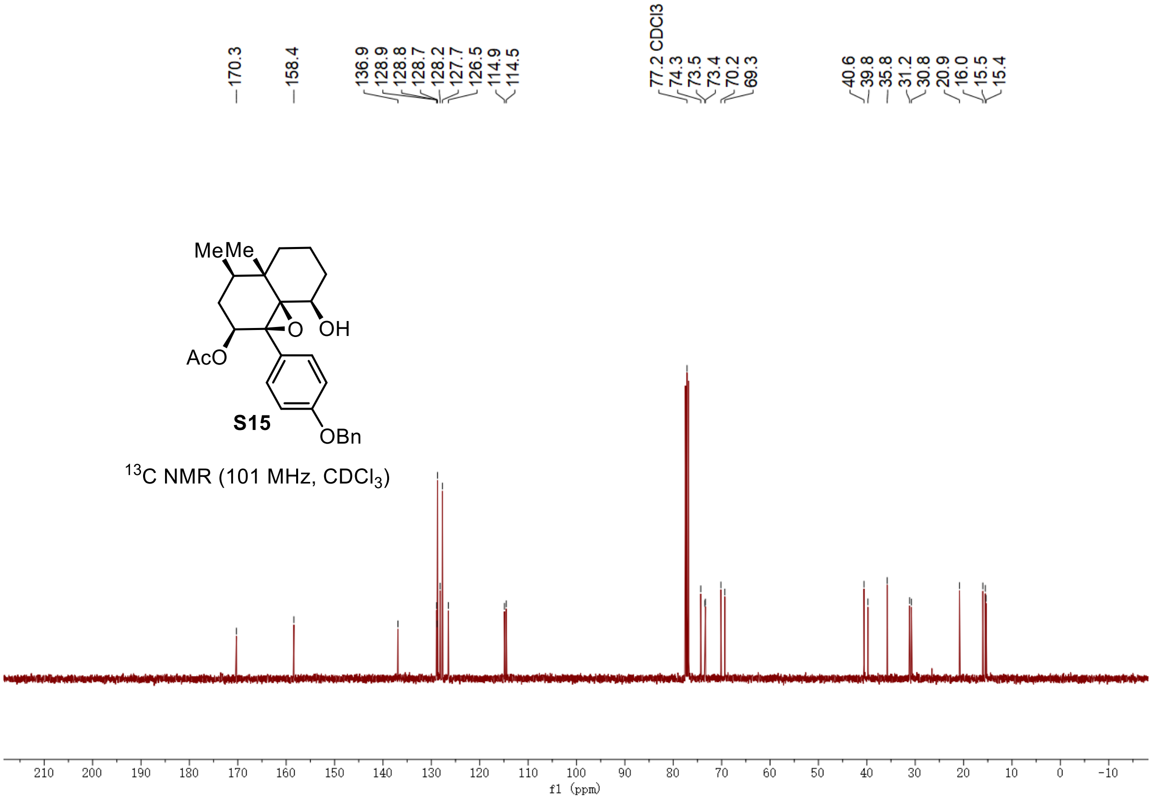
170.3 158.4 136.9 128.9 128.8 128.7 128.2 127.7 126.5 114.9 114.5 77.2 CDCl3 74.3 73.5 73.4 70.2 69.3 40.6 39.8 35.8 31.2 30.8 20.9 16.0 15.5 15.4
MeMe
O
OH
AcO
OBn
S15
^{13}C NMR (101 MHz, CDCl$_3$)
f1 (ppm)

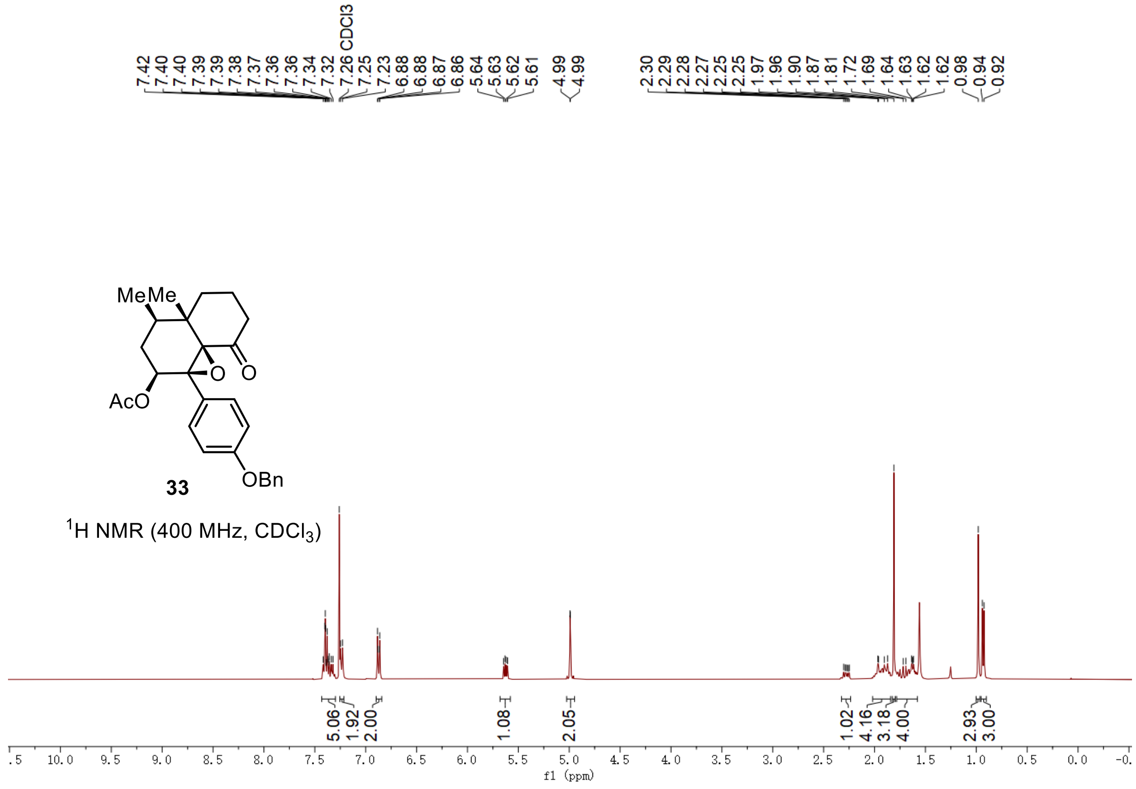
7.42 7.40 7.40 7.39 7.39 7.38 7.37 7.36 7.36 7.34 7.32 7.26 CDCl3 7.25 7.23 6.88 6.88 6.87 6.86 5.64 5.63 5.62 5.61 4.99 4.99
2.30 2.29 2.28 2.27 2.25 2.25 1.97 1.96 1.90 1.87 1.81 1.72 1.69 1.64 1.63 1.62 1.62 0.98 0.94 0.92
MeMe
O
O
AcO
OBn
33
^{1}H NMR (400 MHz, $CDCl_3$)
5.06 1.92 2.00 1.08 2.05 1.02 4.16 3.18 4.00 2.93 3.00
f1 (ppm)

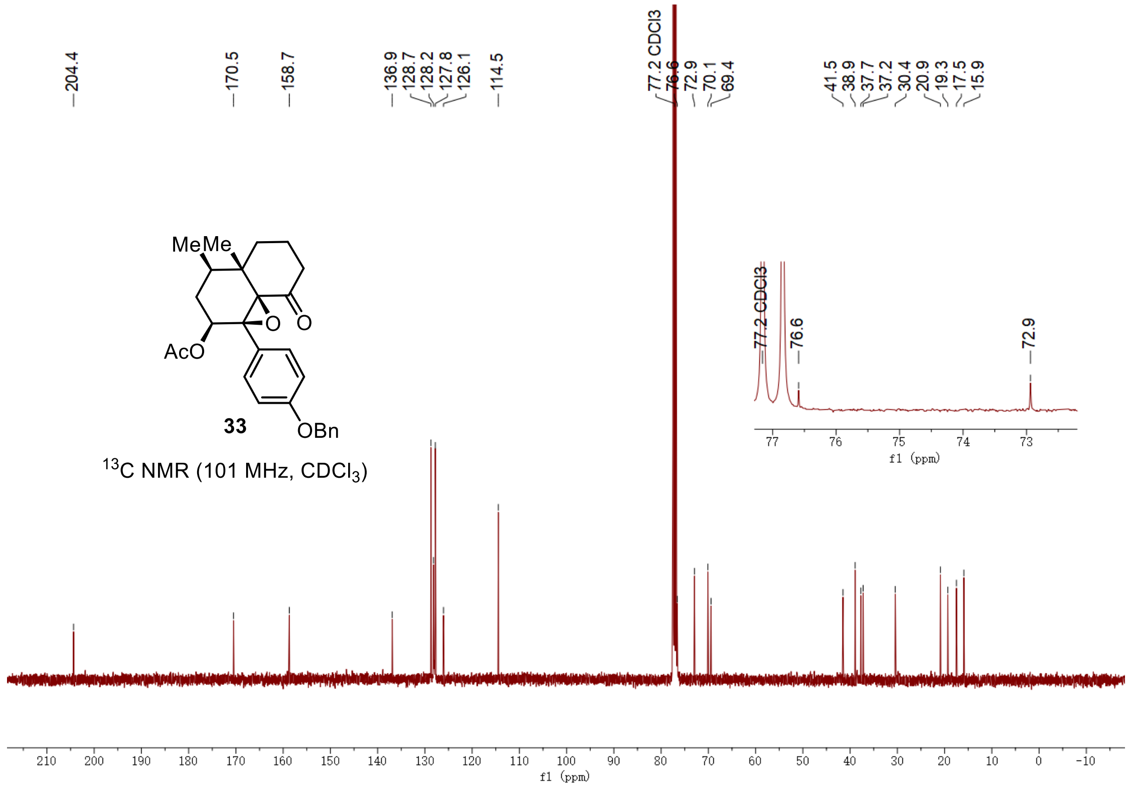
204.4 170.5 158.7 136.9 128.7 128.2 127.8 126.1 114.5 77.2 CDCl3 76.6 72.9 70.1 69.4 41.5 38.9 37.7 37.2 30.4 20.9 19.3 17.5 15.9
MeMe
O
O
AcO
OBn
33
^{13}C NMR (101 MHz, $CDCl_3$)
77.2 CDCl3
76.6
72.9
f1 (ppm)
f1 (ppm)

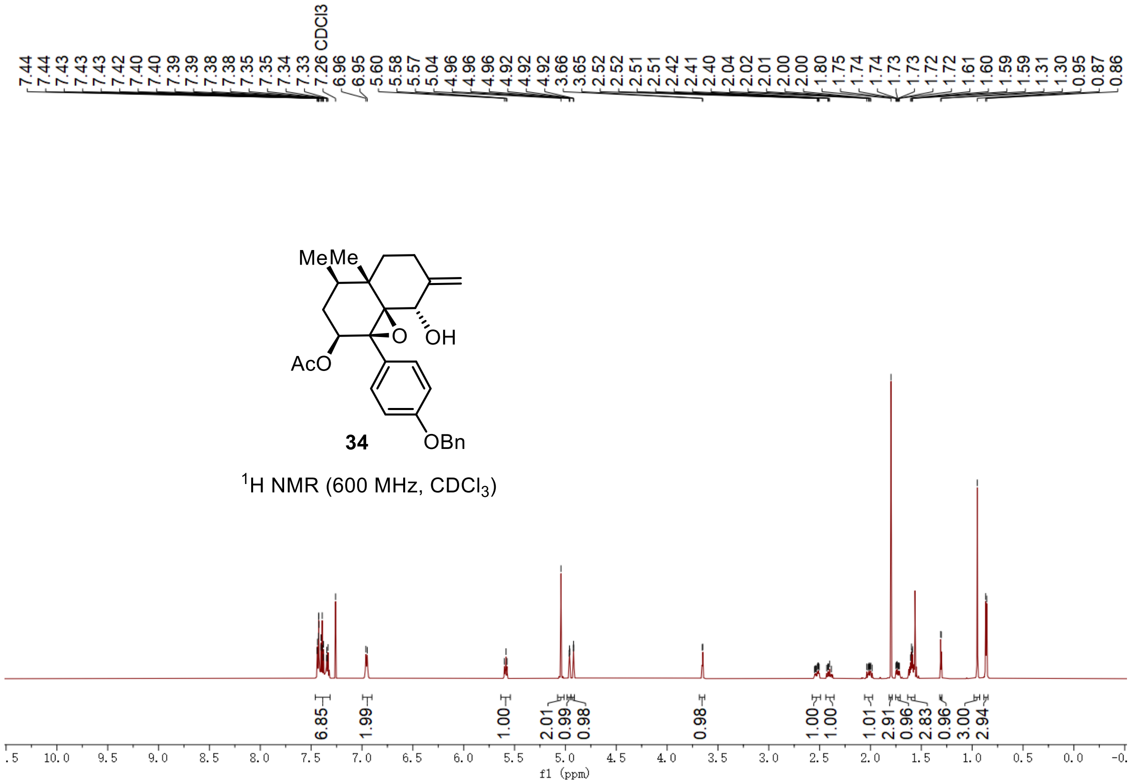
7.44 7.44 7.43 7.43 7.43 7.42 7.40 7.40 7.39 7.39 7.38 7.38 7.35 7.35 7.34 7.33 7.26 CDCl3 6.96 6.95 5.60 5.58 5.57 5.04 4.96 4.96 4.96 4.92 4.92 4.92 3.66 3.65 2.52 2.52 2.51 2.51 2.42 2.41 2.40 2.04 2.02 2.01 2.00 2.00 1.80 1.75 1.74 1.74 1.73 1.73 1.72 1.72 1.61 1.60 1.59 1.59 1.31 1.30 0.95 0.87 0.86
MeMe
O
OH
AcO
OBn
34
^{1}H NMR (600 MHz, $CDCl_3$)
6.85 1.99 1.00 2.01 0.99 0.98 0.98 1.00 1.00 1.01 2.91 0.96 2.83 0.96 3.00 2.94
f1 (ppm)

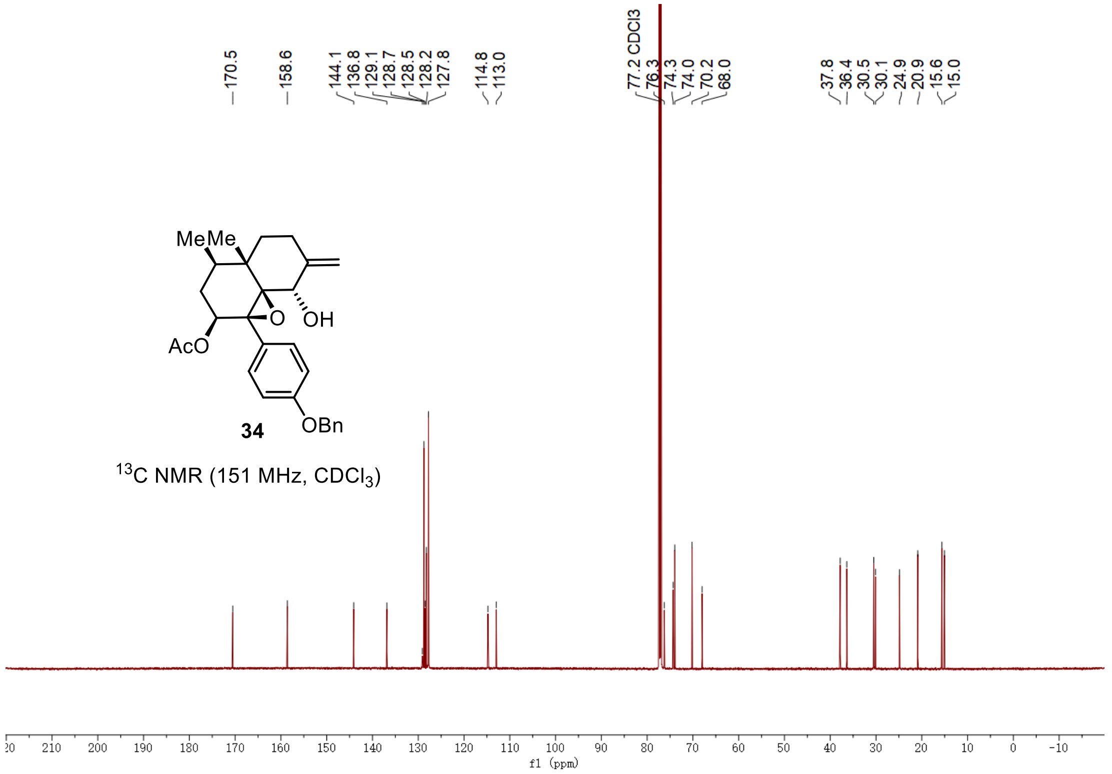
170.5 158.6 144.1 136.8 129.1 128.7 128.5 128.2 127.8 114.8 113.0 77.2 CDCl3 76.3 74.3 74.0 70.2 68.0 37.8 36.4 30.5 30.1 24.9 20.9 15.6 15.0
MeMe
O
OH
AcO
OBn
34
^{13}C NMR (151 MHz, $CDCl_3$)
f1 (ppm)

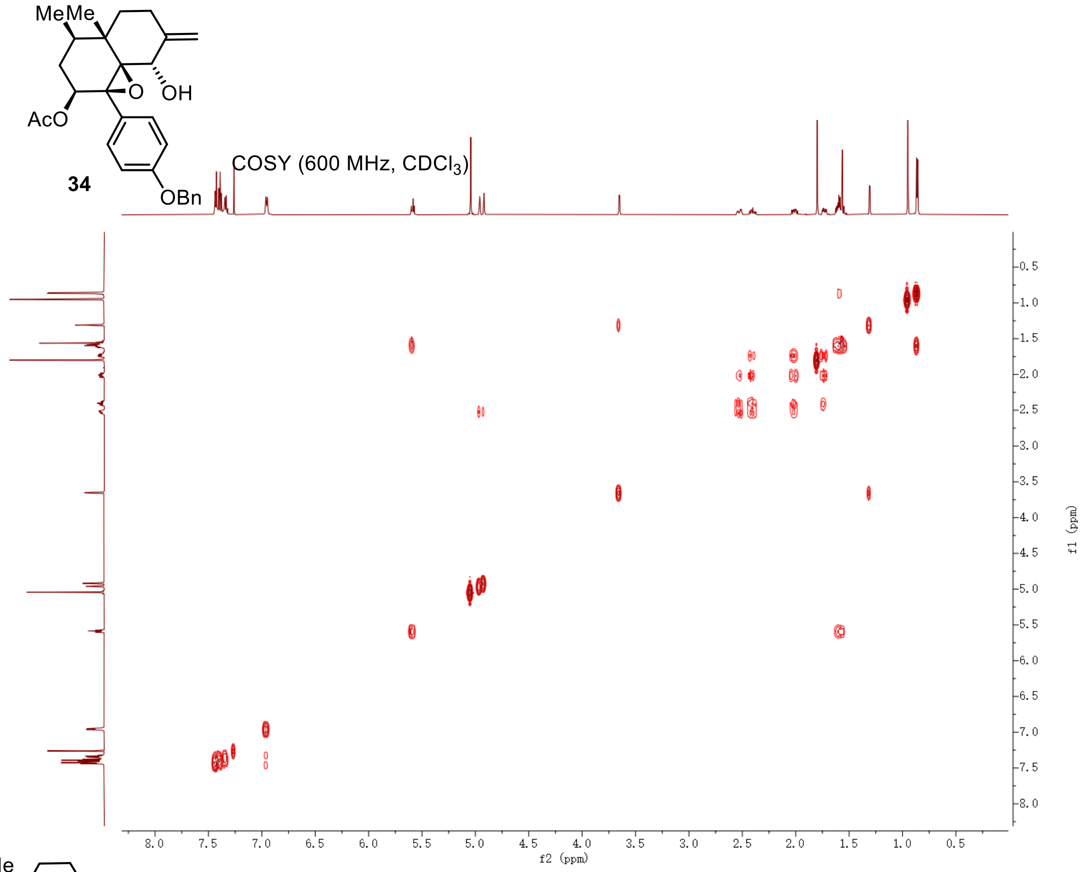

MeMe
OH
O
AcO
34
OBn
COSY (600 MHz, CDCl3)
f2 (ppm)
f1 (ppm)

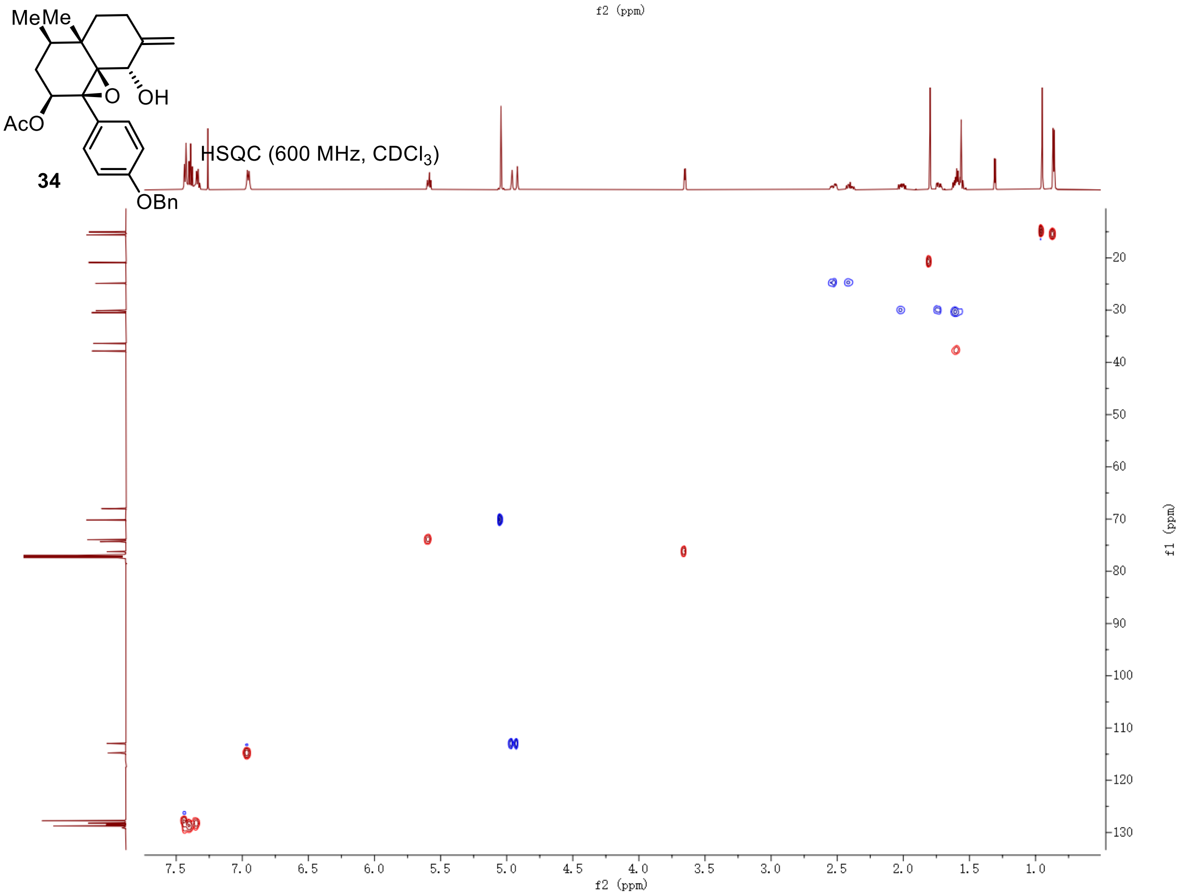

MeMe
OH
O
AcO
34
OBn
HSQC (600 MHz, CDCl3)
f2 (ppm)
f1 (ppm)

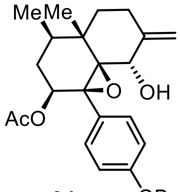

34

HMBC (600 MHz, $CDCl_3$)

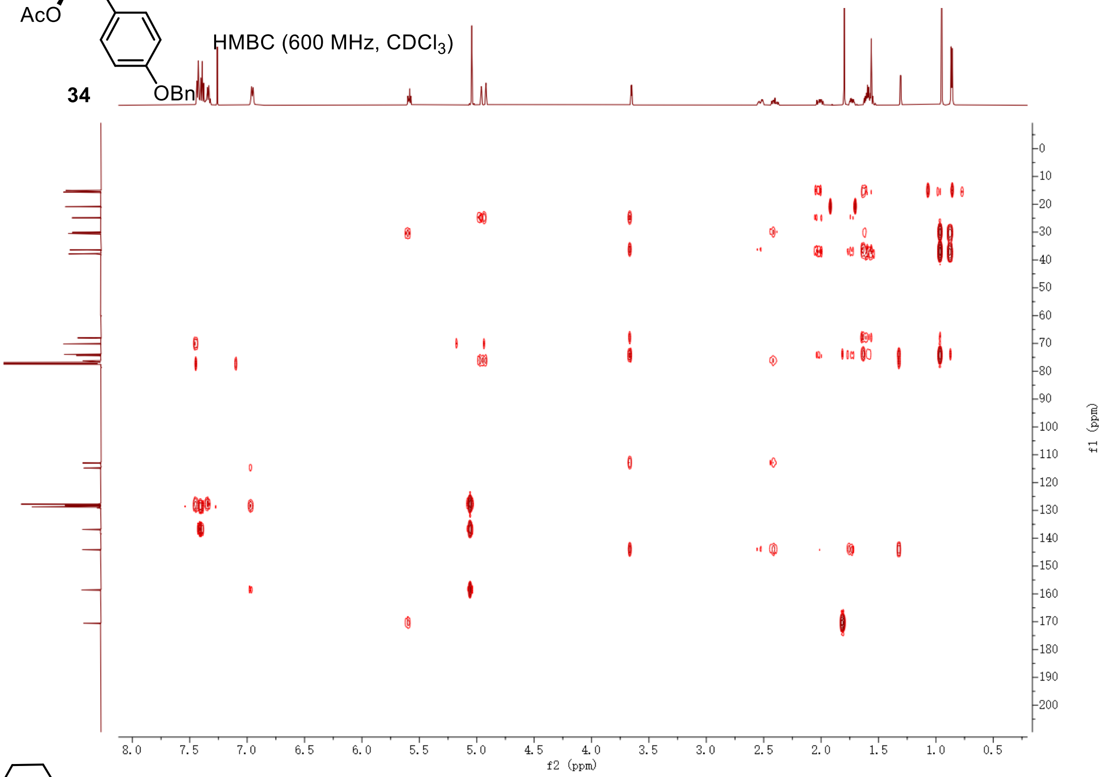

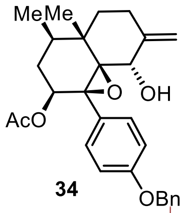

34

NOESY (600 MHz, $CDCl_3$)

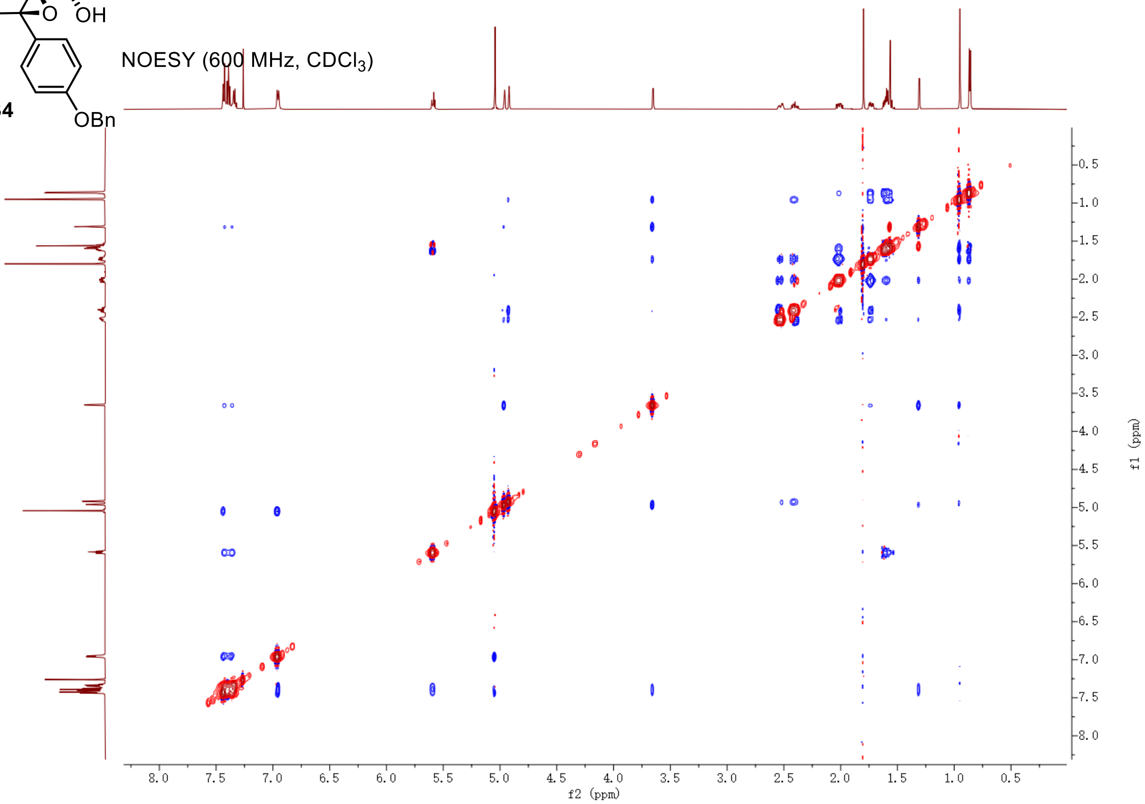

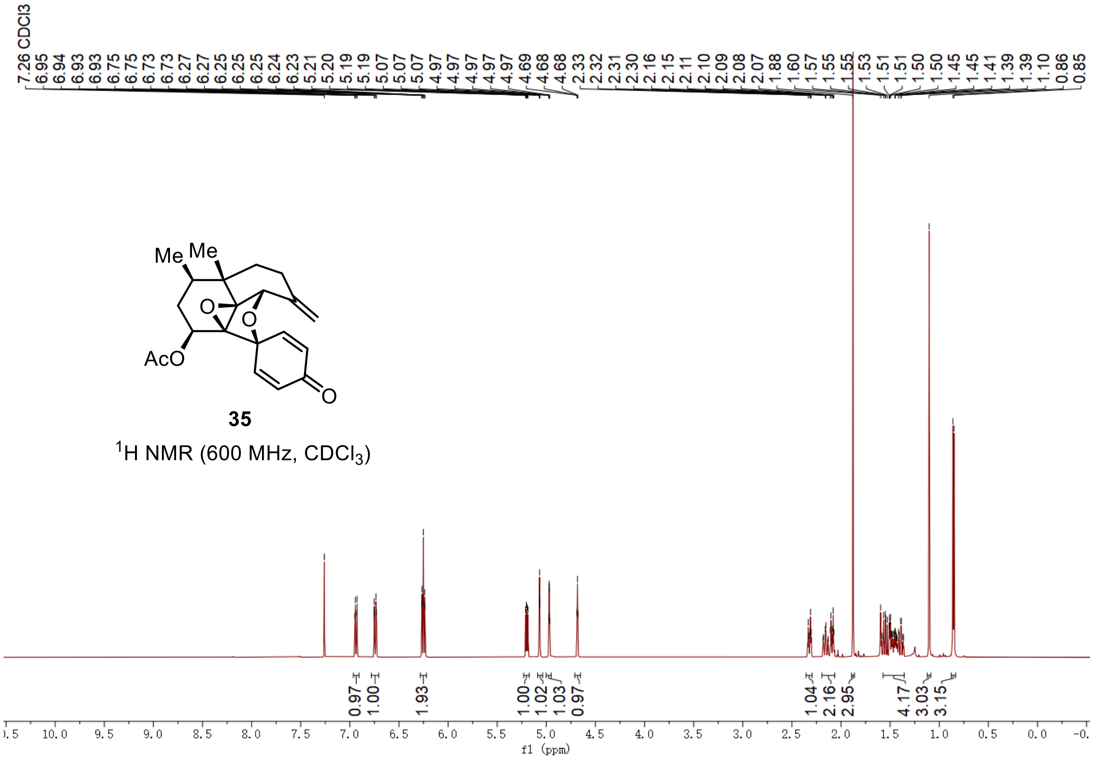

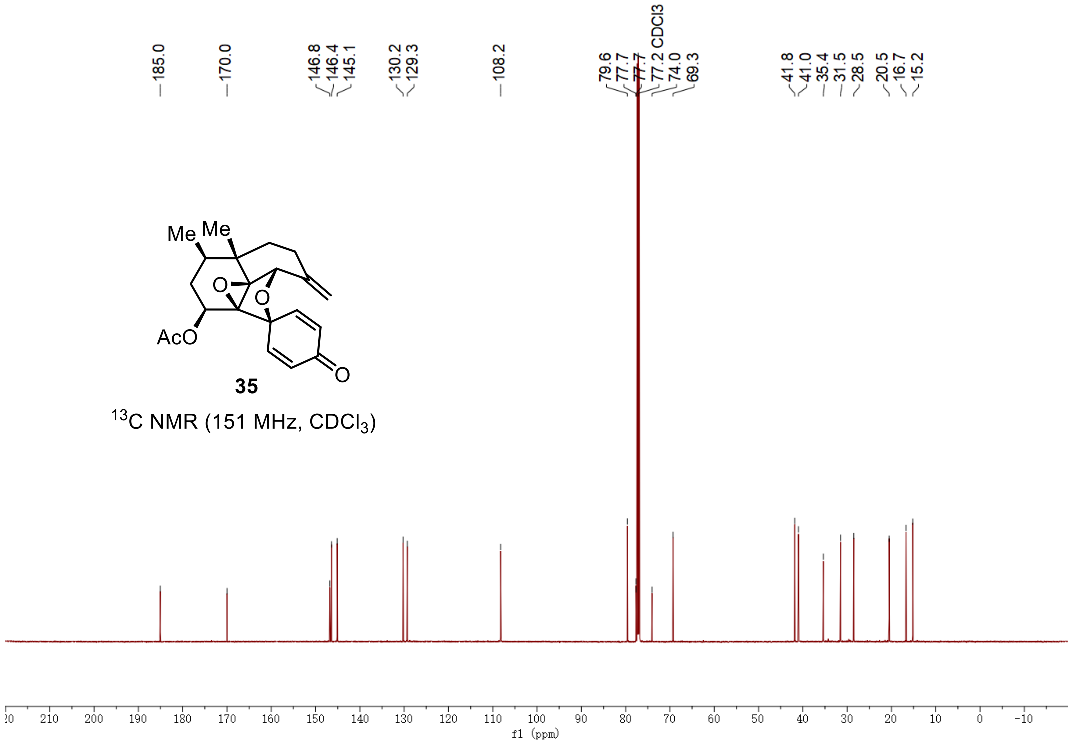

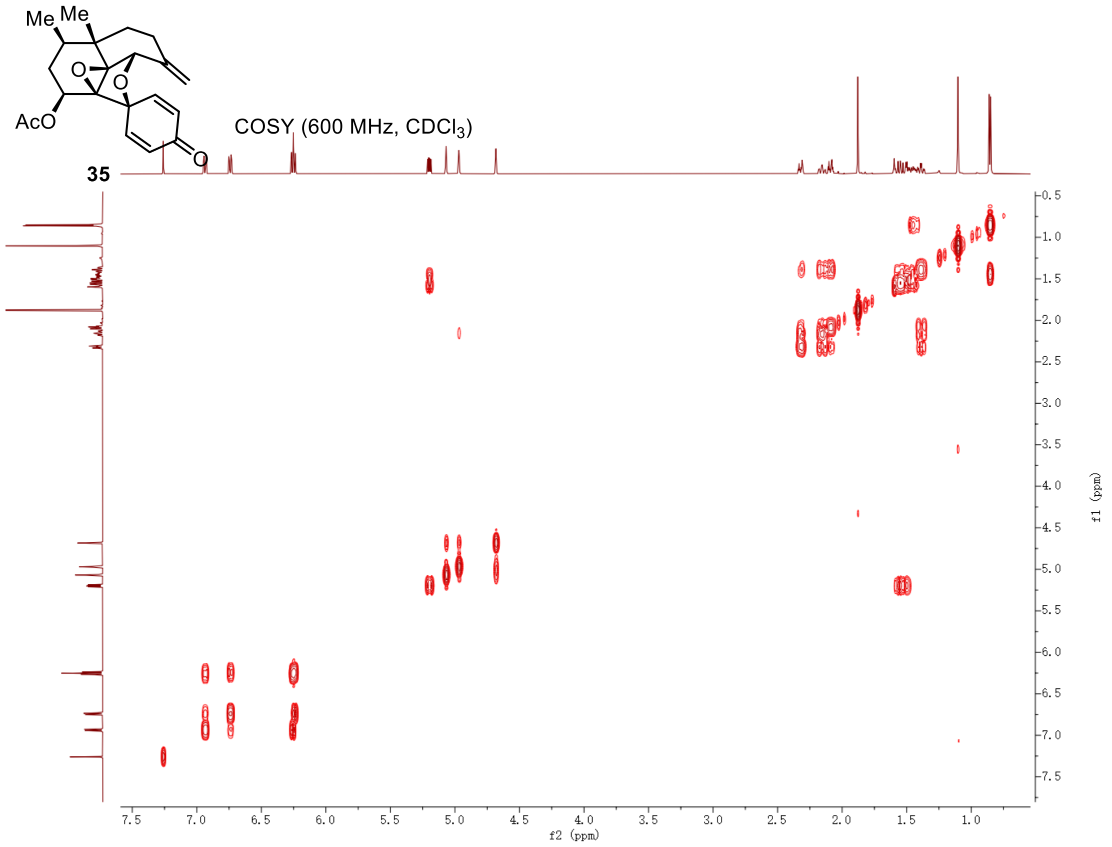

Me Me
AcO
35
COSY (600 MHz, CDCl3)
f2 (ppm)
f1 (ppm)

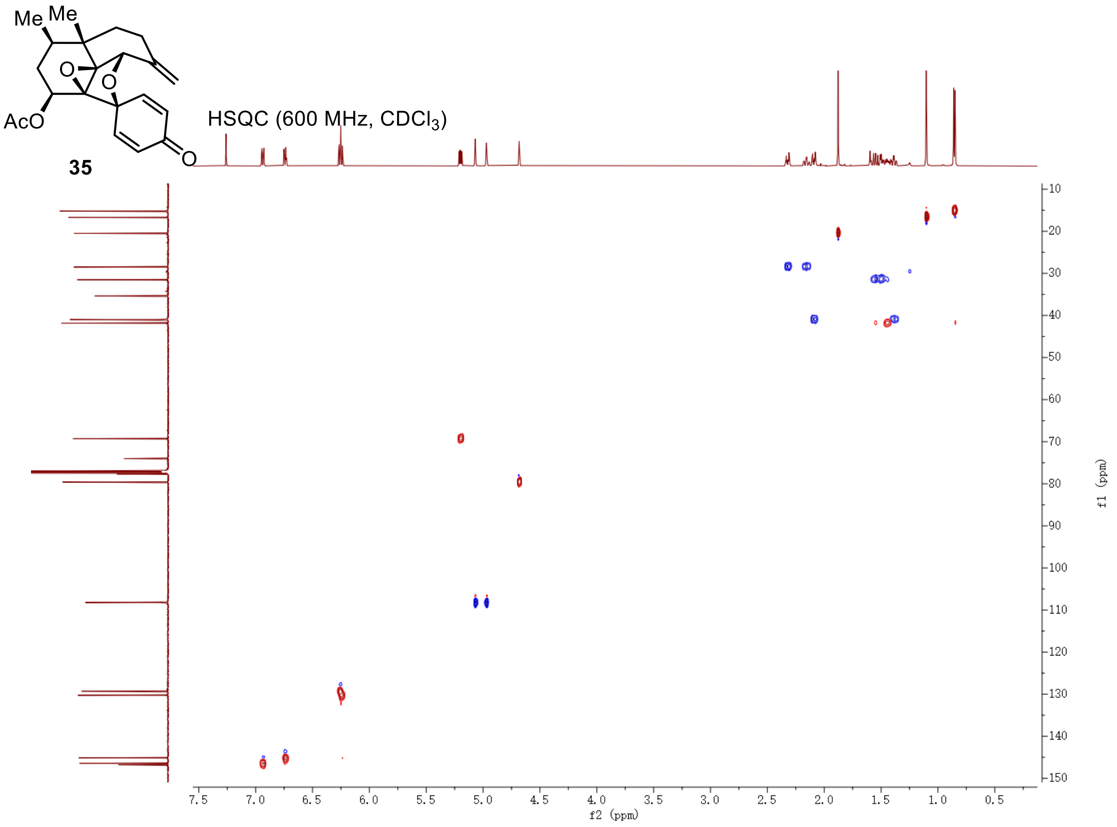

Me Me
AcO
35
HSQC (600 MHz, CDCl3)
f2 (ppm)
f1 (ppm)

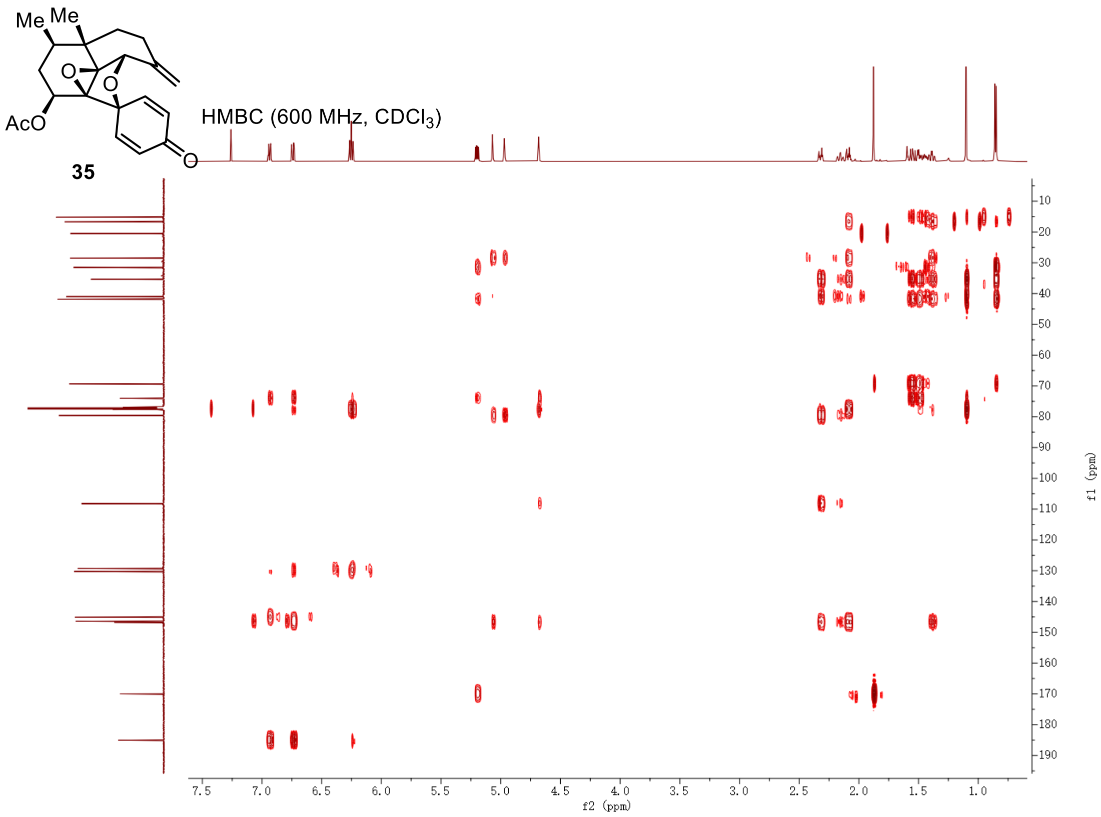
Me Me
O
O
AcO
O
35
HMBC (600 MHz, CDCl3)
f2 (ppm)
f1 (ppm)

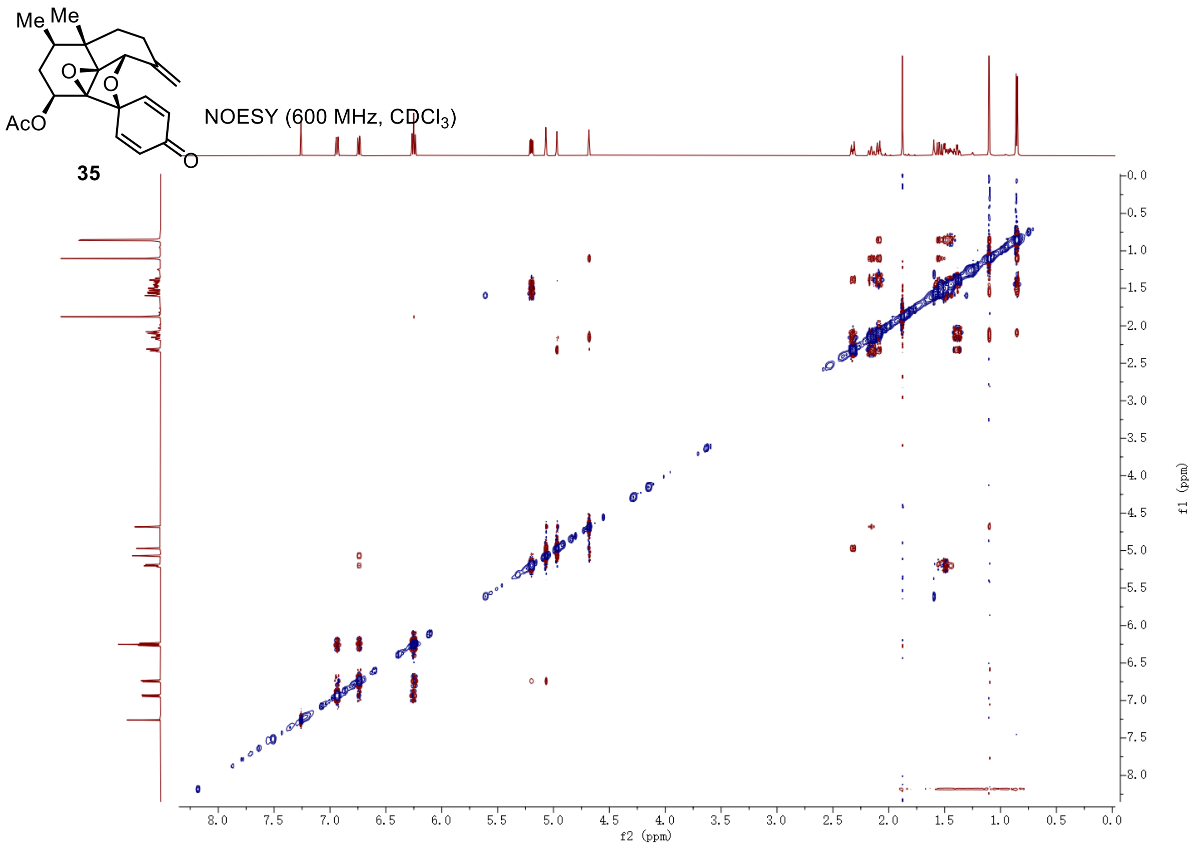
Me Me
O
O
AcO
O
35
NOESY (600 MHz, CDCl3)
f2 (ppm)
f1 (ppm)

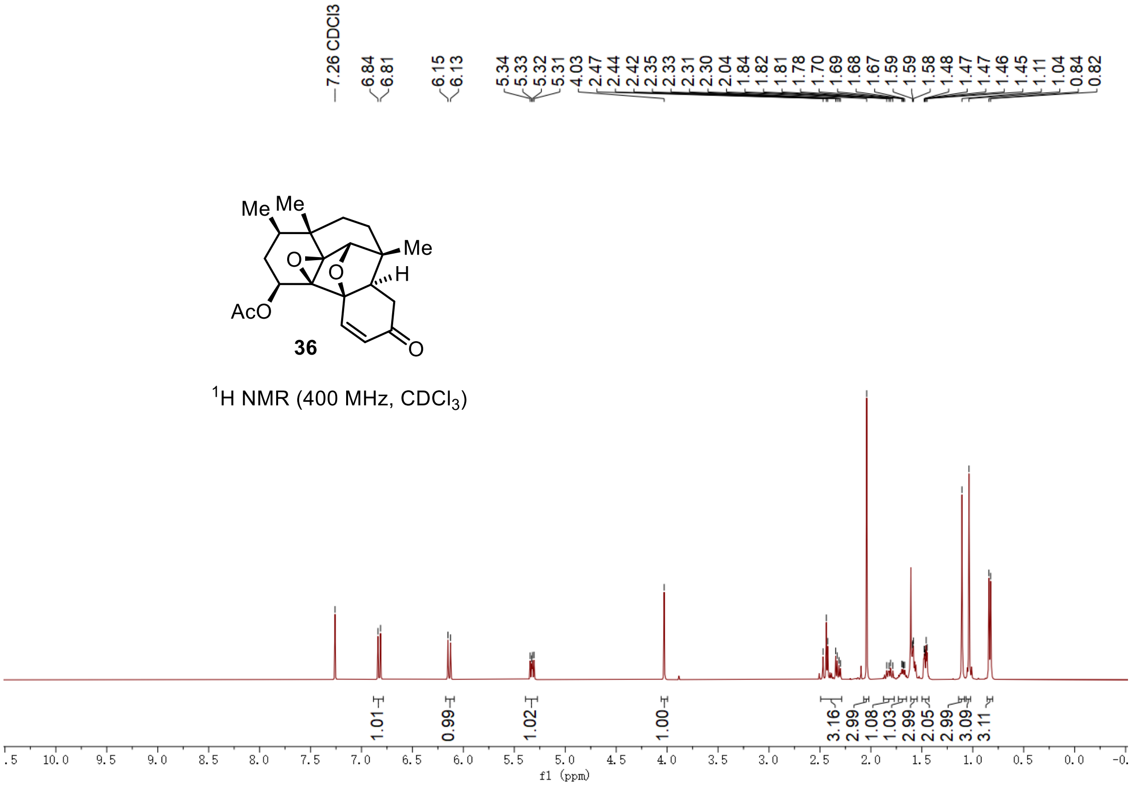

^{1}H NMR (400 MHz, $CDCl_3$)

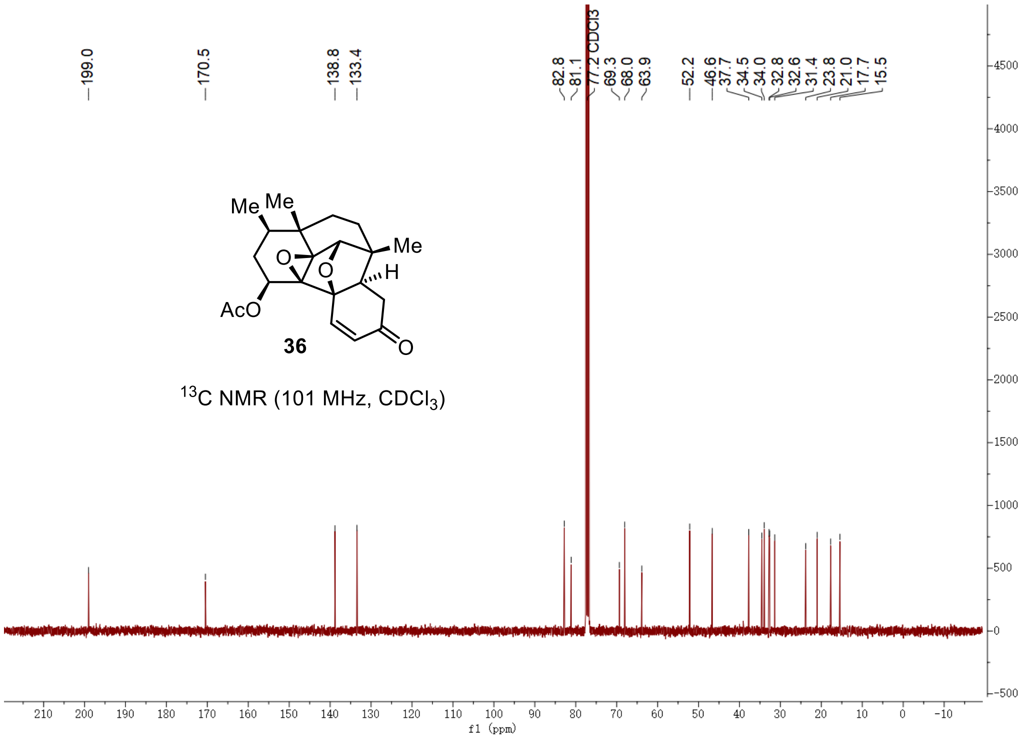

^{13}C NMR (101 MHz, $CDCl_3$)

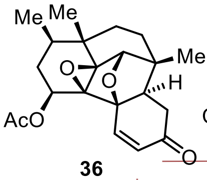
Me
Me
Me
H
O
O
AcO
O

36

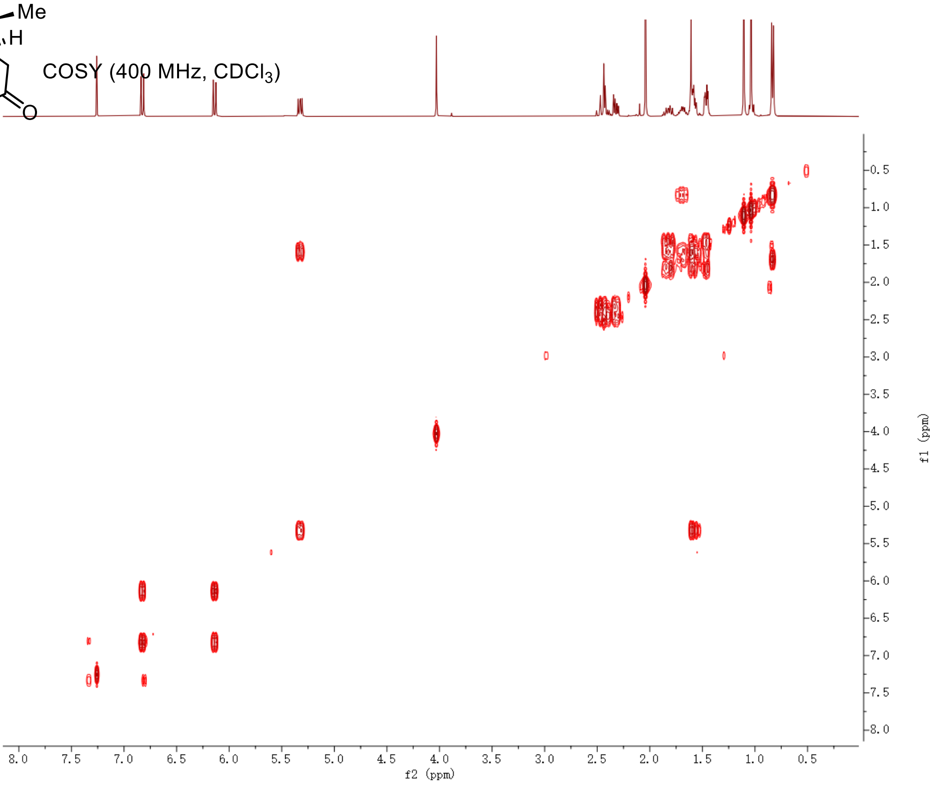
COSY (400 MHz, $CDCl_3$)
f2 (ppm)
f1 (ppm)

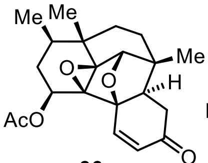
Me
Me
Me
H
O
O
AcO
O

36

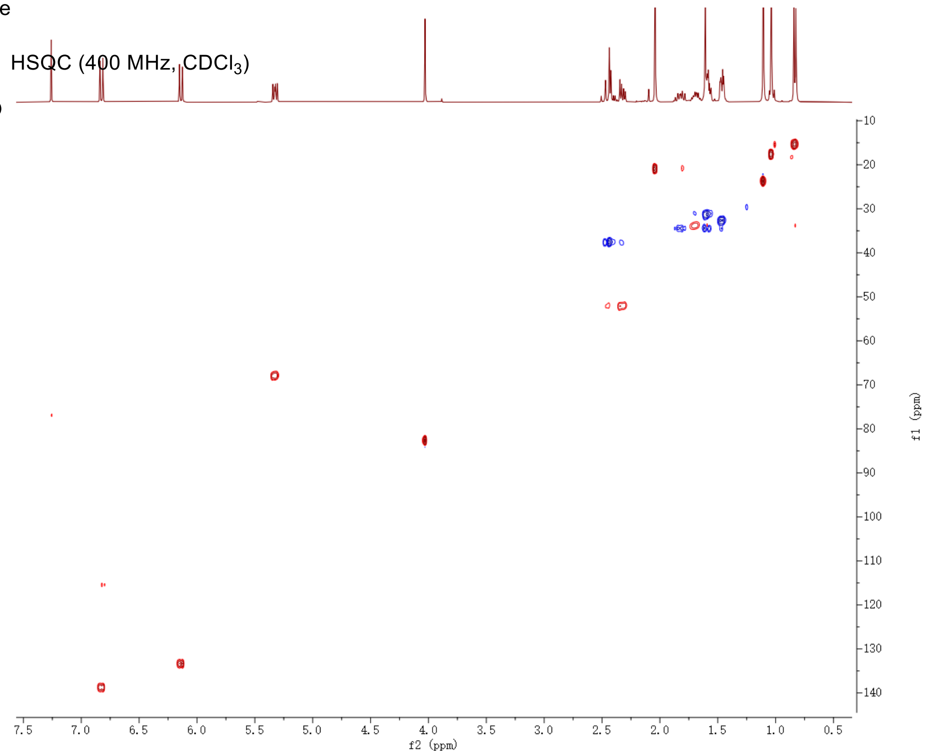
HSQC (400 MHz, $CDCl_3$)
f2 (ppm)
f1 (ppm)

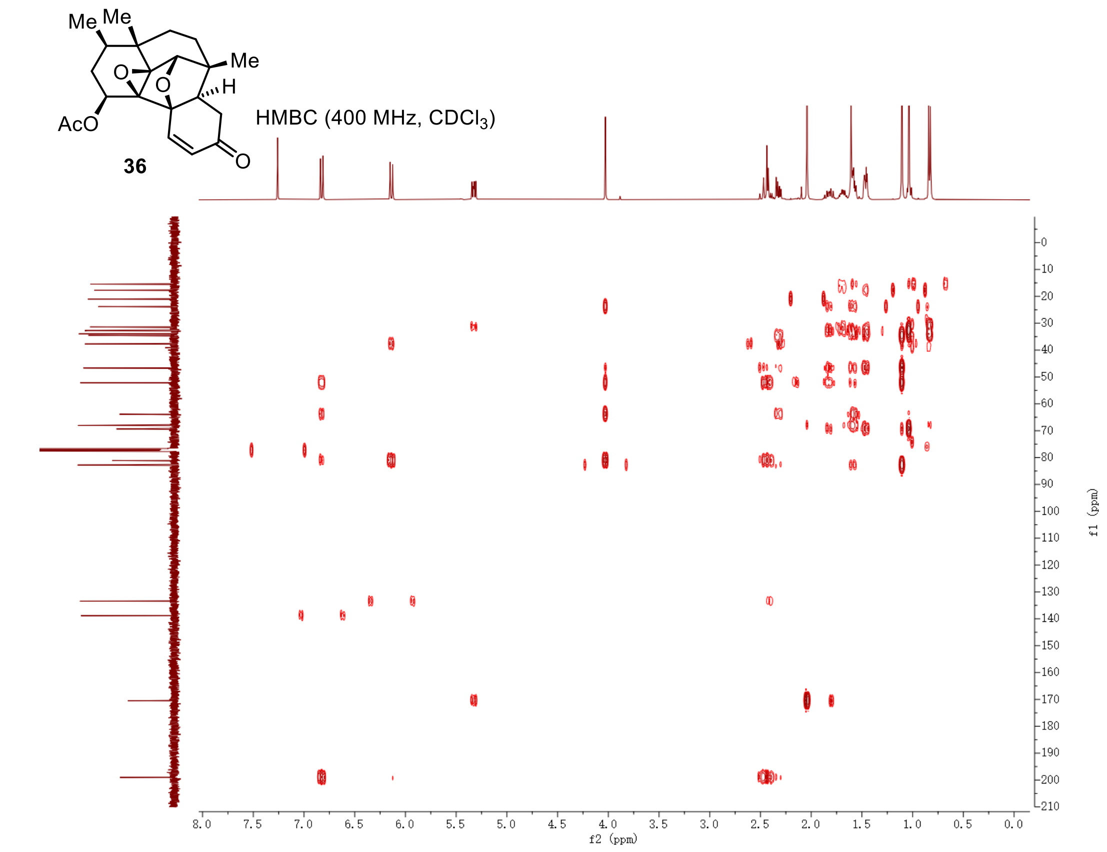
Me
Me
Me
H
O
O
AcO
O
36
HMBC (400 MHz, CDCl3)
f2 (ppm)
f1 (ppm)

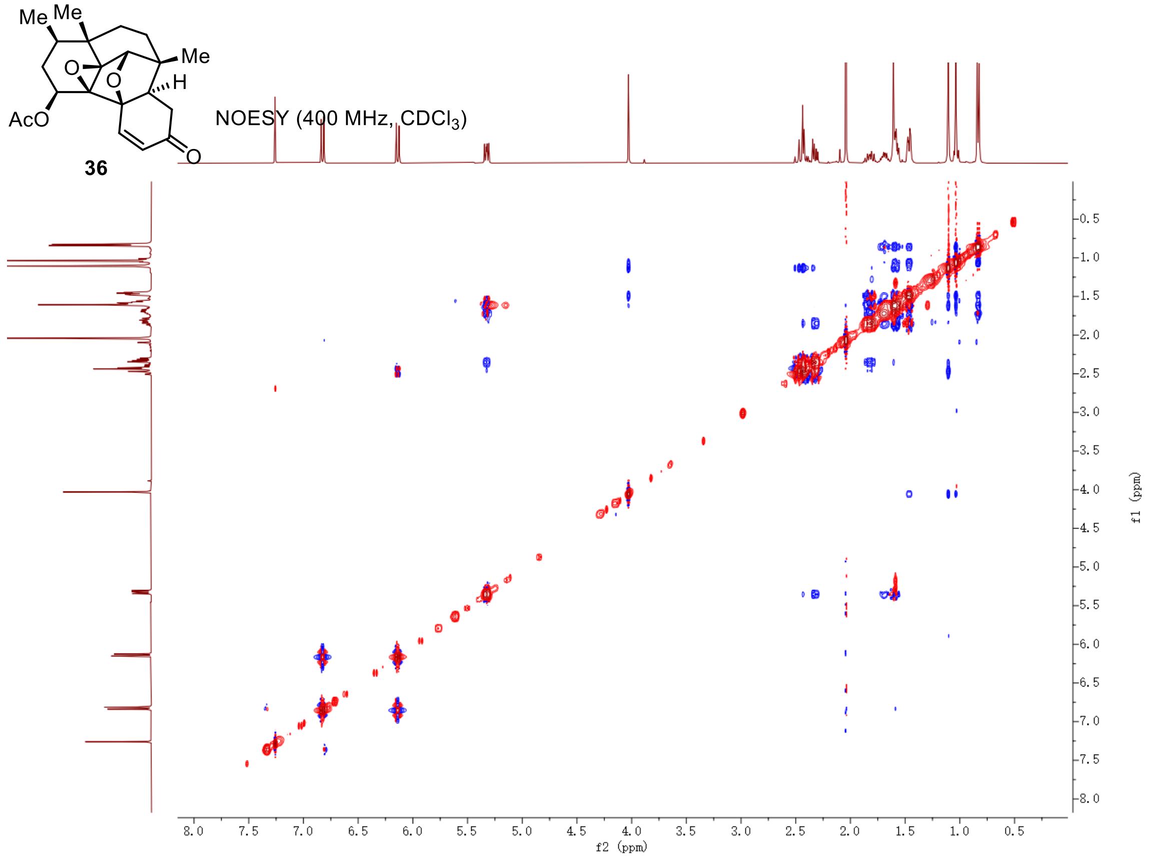
Me
Me
Me
H
O
O
AcO
O
36
NOESY (400 MHz, CDCl3)
f2 (ppm)
f1 (ppm)

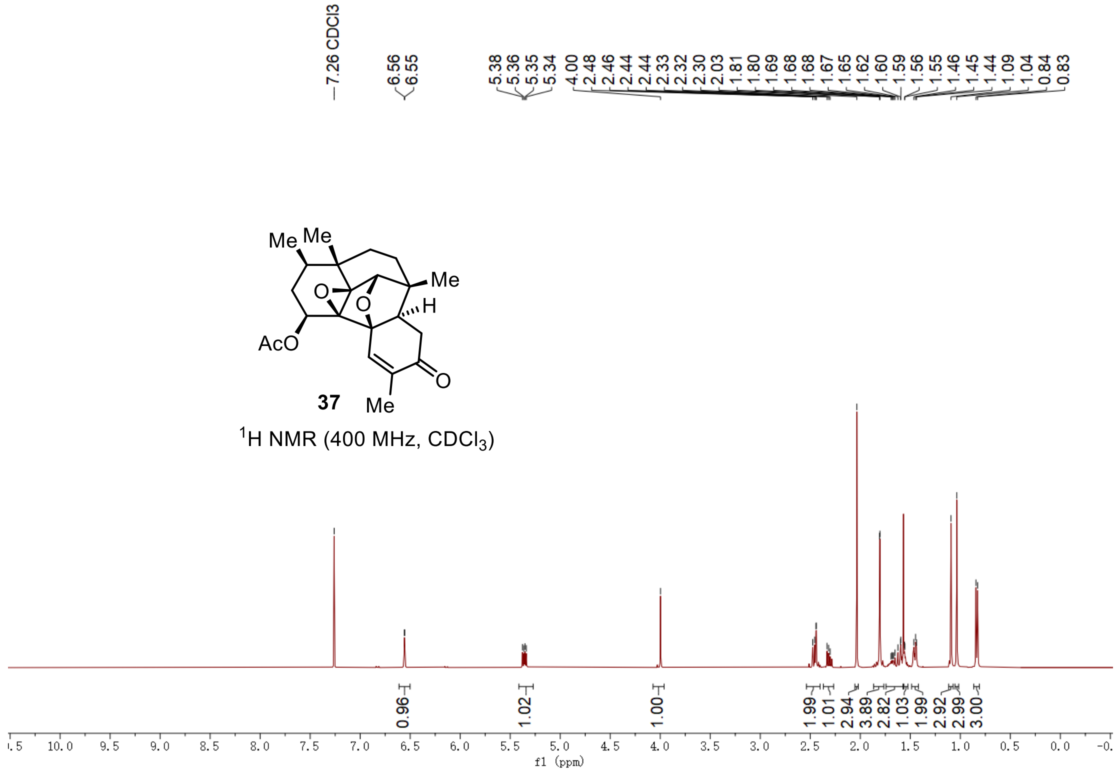
7.26 CDCl3
6.56
6.55
5.38
5.36
5.35
5.34
4.00
2.48
2.46
2.44
2.44
2.33
2.32
2.30
2.03
1.81
1.80
1.69
1.68
1.68
1.67
1.65
1.62
1.60
1.59
1.56
1.55
1.46
1.45
1.44
1.09
1.04
0.84
0.83
37
1H NMR (400 MHz, CDCl3)
0.96
1.02
1.00
1.99
1.01
2.94
3.89
2.82
1.03
1.99
2.92
2.99
3.00
f1 (ppm)

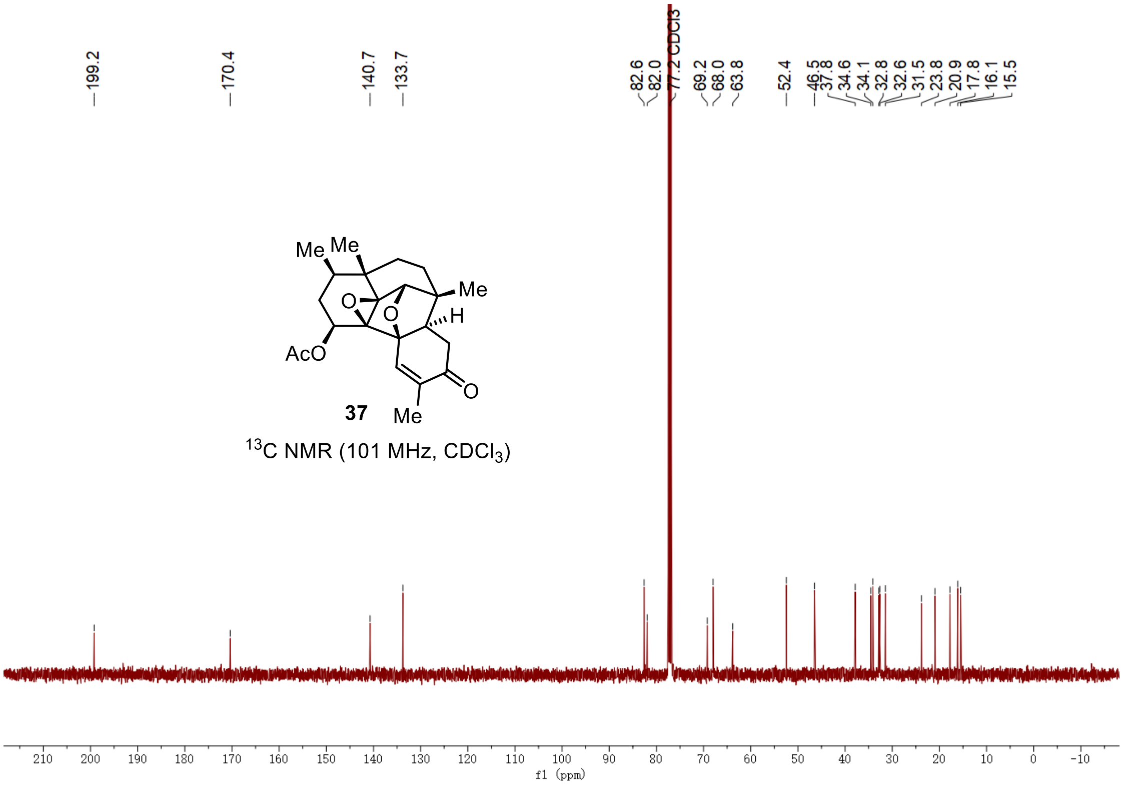
199.2
170.4
140.7
133.7
82.6
82.0
77.2 CDCl3
69.2
68.0
63.8
52.4
46.5
37.8
34.6
34.1
32.8
32.6
31.5
23.8
20.9
17.8
16.1
15.5
37
13C NMR (101 MHz, CDCl3)
f1 (ppm)

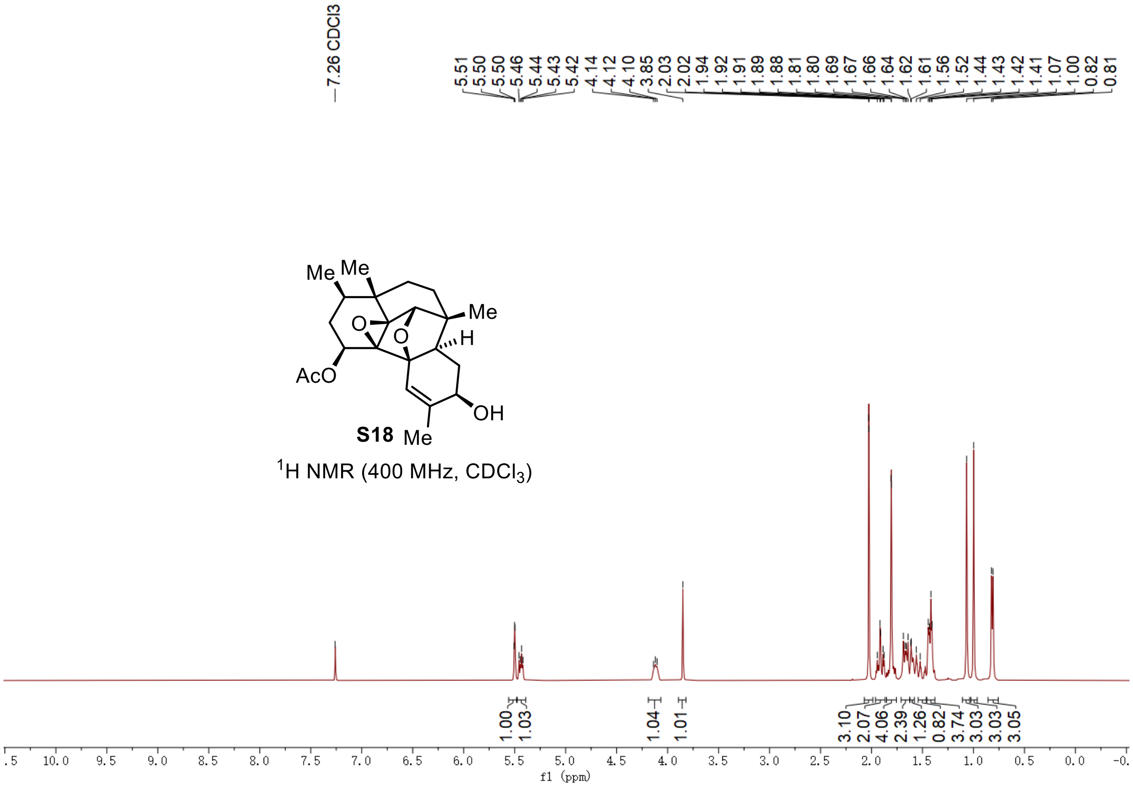
S18
^{1}H NMR (400 MHz, $CDCl_3$)

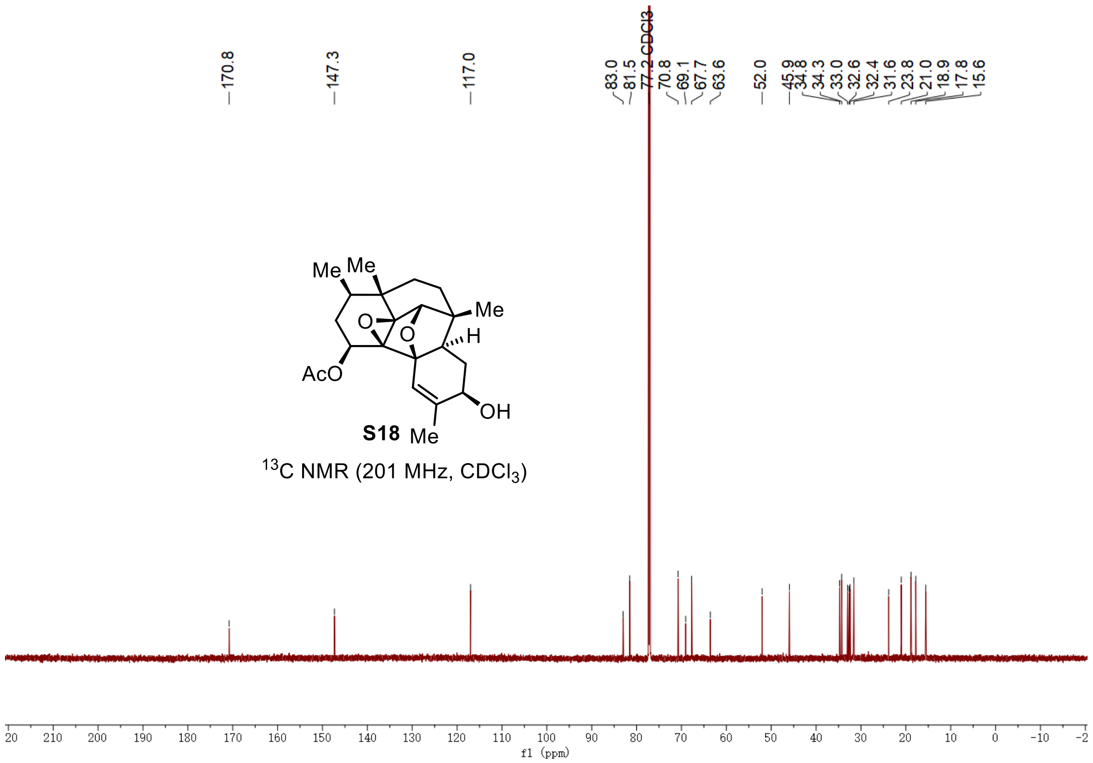
S18
^{13}C NMR (201 MHz, $CDCl_3$)

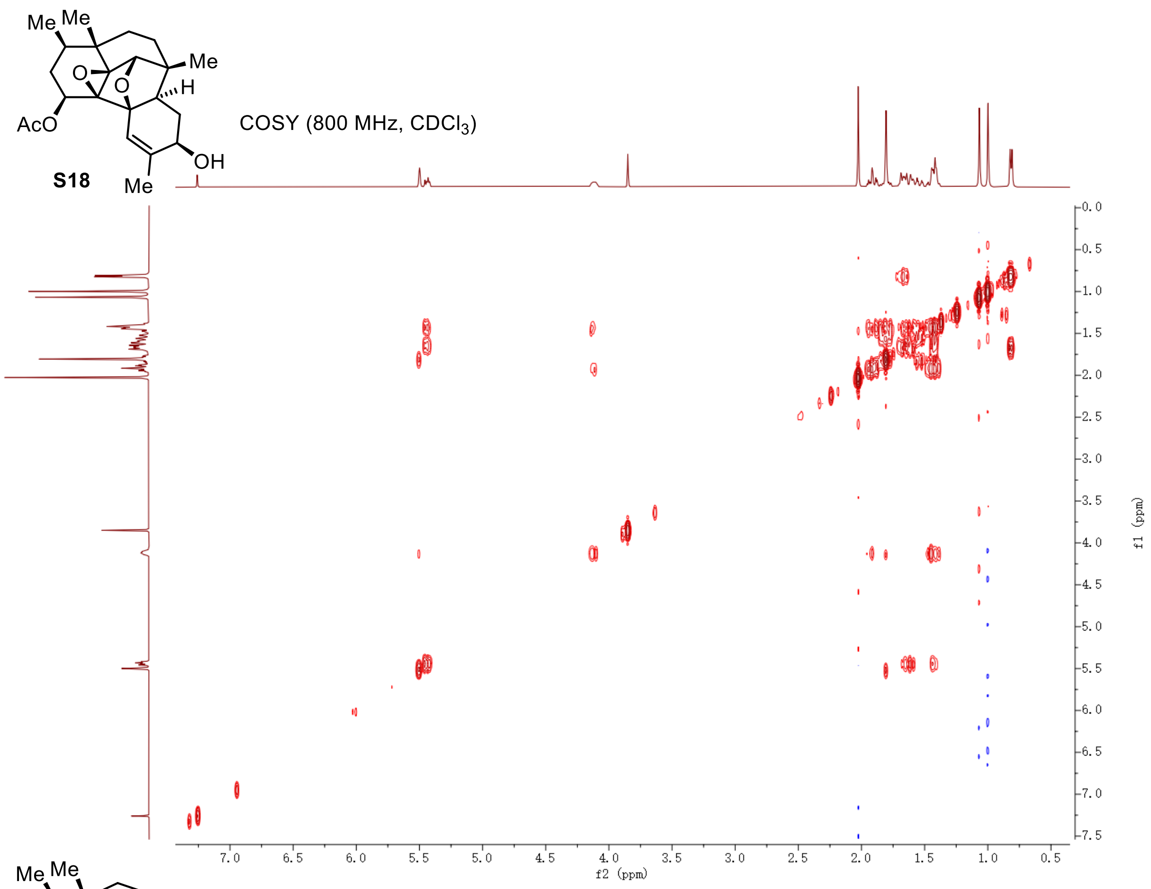

Me
Me
Me
O
O
H
AcO
OH
S18
Me
COSY (800 MHz, CDCl3)
f2 (ppm)
f1 (ppm)

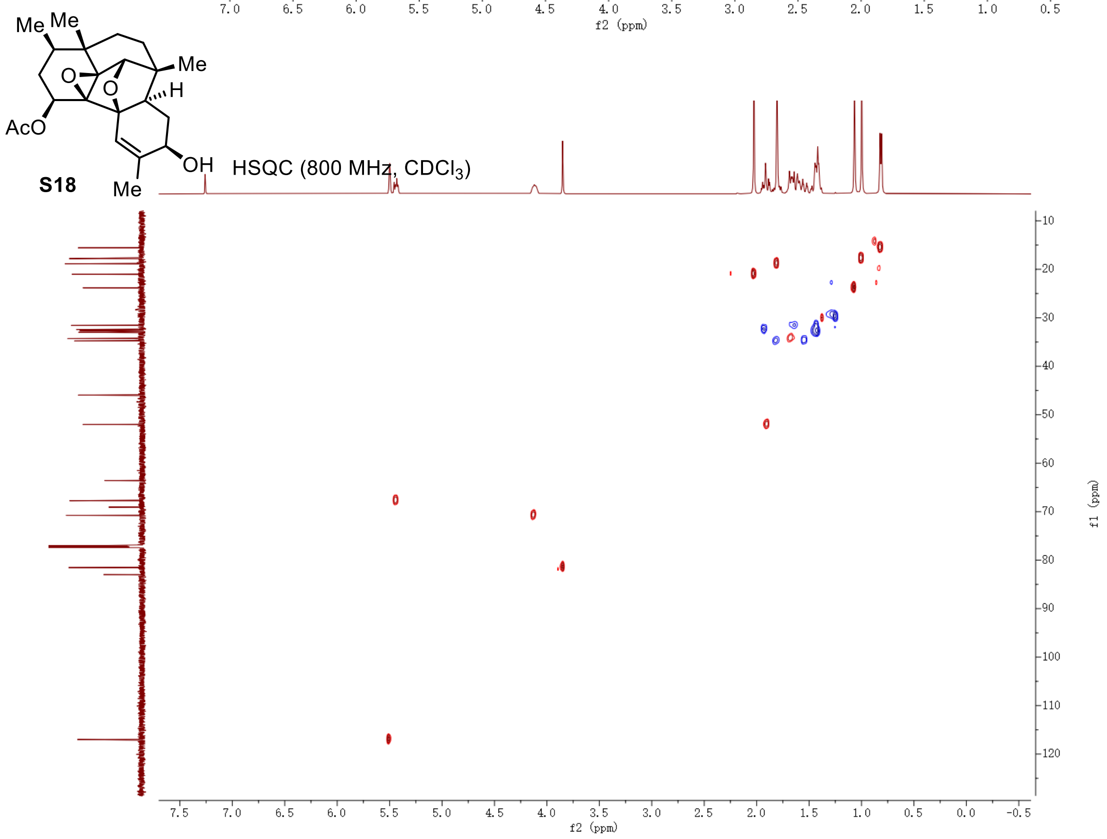

Me
Me
Me
O
O
H
AcO
OH
S18
Me
HSQC (800 MHz, CDCl3)
f2 (ppm)
f1 (ppm)

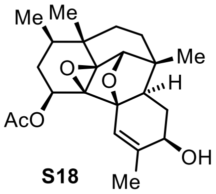

S18

HMBC (800 MHz, $CDCl_3$)

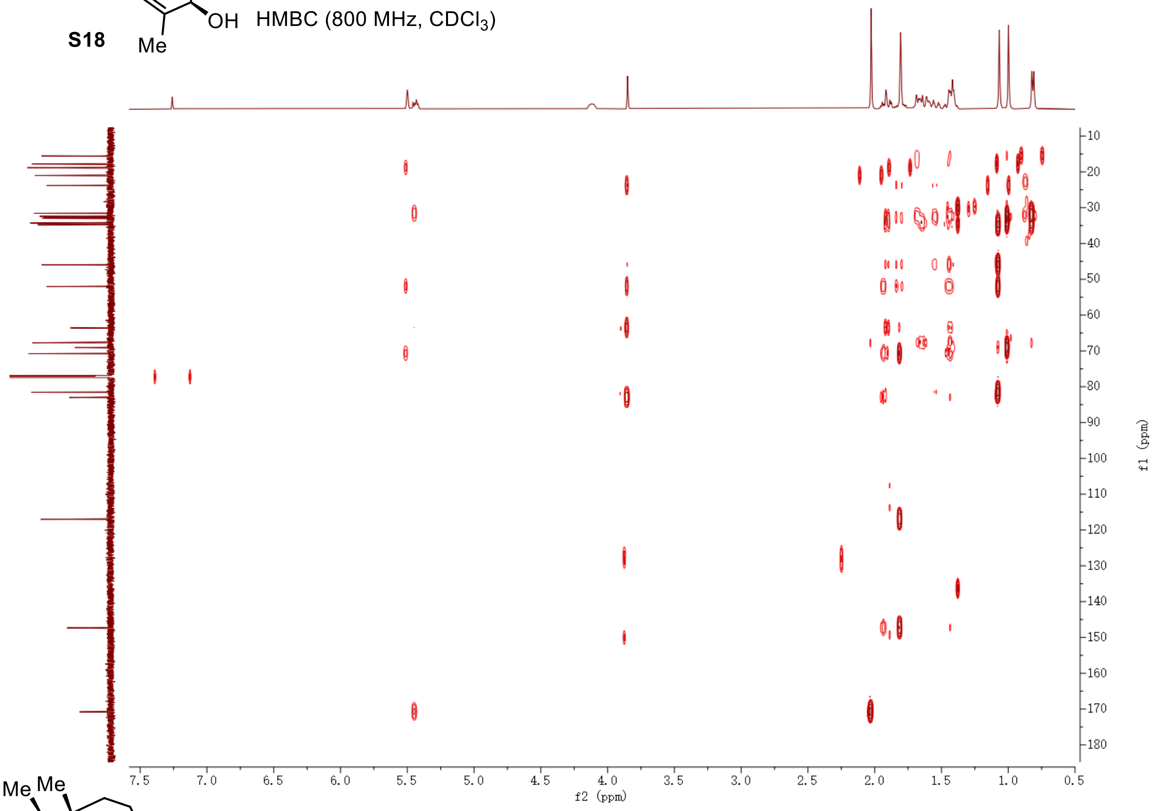

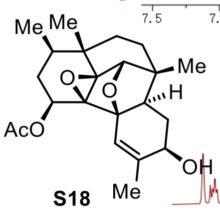

S18

NOESY (800 MHz, $CDCl_3$)

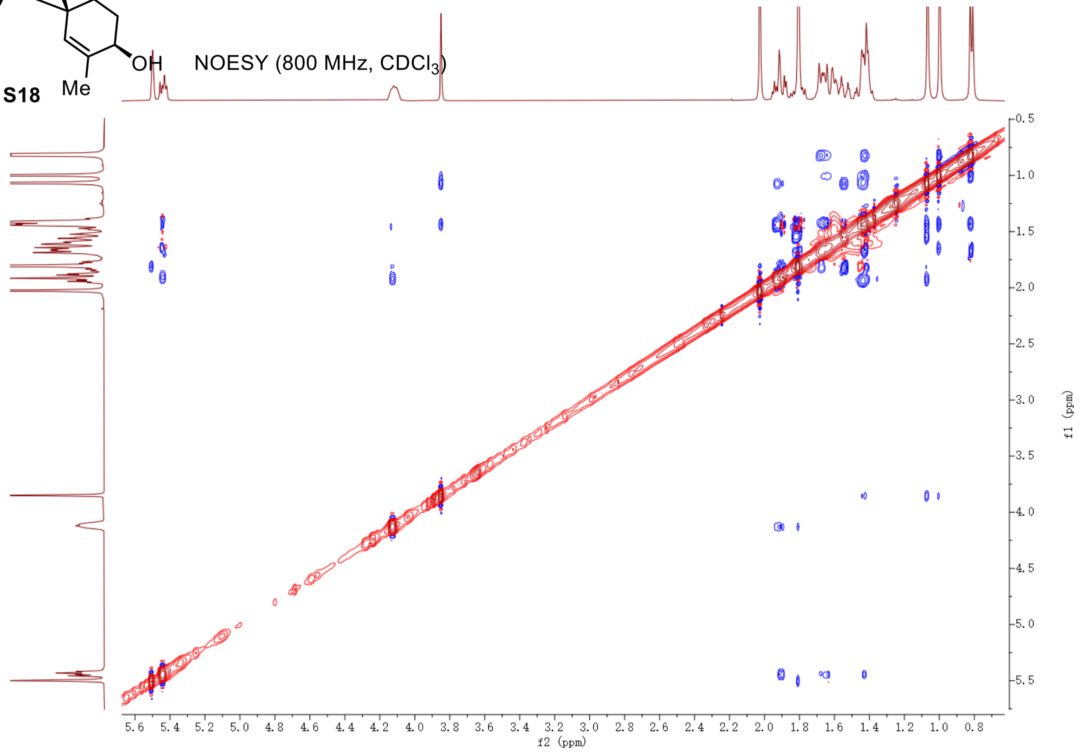

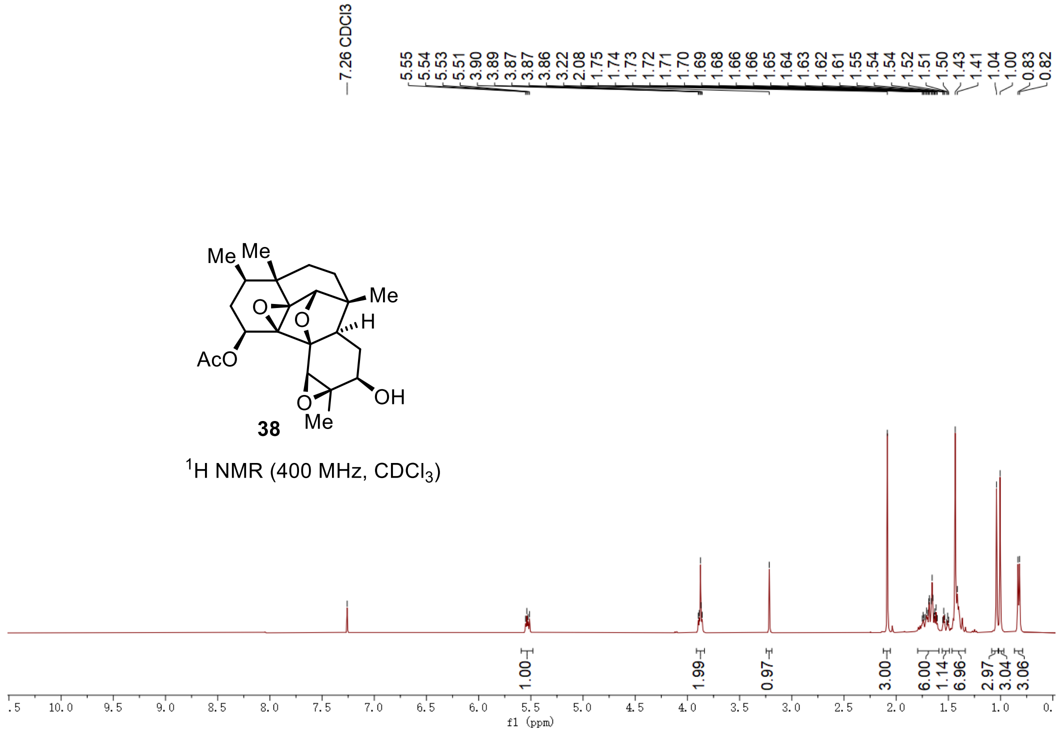

38

^{1}H NMR (400 MHz, $CDCl_3$)

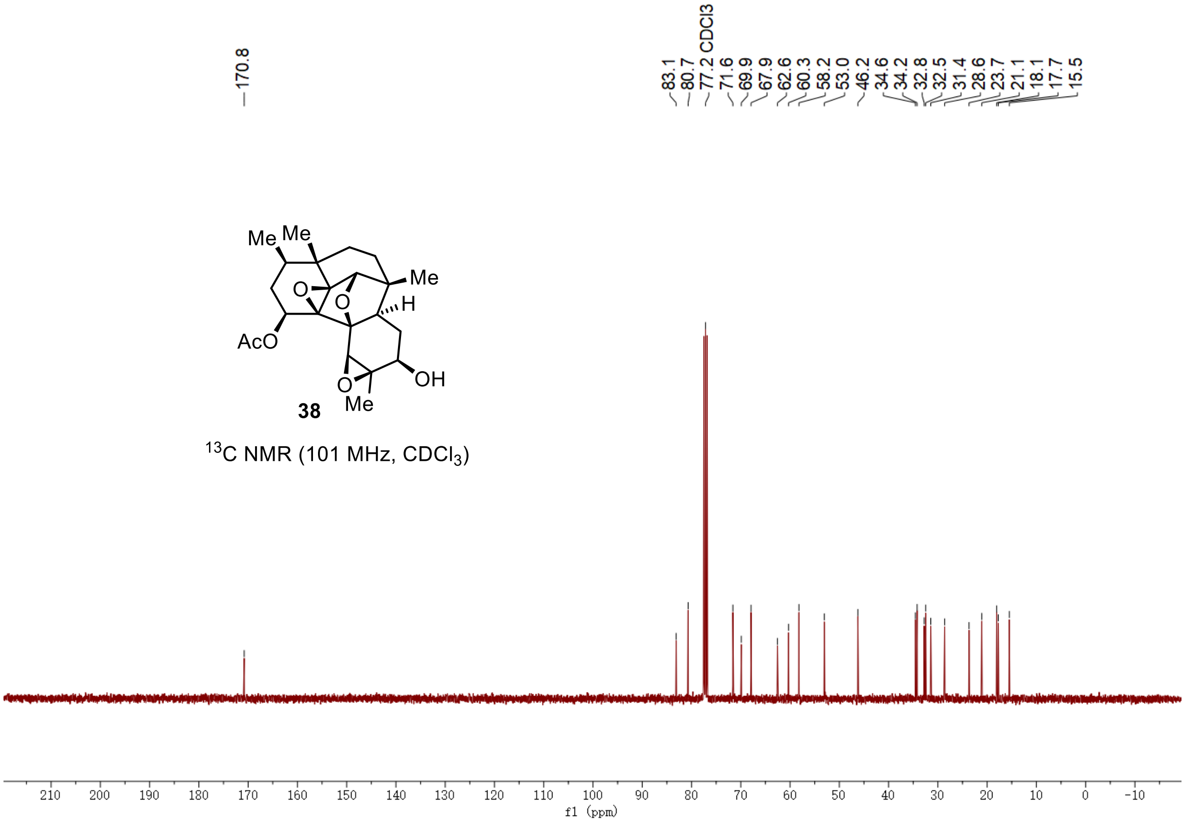

38

^{13}C NMR (101 MHz, $CDCl_3$)

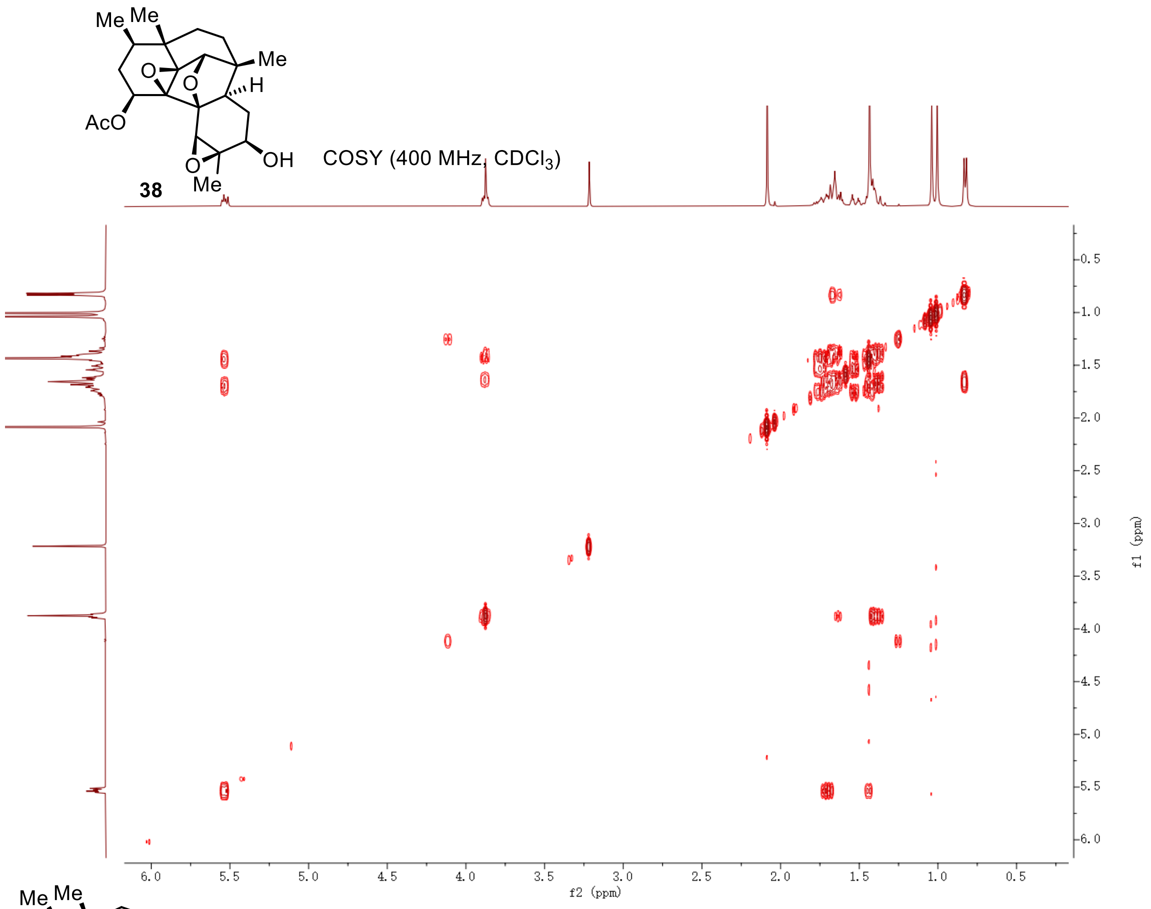
Me
Me
Me
H
O
O
AcO
O
OH
Me
38
COSY (400 MHz, CDCl3)
f2 (ppm)
f1 (ppm)

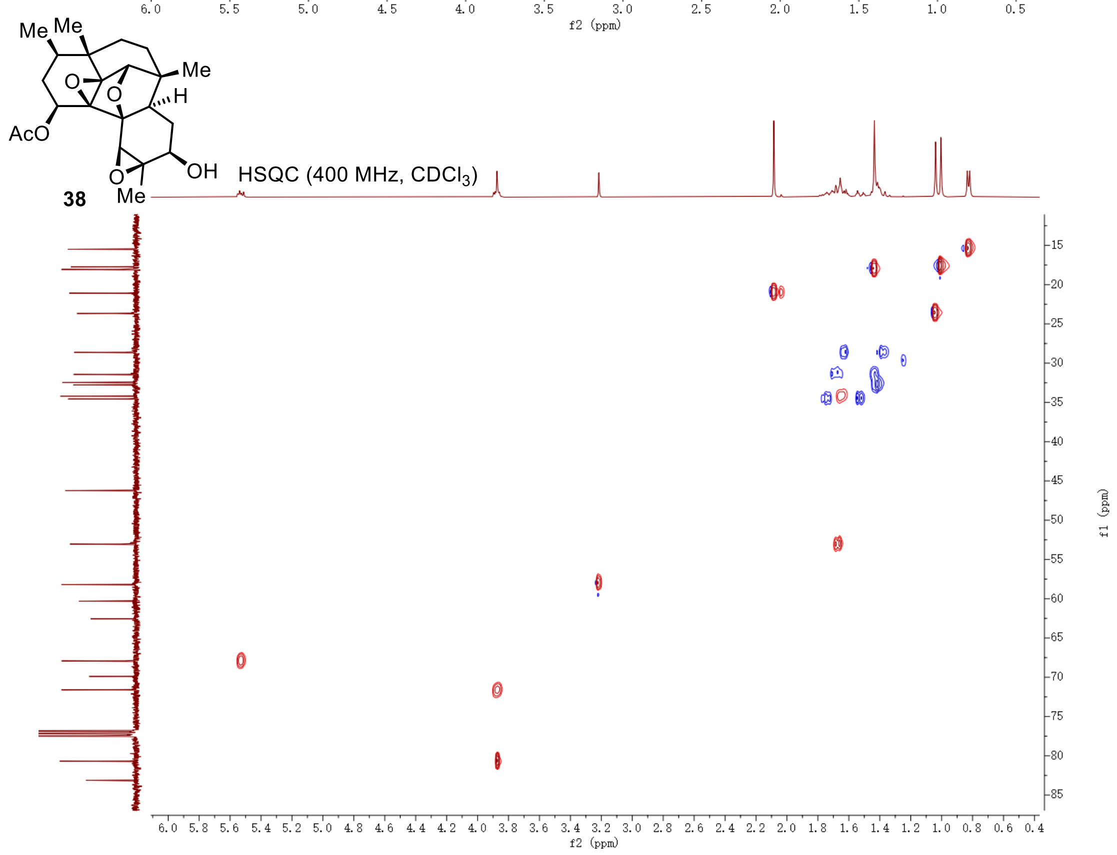
Me
Me
Me
H
O
O
AcO
O
OH
Me
38
HSQC (400 MHz, CDCl3)
f2 (ppm)
f1 (ppm)

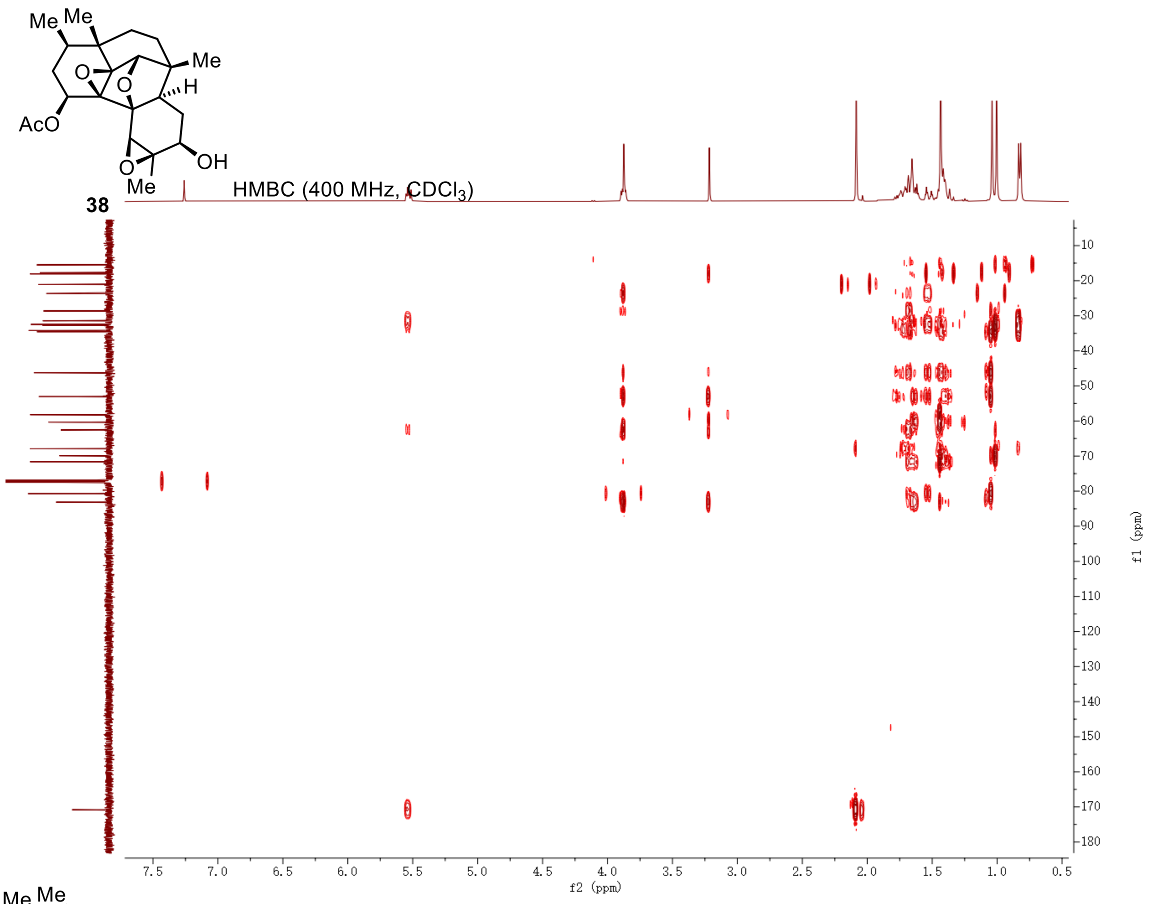
Me Me
Me
H
O
O
AcO
O
OH
Me
38
HMBC (400 MHz, CDCl3)
f2 (ppm)
f1 (ppm)

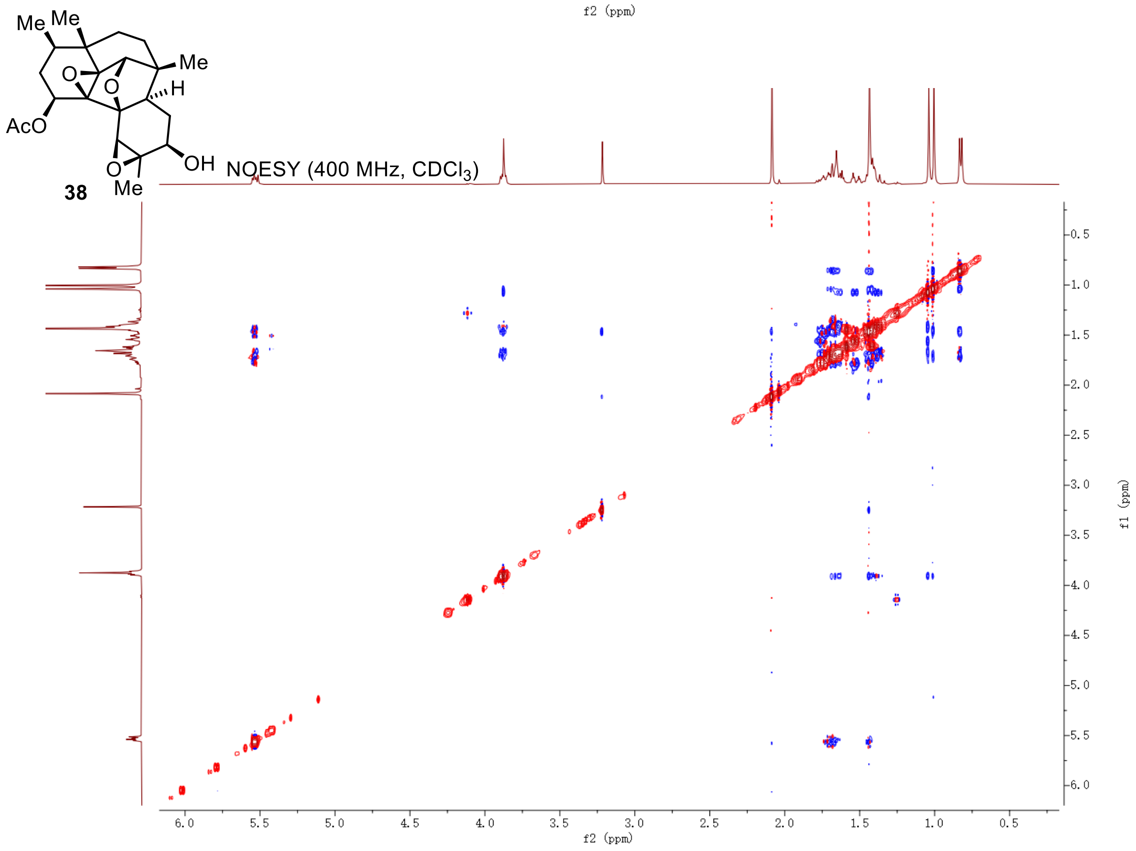
Me Me
Me
H
O
O
AcO
O
OH
Me
38
NOESY (400 MHz, CDCl3)
f2 (ppm)
f1 (ppm)

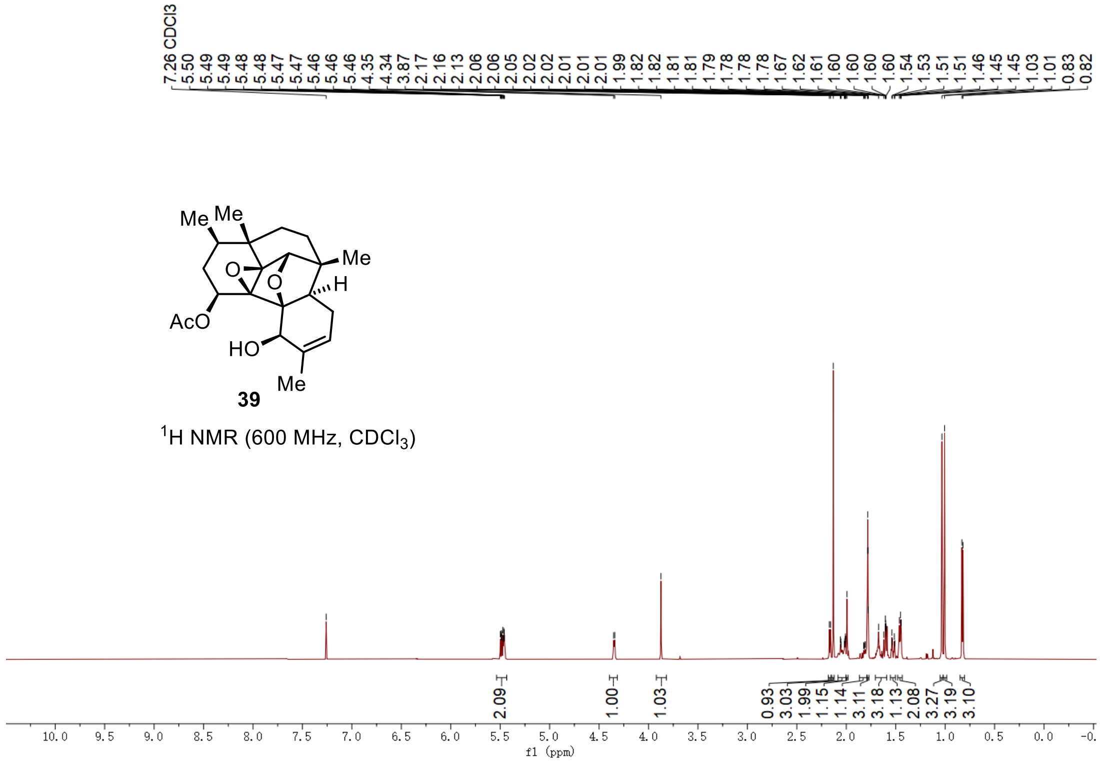

39

^{1}H NMR (600 MHz, $CDCl_3$)

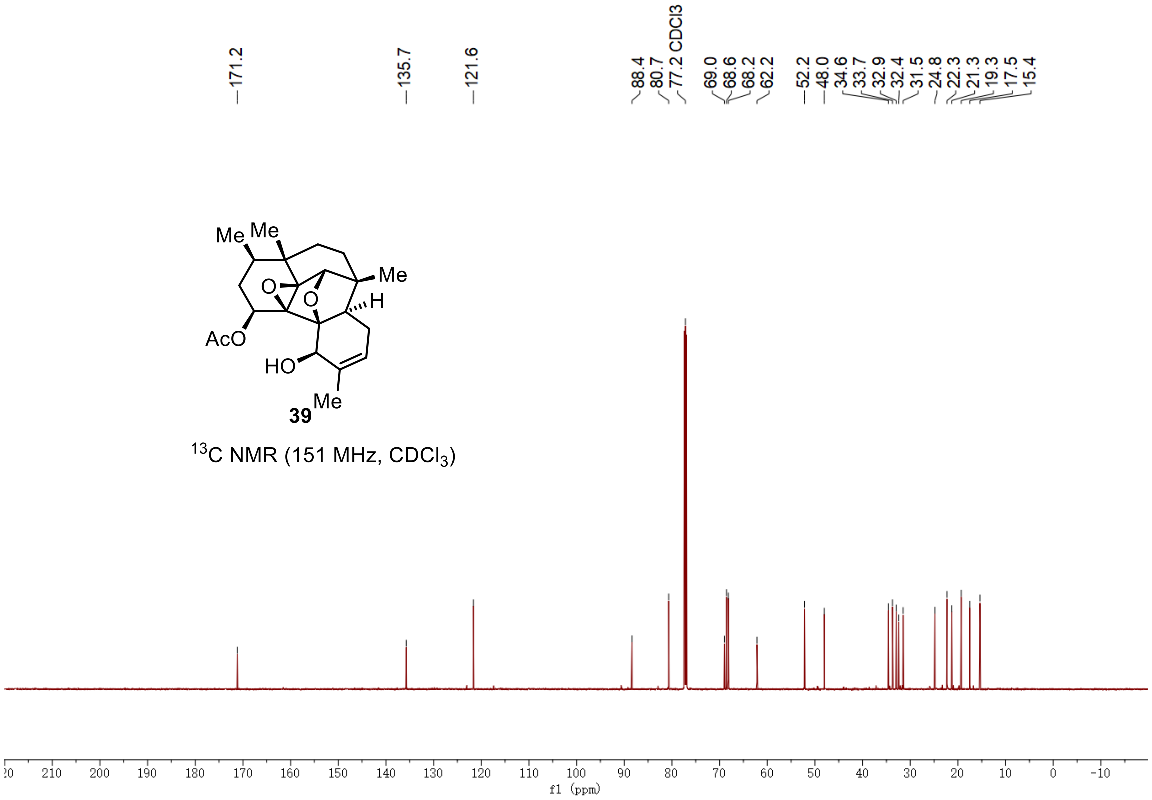

39

^{13}C NMR (151 MHz, $CDCl_3$)

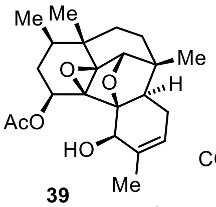

39

COSY (600 MHz, $CDCl_3$)

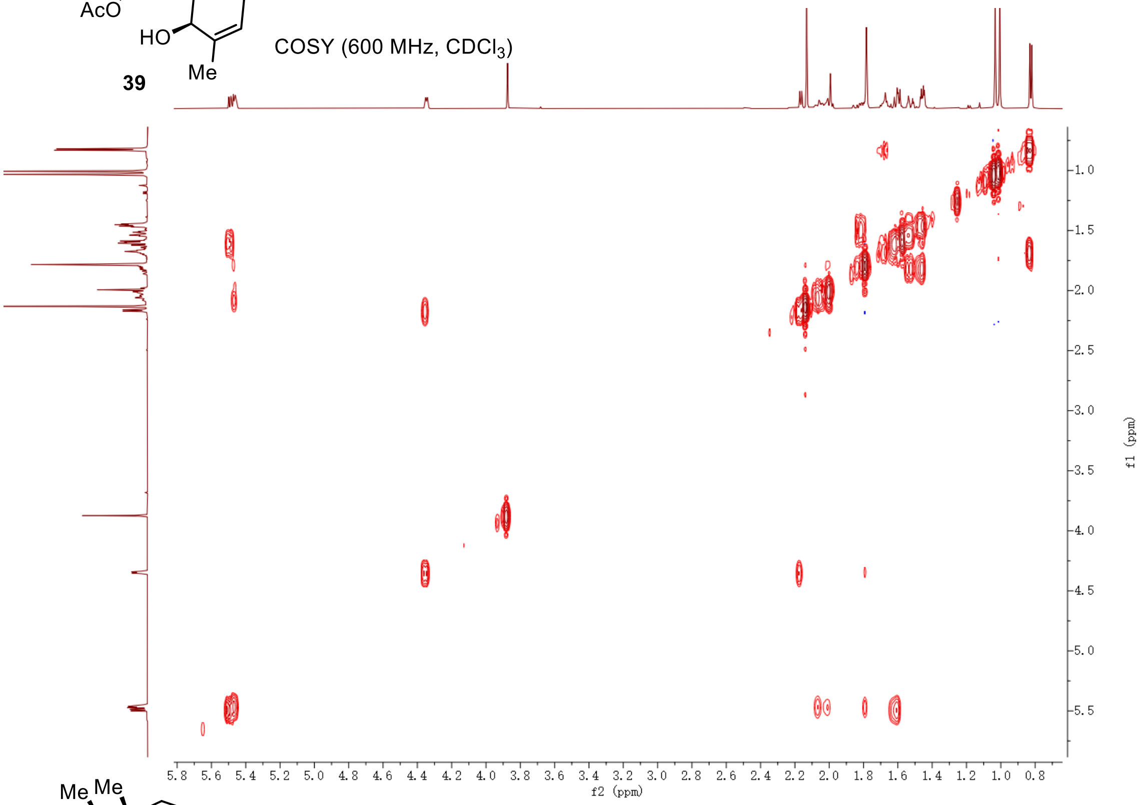

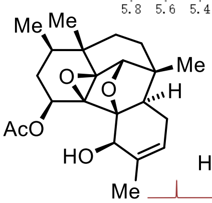

39

HSQC (600 MHz, $CDCl_3$)

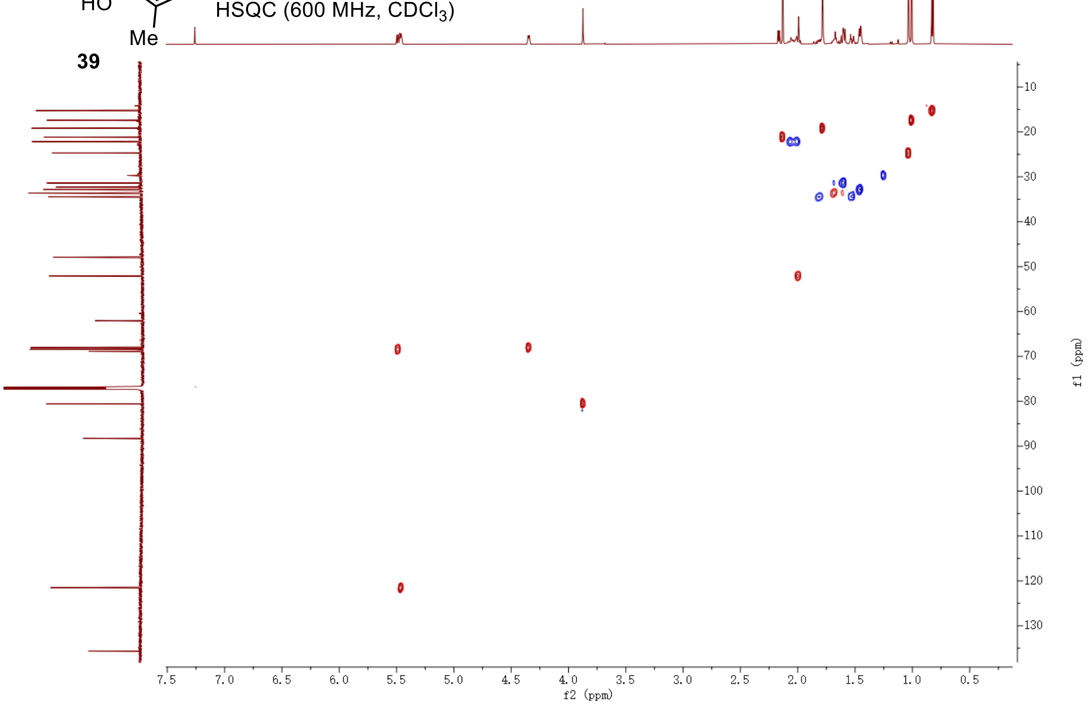

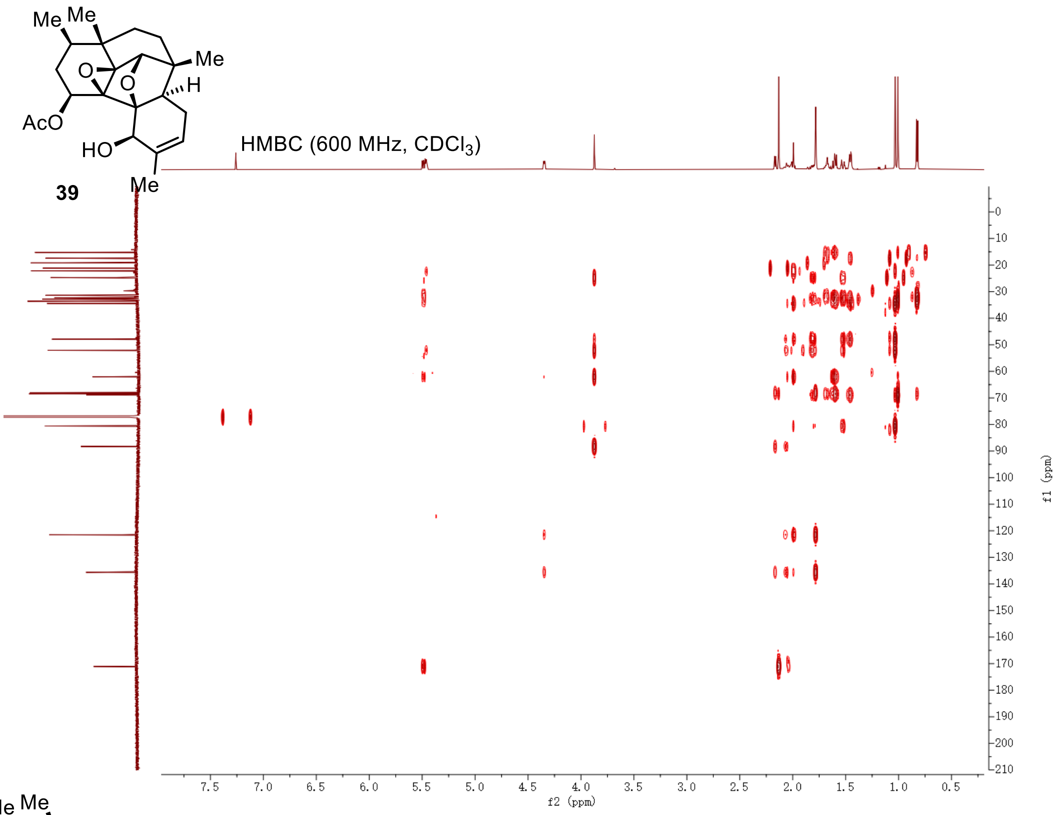
Me
Me
Me
O
O
H
AcO
HO
Me
39
HMBC (600 MHz, $CDCl_3$)
f2 (ppm)
f1 (ppm)

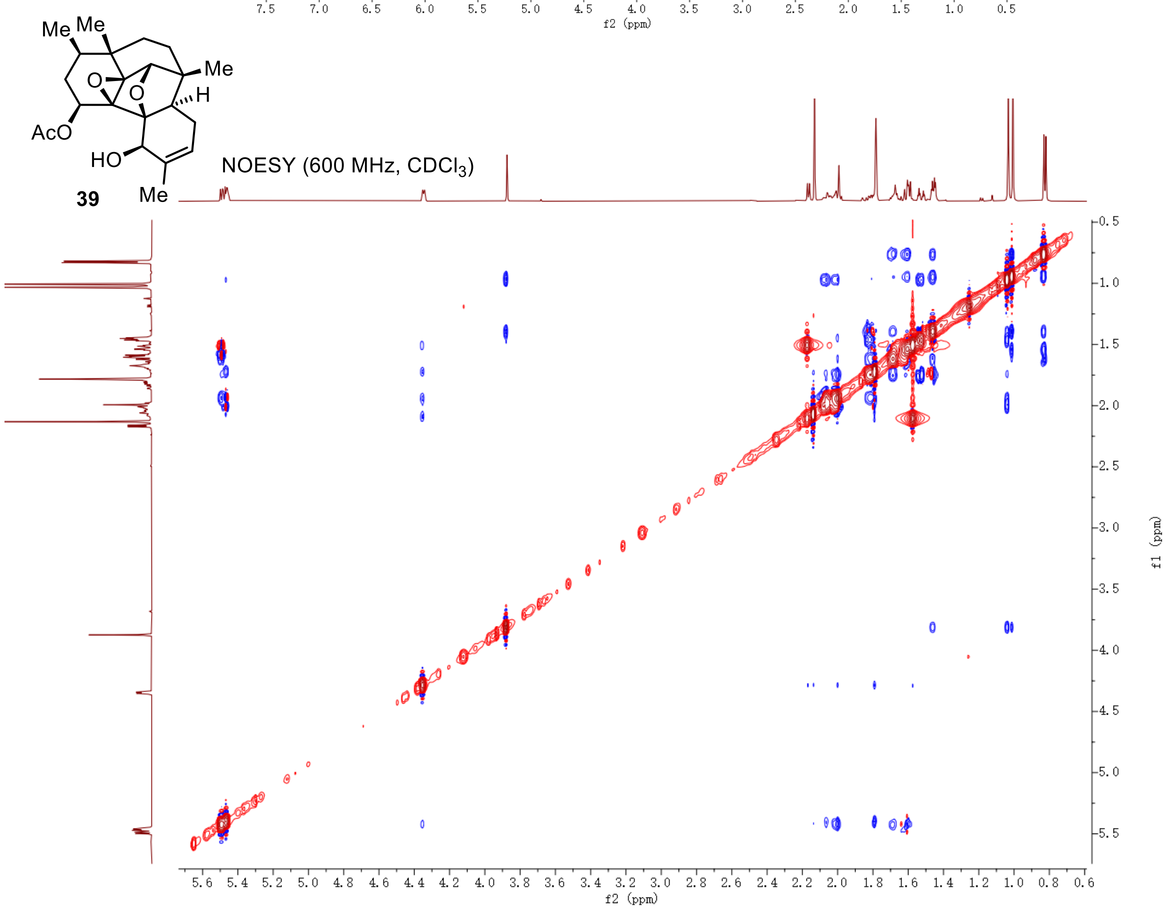
Me
Me
Me
O
O
H
AcO
HO
Me
39
NOESY (600 MHz, $CDCl_3$)
f2 (ppm)
f1 (ppm)

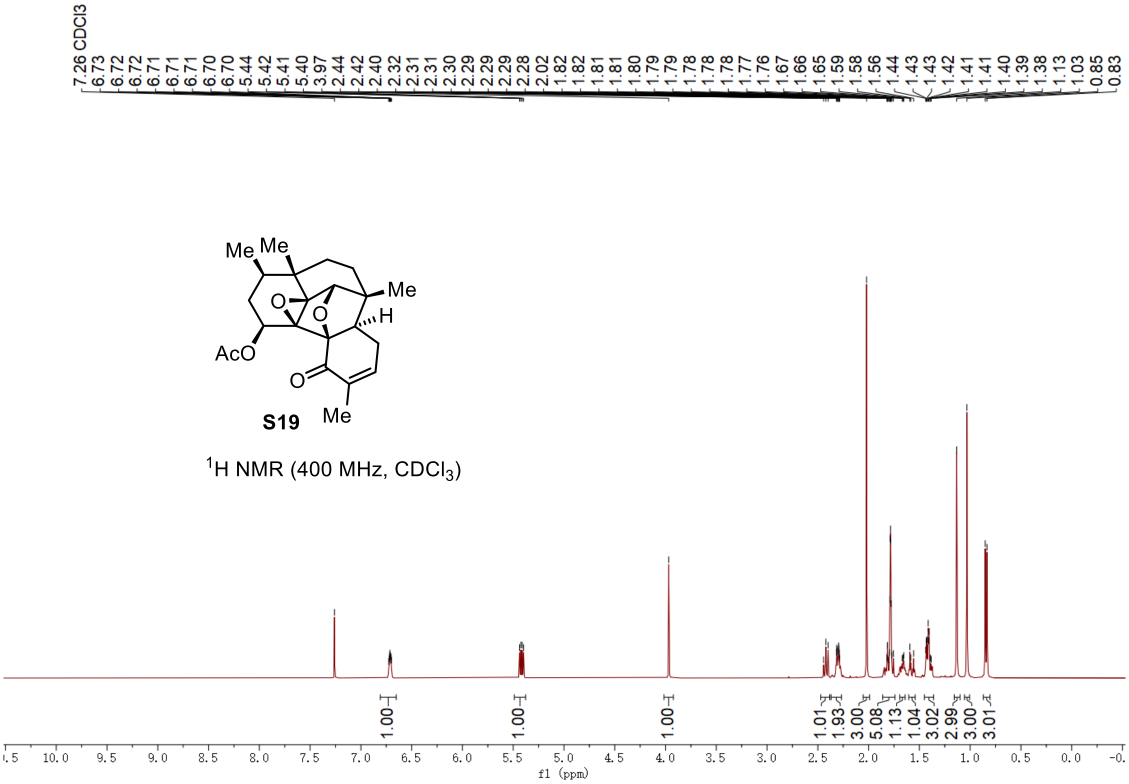

S19

^1H NMR (400 MHz, $CDCl_3$)

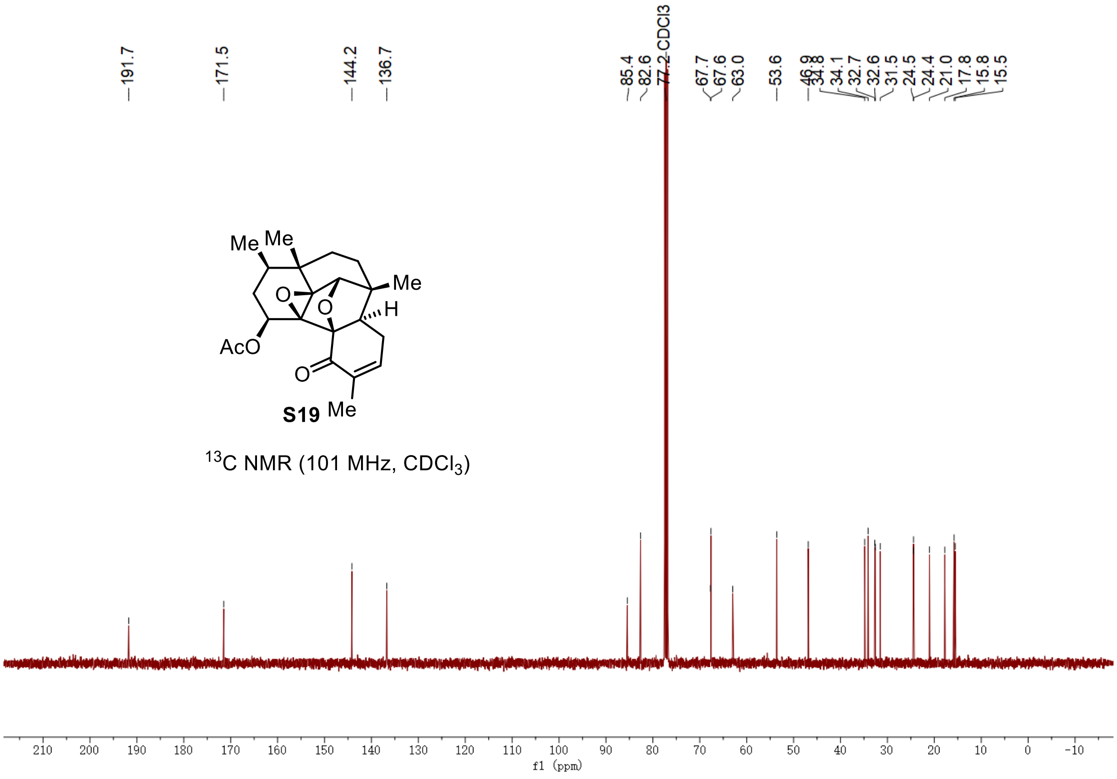

S19

^{13}C NMR (101 MHz, $CDCl_3$)

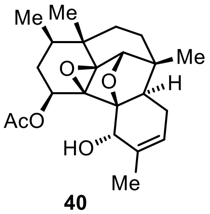

40

^{1}H NMR (400 MHz, $CDCl_3$)

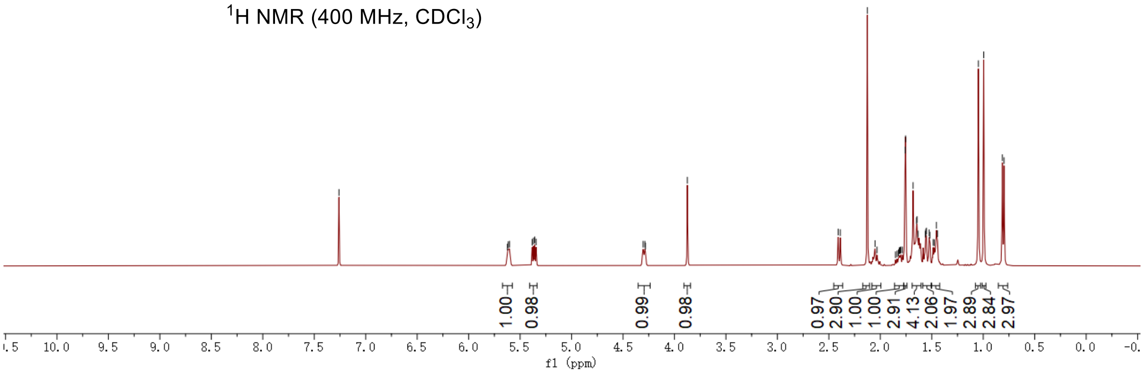

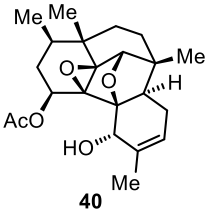

40

^{13}C NMR (101 MHz, $CDCl_3$)

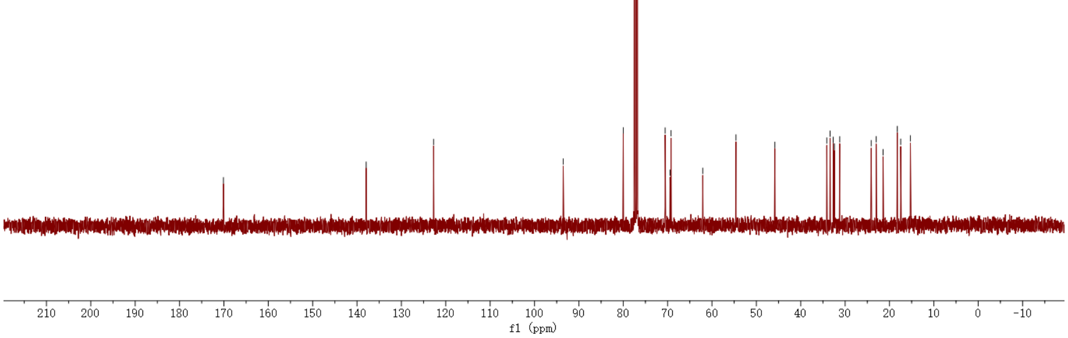

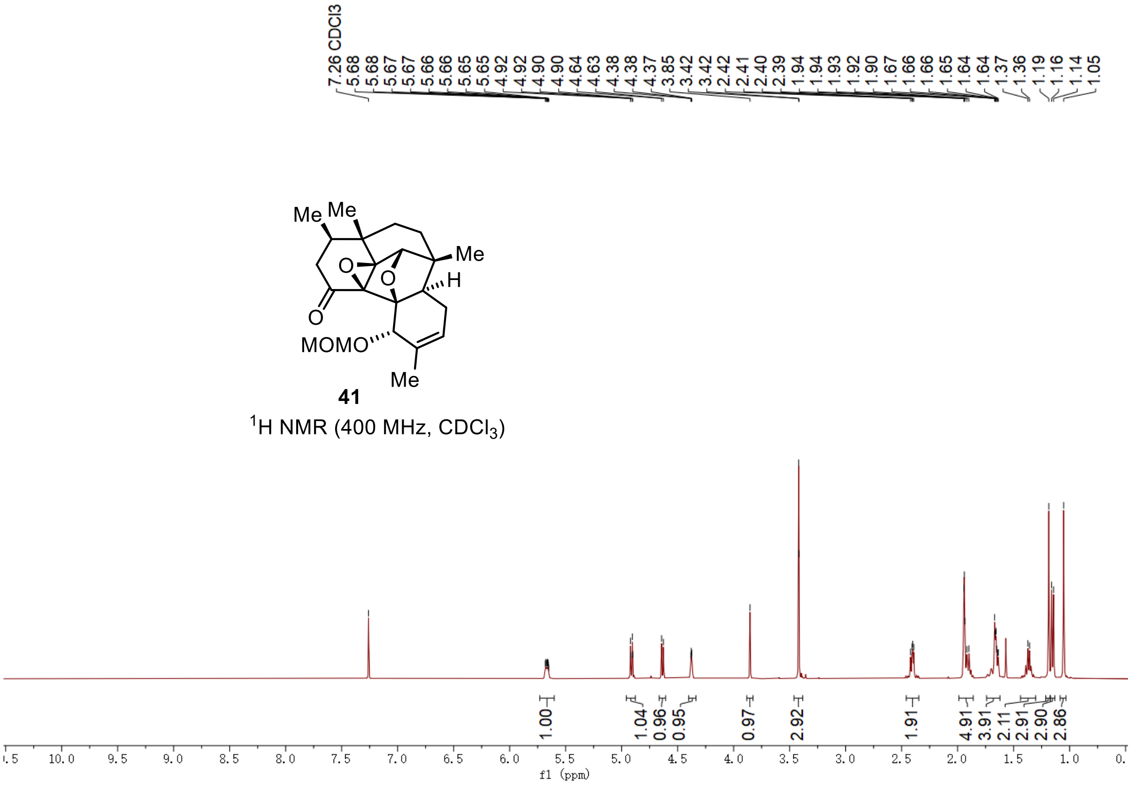

41

^{1}H NMR (400 MHz, $CDCl_3$)

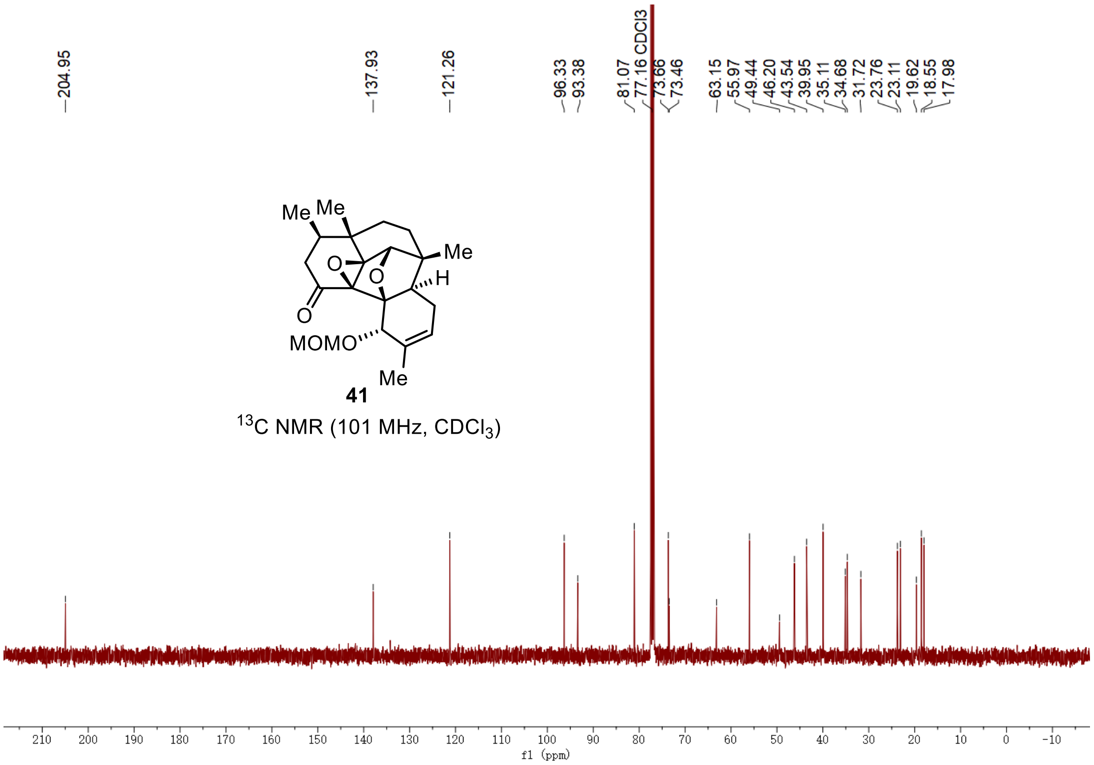

41

^{13}C NMR (101 MHz, $CDCl_3$)

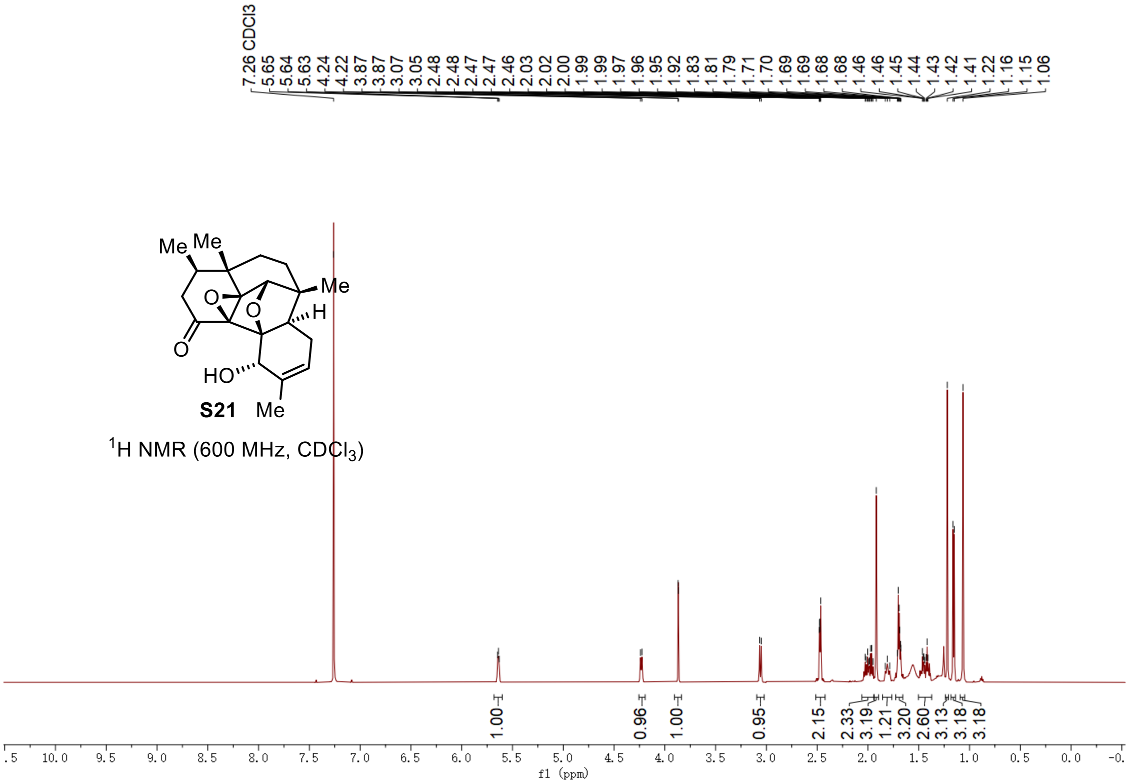

^{1}H NMR (600 MHz, $CDCl_3$)

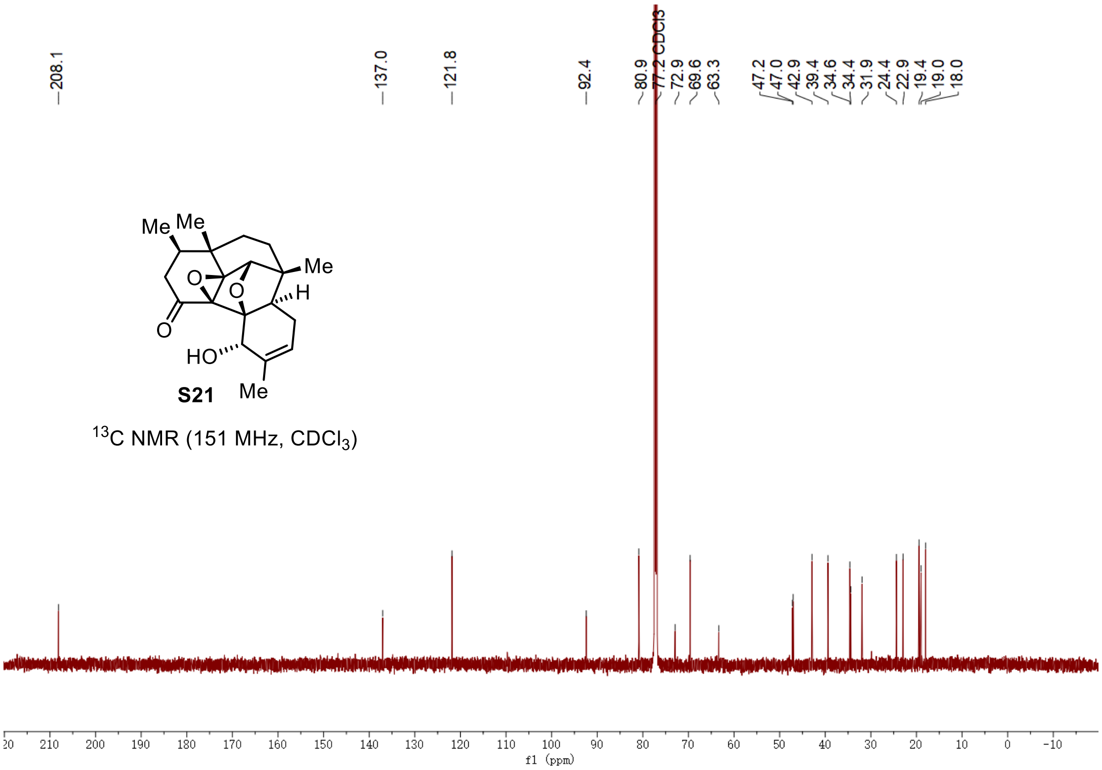

^{13}C NMR (151 MHz, $CDCl_3$)

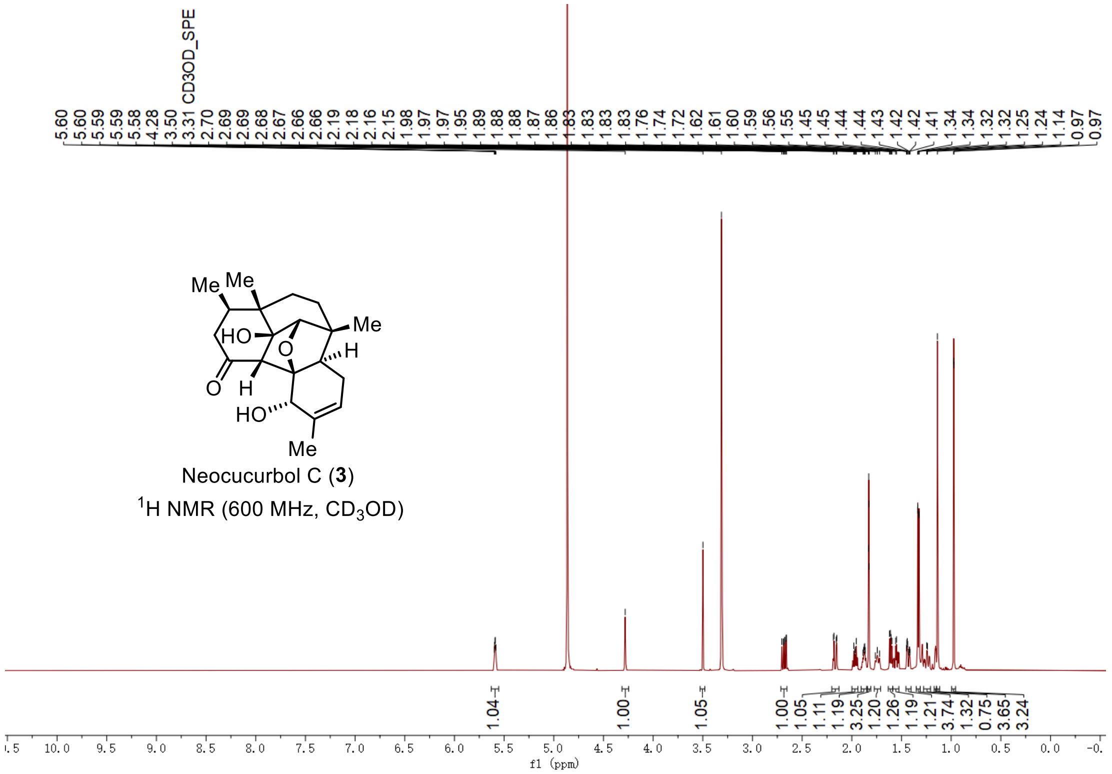

Neocucurbol C (**3**)

^{1}H NMR (600 MHz, CD_3OD)

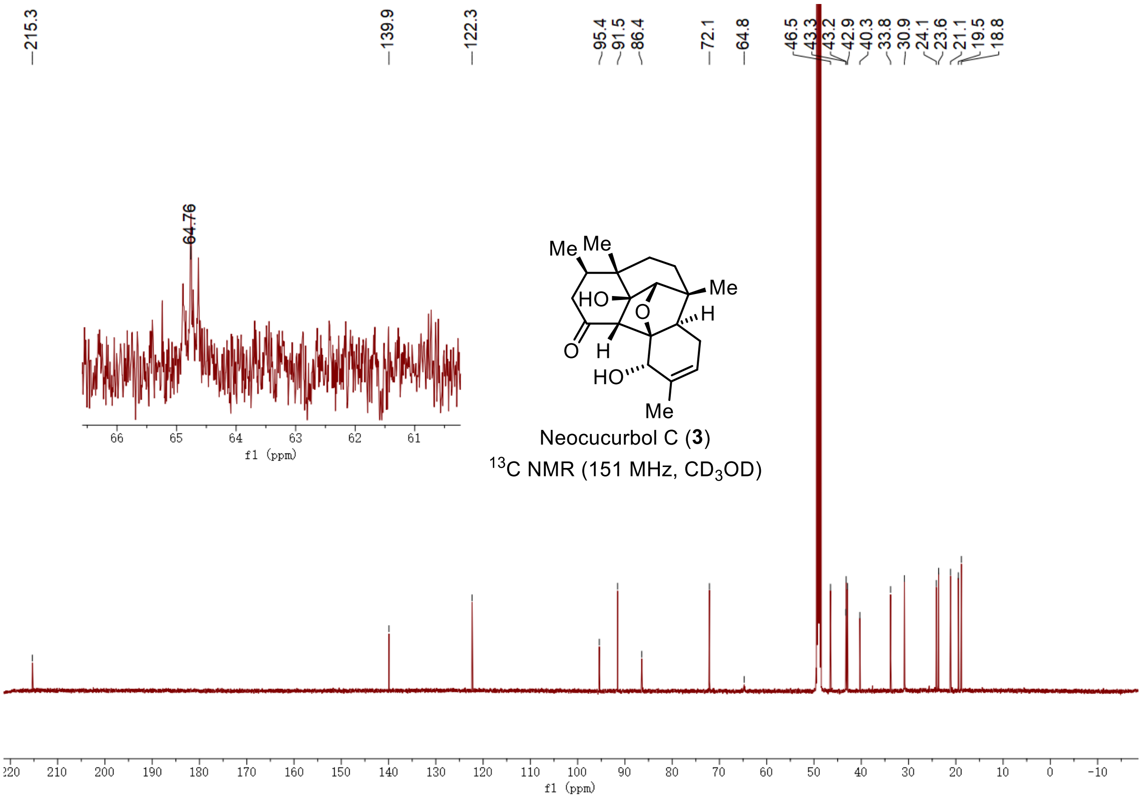

Neocucurbol C (**3**)

^{13}C NMR (151 MHz, CD_3OD)

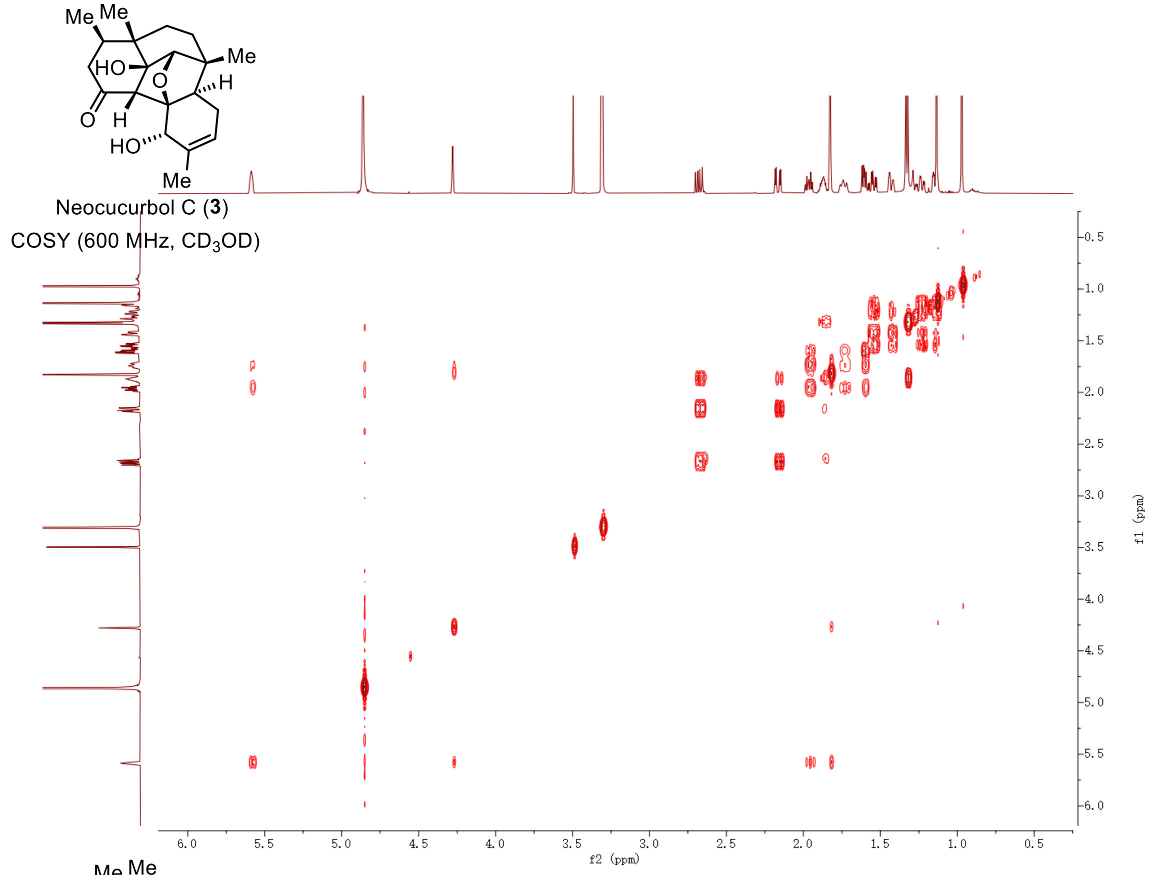

Neocucurbol C (**3**)
COSY (600 MHz, CD_3OD)

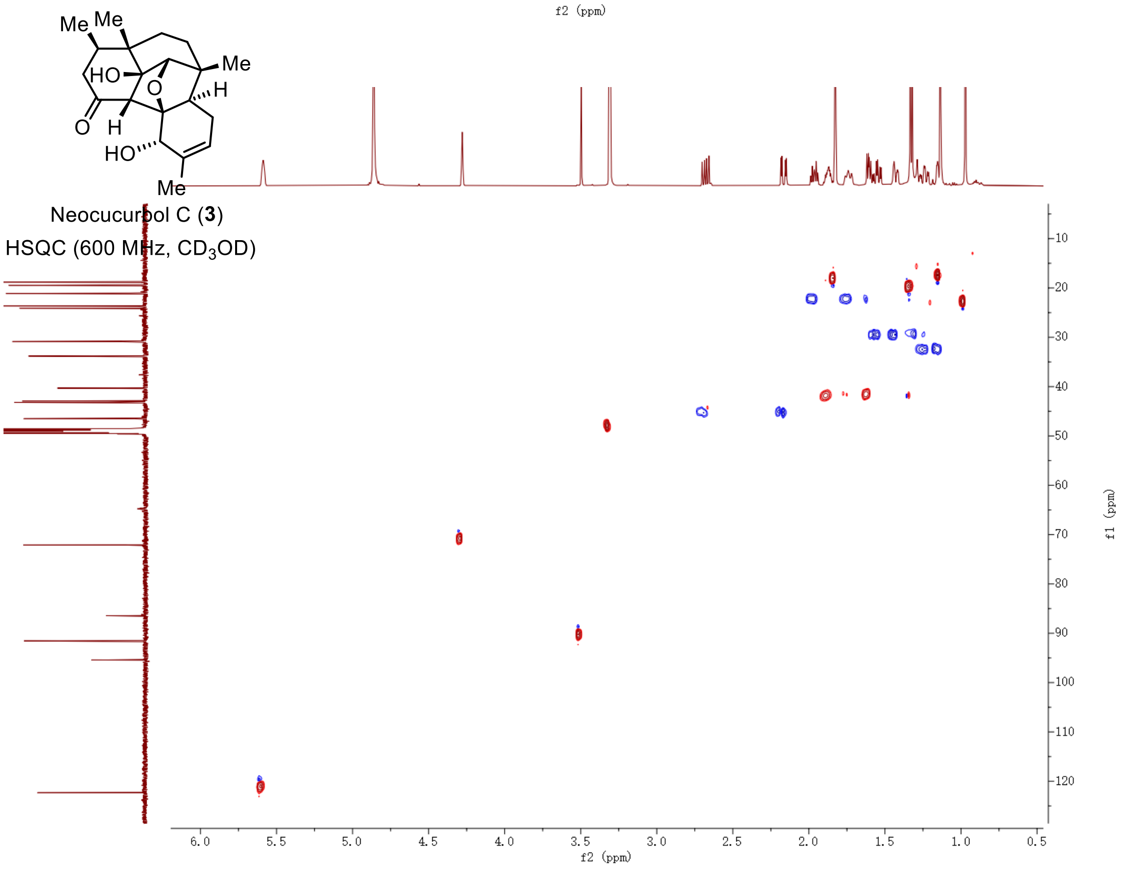

Neocucurbol C (**3**)
HSQC (600 MHz, CD_3OD)

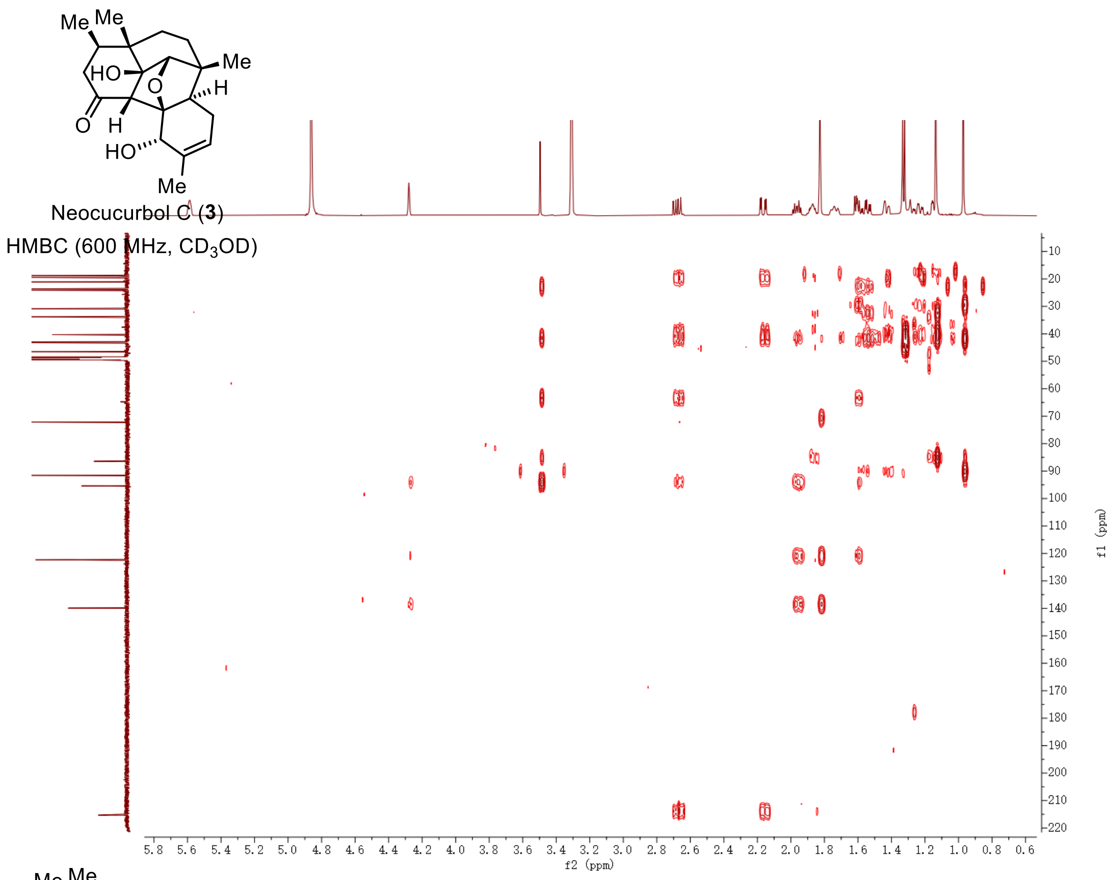
Me
Me
HO
O
Me
H
O
H
HO
Me
Neocucurbol C (3)
HMBC (600 MHz, CD3OD)
f2 (ppm)
f1 (ppm)

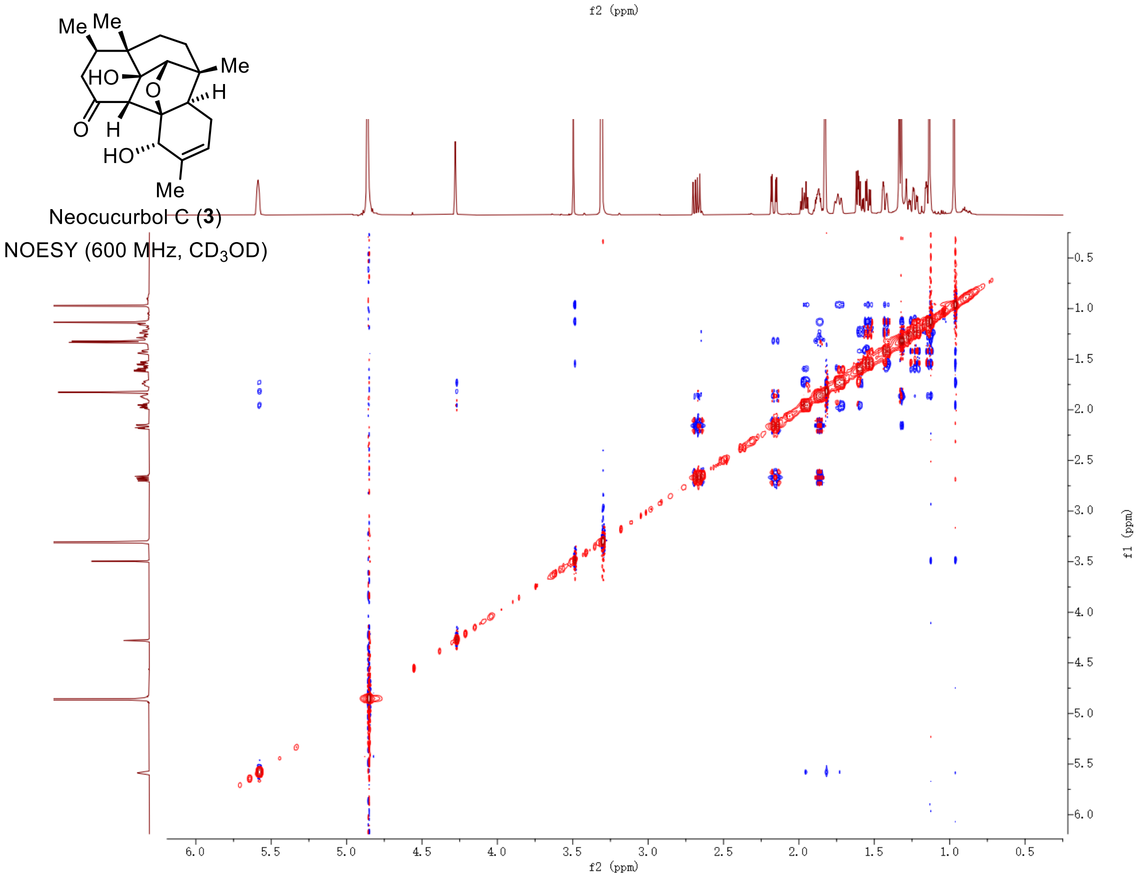
Me
Me
HO
O
Me
H
O
H
HO
Me
Neocucurbol C (3)
NOESY (600 MHz, CD3OD)
f2 (ppm)
f1 (ppm)
